# Integrative Computational Prediction Strategy for Antibody–Antigen Binding: A Case Study on Interleukin‑1 Beta

**DOI:** 10.1021/acs.jcim.6c00781

**Published:** 2026-05-27

**Authors:** Mehmet Emin Aygen, Arzu Uyar

**Affiliations:** † Department of Bioengineering, 52972 Izmir Institute of Technology, 35430 Urla, Izmir, Turkey; ‡ Computational Science and Engineering Program, Izmir Institute of Technology, 35430 Urla, Izmir, Turkey

## Abstract

We present a computational strategy to identify critical epitope/paratope residues and predict binding poses in antibody–antigen complexes. The data set consists of 14 different antibody-bound complexes with 9 diverse antigens, including a challenging target Interleukin-1β (IL-1β)—with its four distinct epitope regions—as a rigorous test case. Our strategy includes epitope/paratope site prediction for the antigen and antibody, respectively, followed by molecular docking and molecular dynamics simulations. Central to this computational strategy is essential site scanning analysis (ESSA), a fast and effective elastic network model-based method that determines binding-related essential residues in proteins. Here, ESSA ranks all residues in a given protein by their effect on the intrinsic dynamics of the protein, where the high-scored residues are considered as potential binding sites. In this study, these essential residues were then used to guide docking calculations using ClusPro, significantly improving the accuracy of antibody–antigen pose prediction compared to blind docking. Our molecular dynamics simulation results also show that ESSA-guided docking not only reproduces known binding modes with higher fidelity but also uncovers mechanistically relevant interaction patterns. However, our initial strategy was dependent on knowledge-based epitope guiding; therefore, we further investigated whether ESSA could predict epitope regions in a more automated way and developed a newer workflow, namely, EPIGUIDE, which does not require prior epitope information. EPIGUIDE was applied to 11 diverse antibody-bound complexes, and successful results were obtained. Additionally, ESSA was able to predict at least one residue in the epitope region without any prior information for the 10 cases. We also compared our results with AlphaFold3 predictions and obtained similar success rates. Our method achieves practical throughput by leveraging the efficiency of coarse-grained modeling in ESSA, bypassing the high computational cost of all-atom physics-based simulations for the initial screening. This enables rapid screening of key binding regions, making it suitable for guiding experimental validation.

## Graphical abstract

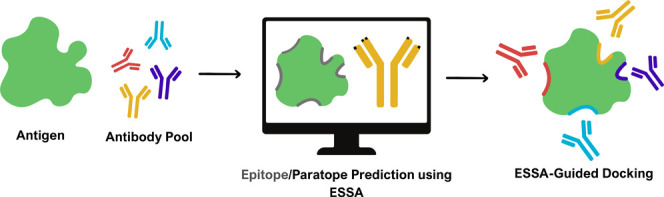

## 1 Introduction

Monoclonal antibodies (mAbs) have one of the largest shares in the global pharmaceutical market. The production and design of these therapeutics play a crucial role in the treatment of various diseases, particularly cancer and autoimmune disorders. Interleukin-1 β (IL-1β) is one of the most studied targets in oncology. Developing better mAbs targeting IL-1β requires a deep understanding of the interactions between the antibody (Ab) and IL-1β. In this context, fast and accurate prediction of binding sites in these mAbs and IL-1β structures is essential for antibody engineering. Computational methods are efficient alternatives to time- and budget-intensive traditional experiments. In this context, here we present a computational strategy that integrates essential site scanning analysis (ESSA^1^) with molecular docking to predict antibody–antigen binding poses. We first validate this approach using IL-1β and its targeting antibodies and then generate an improved version, namely, EPIGUIDE (Epitope Guided Interface Docking Engine), to demonstrate its broader applicability by benchmarking it against 11 diverse antibody-bound complexes, including the original antigen IL-1β.

Interleukin-1 beta, one of the three members of the Interleukin-1 family, is a cytokine protein encoded by the IL-1B gene and is also known as lymphocyte-activating factor. Cytokines are small signaling proteins that attach to cell surfaces and transmit important intracellular messages. This cytokine protein has a crucial role in autoimmune and inflammatory diseases. The crystal structure of IL-1β contains 154 amino acids (aa) (∼17 kDa) in its mature form. There are 12 antiparallel beta-strands forming a beta-barrel. Currently, four different antibodies with published crystal structures, namely, Gevokizumab, IgG26, AAL160, and Canakinumab,^2–5^ target IL-1β via binding to distinct epitope regions, as shown in Figure 1. IgG26 binds to IL-1β and blocks interactions with its receptor and accessory protein. AAL160 and Canakinumab bind overlapping regions on IL-1β but adopt distinct poses. On the other hand, Gevokizumab targets a site opposite the binding site of IgG26 and allosterically modulates IL-1β activity by reducing its binding affinity to the receptor complex.

**Figure 1:**
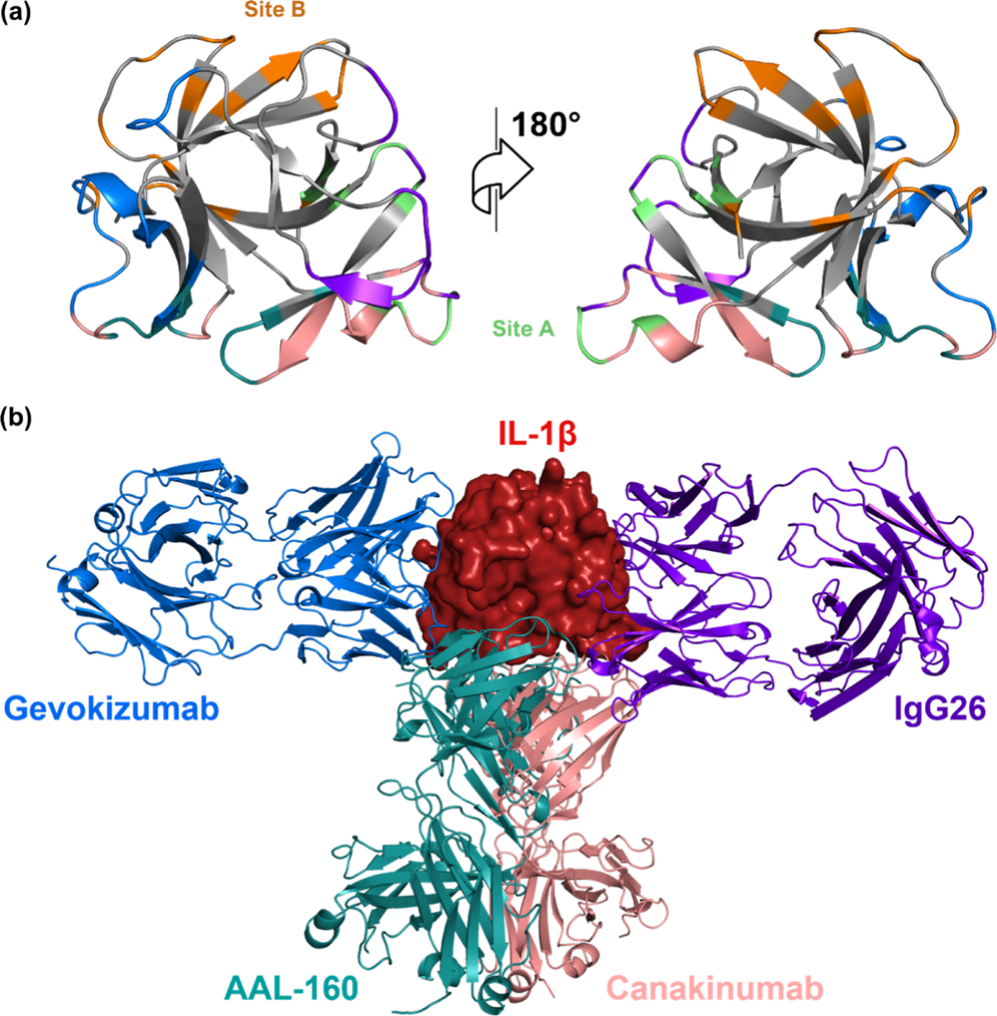
Human IL-1β and four different antibody:IL-1β crystal structures. (a) The known binding sites of IL-1β: Gevokizumab binding site (blue, 4G6M); IgG26 binding site (purple, 7CHY); AAL-160 binding site (teal, 7Z4T); Canakinumab binding site (salmon, 4G6J); and IL-1R binding sites A (lime) and B (orange). (b) Only the IL-1β (red) of Gevokizumab-bound IL-1β (4G6M) is shown here for clarity. All figures are prepared using PyMOL v2.5.4 ^6^.

Three-dimensional structures of proteins, both in apo and bound forms, can have many different conformations due to their dynamicity. Additionally, these forms may exhibit different allosteric behavior. There are studies showing the existence of allostery in antibodies,^7–9^ which is consistent with the long-speculated notion that all proteins are allosteric.^10,11^ The identification of key binding sites, including allosteric ones, may contribute to the development of potent and targeted mAbs. It is well-known that the complementarity-determining regions (CDRs) are the interaction sites of Abs, and their sequences and conformations vary for different antigens (Ags). Particularly, the conformational diversity of HCDR-3 loops in the heavy chain has been shown to have a significant impact on antigen recognition and binding.^12^ However, dynamics and allostery in the Fab region of IL-1β-targeting mAbs have not yet been studied. Here, we focus on identifying key sites and residues in IL-1β, as well as the Fab regions of four different IL-1β-targeting antibodies, with a special emphasis on the HCDR-3 loops in the studied mAbs, considering both antigen and antibody dynamics.

Current experimental methods provide valuable insights into antibody development.^13,14^ However, due to the high cost and time requirements of experiments, several computational tools, specifically focused on antibody design and discovery, have been developed, including physics- and machine-learning-based methods.^15–20^ Each computational tool has its own advantages and limitations. Today, with the help of recent advanced computational tools, it is easier to model both the antigen and antibody, as well as the complex structure; however, it remains challenging to find the correct binding pose of an Ab/Ag complex. Considering the dynamic nature of proteins, including explicit/hidden allosteric effects, which is valuable for epitopes that depend on tertiary structure or are poorly conserved in linear sequence, would be helpful in identifying the paratope and epitope residues and speeding up the prediction of complex structures. For this purpose, we used ESSA, a fast and accurate binding site prediction tool, to determine critical residues on paratopes and epitopes. To the best of our knowledge, this is the first time that ESSA has been used to predict paratope regions, and its ability to answer the questions below was evaluated.

This study aims to answer the following questions: Are there any differences in binding sites in the antigen and antibodies before and after the complex formation? Can ESSA identify residues that play a critical role in antibody:IL-1β interactions, especially on CDR-3s? Do essential residues identified by ESSA improve antibody:IL-1β docking results and yield better binding pose predictions? To find answers, we selected IL-1βs along with four different mAbs. Upon completing the preparation of these structures, we analyzed them as a complex and individually using the “ESSA” to predict potential and known interacting residues. In addition, the ClustENMD conformational sampling method was applied to the least-performing prediction case, and the selected conformers were investigated using ESSA. After the analyses, we used ClusPro to perform protein–protein docking in three different ways for each Ab: Ag system studied, to demonstrate the impact of critical findings from ESSA on improving the docking results. The stability of the docking poses was also investigated using conventional molecular dynamics (MD) simulations.

The calculations described above addressed our initial questions about IL-1β systems, but they also revealed a key limitation: the need for prior knowledge of the epitope. This raised a new question: could ESSA be used to predict epitope footprints without any prior knowledge, and, if so, would this approach generalize to other antigens? To answer this, we developed a complete blind prediction workflow, namely, EPIGUIDE, and tested it on 11 diverse antibody–antigen complexes (including the original IL-1β system). For each complex, ESSA predicted epitope regions directly from the antigen structure, and these predictions were used to constrain docking calculations. We then compared the resulting poses against experimental structures to evaluate the method’s accuracy and generalizability.

## 2 Materials and Methods

### 2.1 The Data Set and Workflows

Fourteen different antibody-bound antigen complex structures were selected from “Protein Data Bank”^21^ based on four criteria: (i) the source organism is *Homo sapiens*, (ii) the experimental method is X-ray diffraction, (iii) having a resolution value less than 3.50 Å, and (iv) containing no mutation. Information about the proteins in the data set is given in Table 1. Four different antibody-bound IL-1β, which is the rigorous case in the data set, are given in Figure 1. The other protein structures were introduced in the Results and Discussion section, where predicted docking poses were provided. The crystal structures of the antibodies studied have only the Fab region, have no mutations, and were produced in vitro. Antibody/antigen (Ab/Ag) interactions in each complex were determined using the PDBsum database,^22^ and interacting residues were used to analyze and compare ESSA results.

**Table 1:** 14 Diverse Antibody–Antigen Complexes

PDB_ID	Antigen	Ag residue #	Antibody
4G6J	IL-1β	149	canakinumab
4G6M		150	gevokizumab
7CHY		150	IgG26
7Z4T		152	AAL160
3WD5 ^23^	TNFα	152	adalimumab
4G3Y^24^		148	infliximab
5Y9J^25^	TNFSF13B	144	belimumab
5GGT^26^	PD-L1	116	BMS-936559
7C88 ^27^		116	JS003
1OAK^28^	VWF	195	NMC-4
3S36 ^29^	VEGF	112	1121B
4DGI^30^	prion	97	POM1
7E9B^31^	PD-1	114	HLX10
8Y31 ^32^	IFNAR1	102	QX006N

This study includes two different workflows, as given in Figure 2. A knowledge-based approach was applied only to four different antibody-bound IL-1β systems (Figure 2A). Experimentally known epitope residues were used as constraints in protein–protein docking (ClusPro) to assess whether ESSA-guided docking could recover correct antibody–antigen binding poses. Resulting poses were validated against crystal structures and assessed for stability using MD simulations.

**Figure 2:**
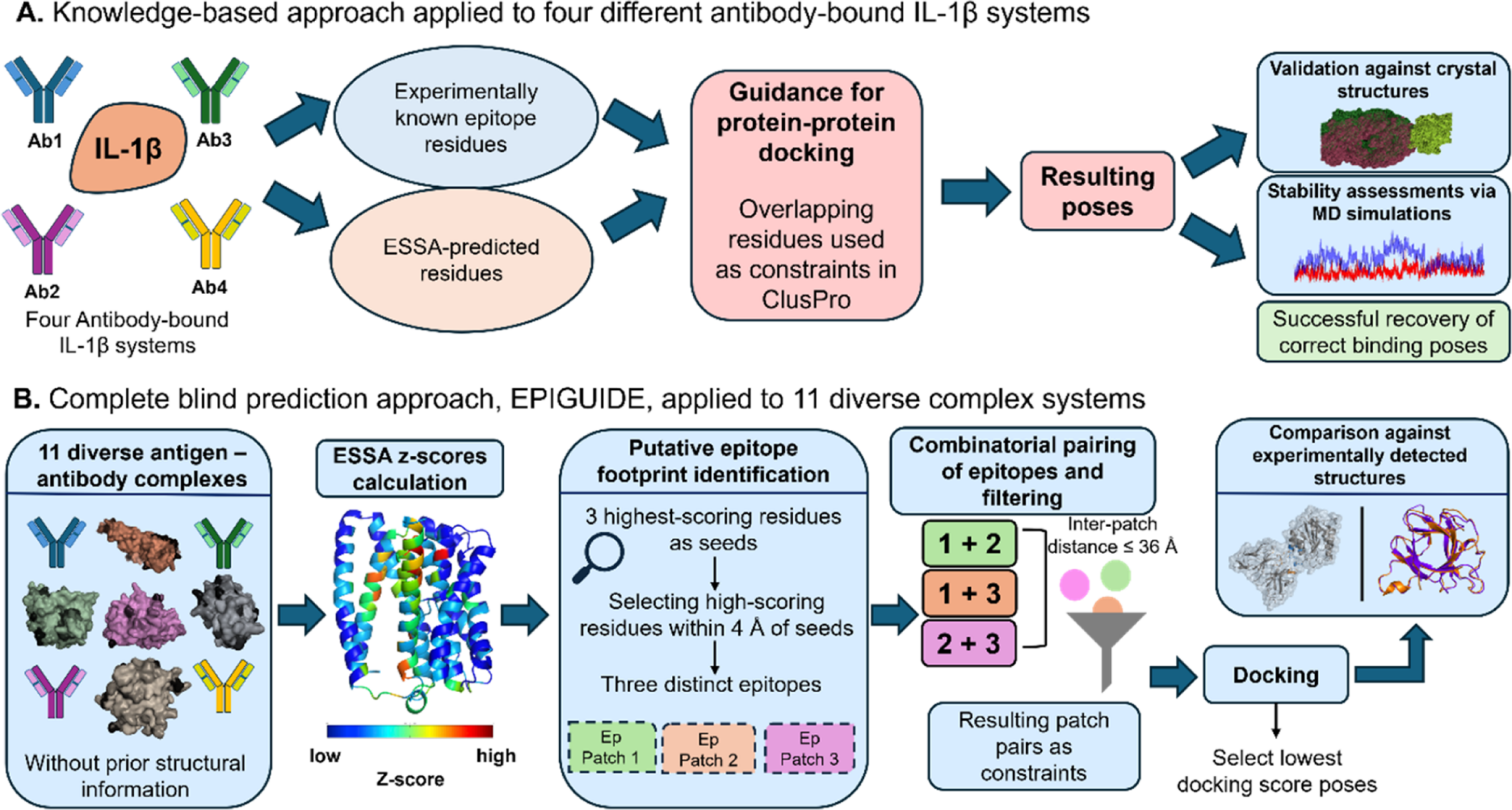
Workflows for the development and validation of the ESSA-guided antibody–antigen docking strategy. (A) Knowledge-based approach and (B) EPIGUIDE, a complete blind prediction approach.

As the second workflow, the complete blind prediction approach, EPIGUIDE (Epitope-Guided Interface Docking Engine), was applied to 11 diverse antibody-bound complexes, including IL-1β (Figure 2B). For each antigen, ESSA *z*-scores (please see the next sub-section 2.2 for more details about the method) calculated with three different *R*_cut_ values (7.3 Å, 10 Å, and 13 Å) were used to identify putative epitope footprints without prior structural information. First, three residues were determined to be used as seeds in putative epitope formation. Here, two different seed-determining approaches were followed: “Max” and “Ave”. In the “Max”, the *z*-scores obtained from ESSA using 3 different *R*_cut_ values and 20 modes each (in total 60 modes) were sorted from highest to lowest, and the three residues with the highest *z*-scores were selected as seeds. In the “Ave”, if a residue had the highest *z*-score in different modes, its average *z*-score was calculated, resulting in a unique *z*-score for each residue. These values were again sorted from highest to lowest, and the three highest values were accepted as seeds. For each seed, we identified all neighboring residues with *z*-scores higher than 5 and residing within 4 Å of the seed, thereby constructing three spatially clustered epitope patches. These three patches were then paired combinatorially to define a larger region suitable for docking. However, to reflect the physical constraints of antibody recognition, we imposed an interpatch distance threshold between the centers of the two patches; only pairs satisfying this criterion were advanced to the docking stage. Here, the interpatch distance threshold was defined using the average distance between the heavy and light chains of 117 various antibodies using the distance between Cα atoms of residue 65 in the heavy chain and residue 55 in the light chain. The average distance between the heavy and light chains was obtained as 34 ± 2 Å; therefore, an interpatch distance ≤36 Å was used as a threshold. Three epitope patches (Ep1, Ep2, and Ep3) were paired as Ep1 + Ep2, Ep1 + Ep3, and Ep2 + Ep3. The epitope pairs with an interpatch distance ≤36 Å were used as the target in docking calculations. Dockings were performed for the epitope pairs coming from both “Max” and “Ave” seed-determining approaches. If the epitope pairs are exactly the same for the Max and Ave approaches, then only one docking calculation was performed for such cases. The resulting Ab/Ag poses obtained using two different seed-determining approaches were separately sorted by their docking score. Here, only the poses interacting with CDRs from the antigen were used. The pose with the lowest docking score was selected as the potential antibody–antigen binding pose and compared against experimentally determined complex structures to assess prediction accuracy across the benchmark set. However, three more evaluation methods were also tested, as explained in Section 3.6 within the Results and Discussion part.

### 2.2 Essential Site Scanning Analysis

The ESSA^1^ was used in this study to monitor changes in allosteric sites in antigen and antibody structures before and after complex formation. ESSA is an Elastic Network Model-based methodology in which the side-chain atoms of each amino acid are added to the elastic network one by one to mimic a molecule binding. Each addition has a small or significant effect on harmonic mode frequency values compared to the original network that was constructed using only C-alpha atoms. These difference values are then converted into a statistical *z*-score value for each amino acid. At the end of these calculations, the amino acids whose side chains caused the most shift in mode frequency values pinpoint essential binding sites. The ESSA was applied separately to IL-1β antigen, its antibodies, and the corresponding IL-1β–antibody complexes, and the results were compared with each other. In this way, it was possible to determine where the changes in the antigen and antibodies studied occurred upon complex formation. For each structure, three different *R*_cut_ values (7.3 Å, 10 Å, and 13 Å) were considered, and 20 harmonic slow modes were used in ESSA. For each amino acid, the highest *z*-score value obtained for that amino acid was accepted and used regardless of the cutoff value. We named this approach “combined-cutoff ESSA”. *Z*-score values are normalized to a range of 1–7 for accurate comparison of structures. In this range, 6 (orange) and 7 (red) denote the most probable essential binding sites in a structure.

### 2.3 Conformational Sampling Using ClustENMD

ClustENMD^33^ is an Elastic Network Model-based method and is used for all-atom conformational sampling. In this method, a protein structure is first deformed along the selected normal modes (harmonic slow modes) obtained from the Anisotropic Network Model,^34^ where they are randomly weighted in the calculation. After deformation, new conformers are produced, and the energy of these conformers is minimized via short MD simulations. Finally, all-atom structures are obtained. Here, we considered the first five harmonic slow modes and set the number of generations (gens) to 5 in ClustENMD to generate 300 conformations in total for each apo antibody (antigen-removed) structure. Then, three frames with the highest RMSD values relative to the reference structure were selected within 300 frames and subjected to ESSA analysis for binding site prediction. On the other hand, for the Ab/Ag docking poses and the crystal structure of Gevokizumab: IL-1β, ∼2000 conformers were generated using 5 harmonic slow modes and 10 gens.

### 2.4 Molecular Docking Using ClusPro

ClusPro is a freely available and fully automated web server for protein–protein docking, which is based on the PIPER docking program.^35,36^ Potential binding poses are calculated for the two protein structures. ClusPro first performs a rigid body docking, where both structures are kept rigid, to sample billions of different conformations (poses). 1000 conformations with the lowest energy values are then selected for clustering based on RMSD values. The largest clusters, in general, reveal the most probable docking poses of the two proteins. Finally, energy minimization is applied for the poses using the CHARMM force field^37^ to prevent steric clashes in the protein–protein complex. In addition to blind docking in ClusPro, critical residues that are determined either experimentally or computationally can be specified as either attraction or repulsion sites. Therefore, in this study, we performed both blind and guided docking for the antibody–antigen pairs. The coordinates of the antibody and antigen in the complex crystal structure were directly used in blind docking. In guided docking, the critical residues identified by the ESSA were used as attraction sites. Two different guided docking calculations were performed using essential residues on CDR-3 and only on the antigen. All guided docking results were compared to blind docking and the reference crystal structure in terms of energy and surface complementarity.

### 2.5 MD Simulations

All-atom conventional MD simulations were performed for blind and guided docking poses of the selected complex structure. The dynamics and stability of the docked complex structures were investigated along with the original hydrogen bond formations. CHARMM-GUI^38^ was used to prepare complex systems in TIP3 water with a box edge distance of 12 Å. The simulation box dimensions were 133 × 133 × 133 (*x*, *y*, and *z* axes) and neutralized with 0.15 M KCl ions using the Monte-Carlo method. The Ag_ESSA system comprises ∼223,500 atoms, while the Blind system has a total of ∼244,000 atoms. Each system was equilibrated for 2 ns with positional restraints on protein heavy atoms (backbone: 400 kJ/mol/nm^2^, side chains: 40 kJ/mol/nm^2^) using a 1 fs time step using the *NVT* ensemble at 303,15 K. Long-range electrostatic interactions were treated using the Particle Mesh Ewald method with an error tolerance of 0.0005. van der Waals interactions were smoothly switched off between 10 and 12 Å using a force-switching function. All bonds involving hydrogen atoms were constrained using the SHAKE algorithm. Simulations were performed in the *NPT* ensemble at 303,15 K using Langevin dynamics (friction coefficient 1 ps^–1^) and at 1 bar pressure using a Monte Carlo barostat with isotropic coupling. Coordinates were saved every 20 ps for analysis. Three replicate MD simulations, each of 300 ns, were performed for the docked (Ag_ESSA and Blind) complexes using OpenMM^39^ toolkit with the CHARMM36m^40^ force-field. In total, 15,000 frames were acquired for each MD simulation. Afterward, the frequency of specifically formed hydrogen bonds (formed in the original crystal structure) between Ag/Ab was calculated and compared between each frame of the simulations.

## 3 Results and Discussion

### 3.1 ESSA Identifies Essential Residues in the Epitope Regions of IL-1β

We individually analyzed four different antibody-bound complex structures of IL-1β (Figure 1) using ESSA to identify the essential sites in IL-1β where the eigenvalues of the global modes change upon antibody binding. Due to interactions with different mAbs, there is a slight conformational change in the structures of selected IL-1βs. Figure S1 and Table S1 display the alignment of IL-1βs, along with their RMSD values. First, we removed the antibodies from each complex structure and performed ESSA with three different cutoffs. Then, we investigated the ESSA results for varying cutoffs (Figure 3) using a combined approach as explained in the Materials and Methods section. Combined-cutoff ESSA results of all Ags are shown in Tables S2–S5.

**Figure 3:**
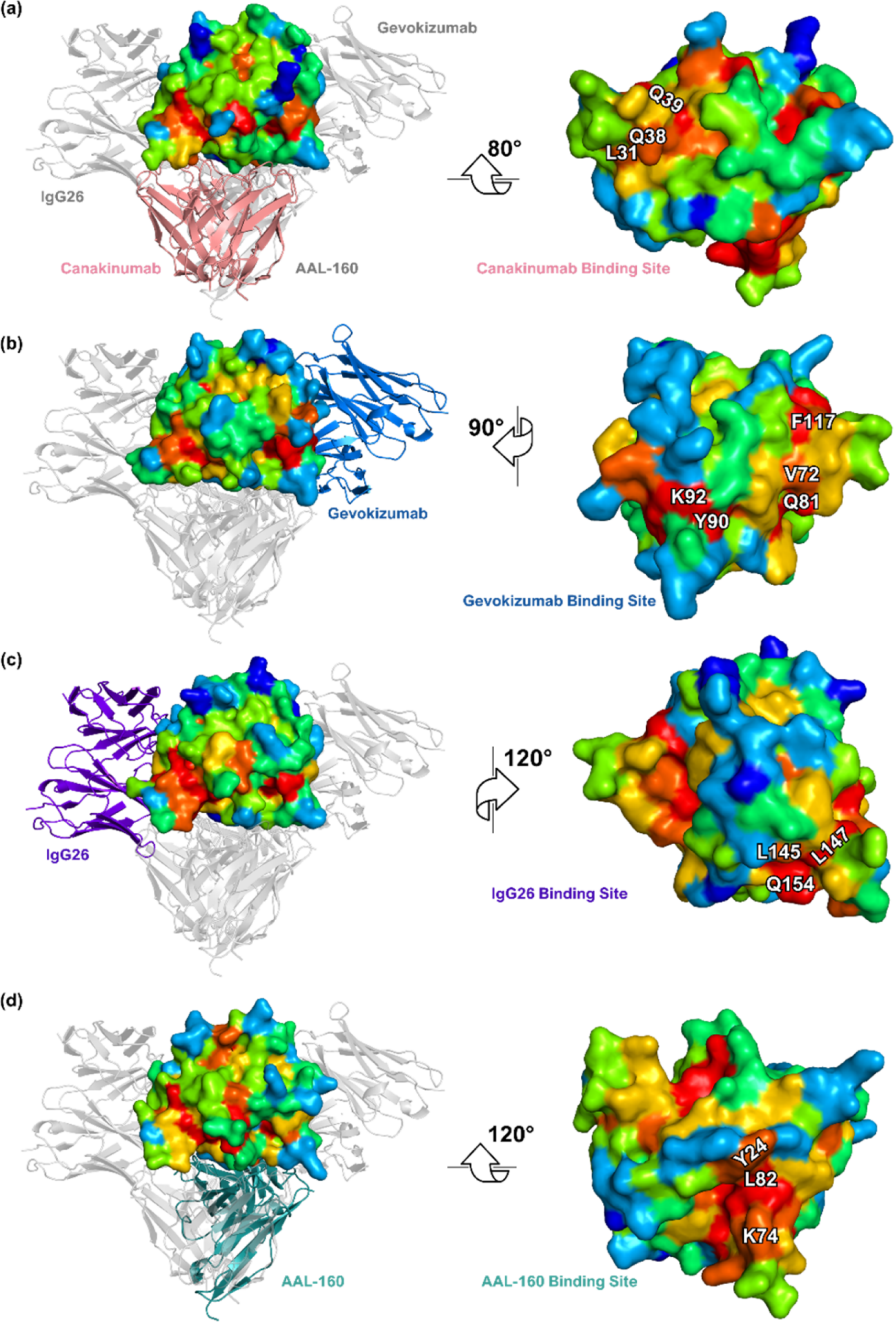
Predicted potential epitope sites in IL-1β antigens using ESSA. A rainbow coloring scheme was used, where red and orange represent the most essential residues, while dark blue represents the least essential residues. Only the bound antibody was shown in its original color, as given in Figure 1, whereas the other antibodies were demonstrated in gray cartoons. On the right panels, the antigen was rotated to show the corresponding antibody binding sites clearly. The interacting residues that were detected by ESSA were also labeled on the antigen structure.

ESSA detected between 38 and 43 (25% and 29%) amino acids as essential in four IL-1β conformations (the red- and orange-colored residues in Figure 3). Notably, ESSA successfully identified all four antibody binding sites across these conformations. Residue-level detailed analysis showed that ESSA detected at least three residues (max 5) on each corresponding epitope (Figure 3). In addition, ESSA detected a minimum of one (up to five) residue in other epitopes. We also noticed that ESSA identified the residues that are often adjacent to epitopes as essential. Especially, the Gevokizumab-removed IL-1β (4G6M) gave the best result with the detection of five interacting residues and six epitope-neighboring residues, which is the highest number of residues among all Ags studied (Figure 3b). Moreover, ESSA-detected residues in IL-1β overlap with some residues at the known binding sites of other proteins and small molecules (Figure 4). ESSA pinpointed critical residues in both site A and site B at the IL-1β:IL-1R interface, as well as the IL-1R accessory protein binding site.^41,42^

**Figure 4:**
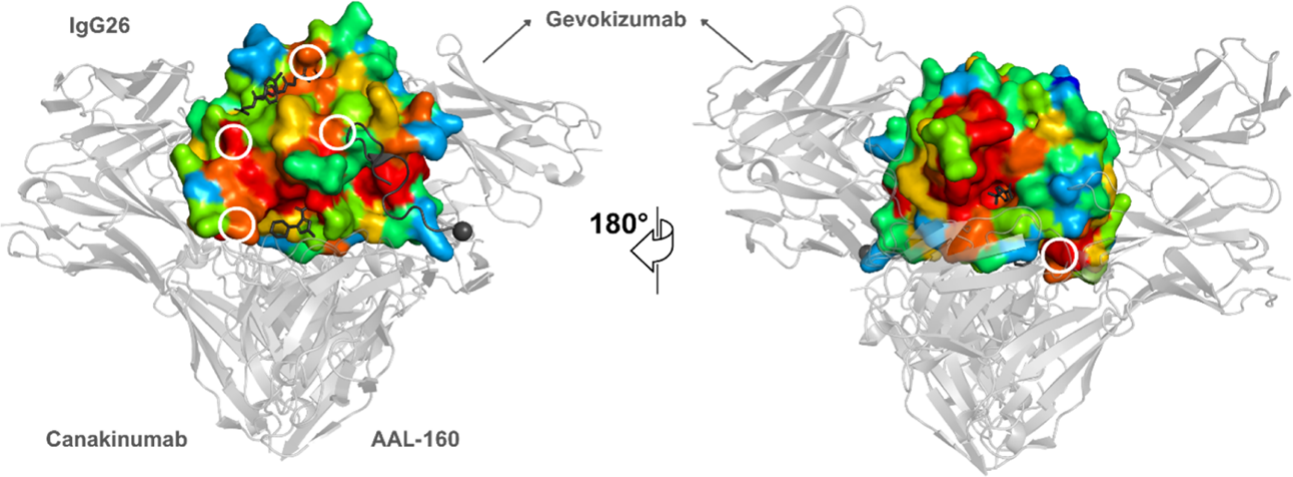
A comparison of known receptor and ligand binding sites in IL-1β and ESSA results. Each ligand/peptide was shown with black sticks. White circles represent receptor binding sites detected by ESSA. On the left, from top to bottom, molecules are SX2 (6Y8M*), macrocyclic peptide (8RYK^43^), and K3Y (5R8F^44^), respectively. On the right, the ligand is S7J (5R8D^44^), and white circles are mapped based on the receptor-bound IL-1β structure (1ITB^42^). (*not a published crystal structure).

We thought that the usage of ESSA-detected residues might be helpful in performing local (forced/guided) docking between Ag and Ab, which requires paratope information. Therefore, we further investigated four anti-IL-1β antibodies in terms of paratope prediction using ESSA.

### 3.2 ESSA Identifies Essential Residues in Anti-IL-1β Antibodies that Are Critical for the IL-1β Recognition

Anti-interleukin mAbs were also analyzed individually using ESSA to compare allosteric changes in those antibodies in the absence and presence of the antigen. The antigen part was removed to mimic the absence of the antigen and was named “Ag-removed”. We aim to identify essential paratope residues, particularly within the CDRs, and to understand the internal dynamics of the Ag-removed antibody forms. Overall, looking at the sum of the essential residues on both (H)­eavy and (L)­ight chains in antibodies, ESSA detected between 41 and 54 (9.5%–12.5%) residues. ESSA identified up to six residues on HCDR-3 across all four antibodies, including both Ag-interacting and noninteracting residues. A detailed analysis of these residues shows that at most, two Ag-interacting residues are on the HCDRs. Investigation of all detected amino acids in each antibody revealed that aromatic amino acids (Trp, Tyr, and Phe) are in the majority, with a ratio of ∼50%. In addition, ∼70% of the detected residues are hydrophobic. We aligned the sequences of all four antibodies, which were colored according to ESSA *z*-scores, and investigated the residues commonly detected (Figure S2). We found that 17 aa in the heavy chain and 16 aa in the light chain at the same sequence position were detected as essential in at least three mAbs. These residues are mainly located in three areas in antibodies: the center of the VH-VL interface, the bottom of the Fab fragment, and near the hinge regions. On each Ab, ESSA consistently detected Trp residue, which is the most seen first residue of the framework after the HCDR-3 region, which is important for the interaction with the light chain,^45^ and it was found to be critical for antibody tuning in two different experimental studies.^46,47^ These areas might be critical for the internal dynamics of the antibodies and their interaction with the Fc fragment and other proteins.

We ranked the mAbs according to their total number of ESSA-detected paratope residues and obtained the following: IgG26 > Canakinumab > AAL160 > Gevokizumab (Tables S6–S9). The ESSA results for the highest performing (IgG26) and lowest performing (Gevokizumab) cases are provided in Figure 5. IgG26 interacts with IL-1β mostly from the CDR-2 and CDR-3 regions of both heavy and light chains. As seen in Figure 5a, two residues (W52 and F99) on the paratope region of the antibody were detected using ESSA. In addition, seven residues on CDRs were detected, of which five are on HCDR-3 and two on HCDR-1. On the other hand, Ag-removed Gevokizumab showed the lowest performance among the selected mAbs because no interacting residues on its paratope were detected. Still, three residues on HCDR-3 and two residues on LCDRs were detected with ESSA (Figure 5b). Those three residues on HCDR-3, especially F107, were generally seen as residues at these positions.^45^ When all mAbs were considered, in addition to HCDR-3s, at least one residue on LCDR-3 was detected in each antibody except AAL160 (7Z4T).

**Figure 5:**
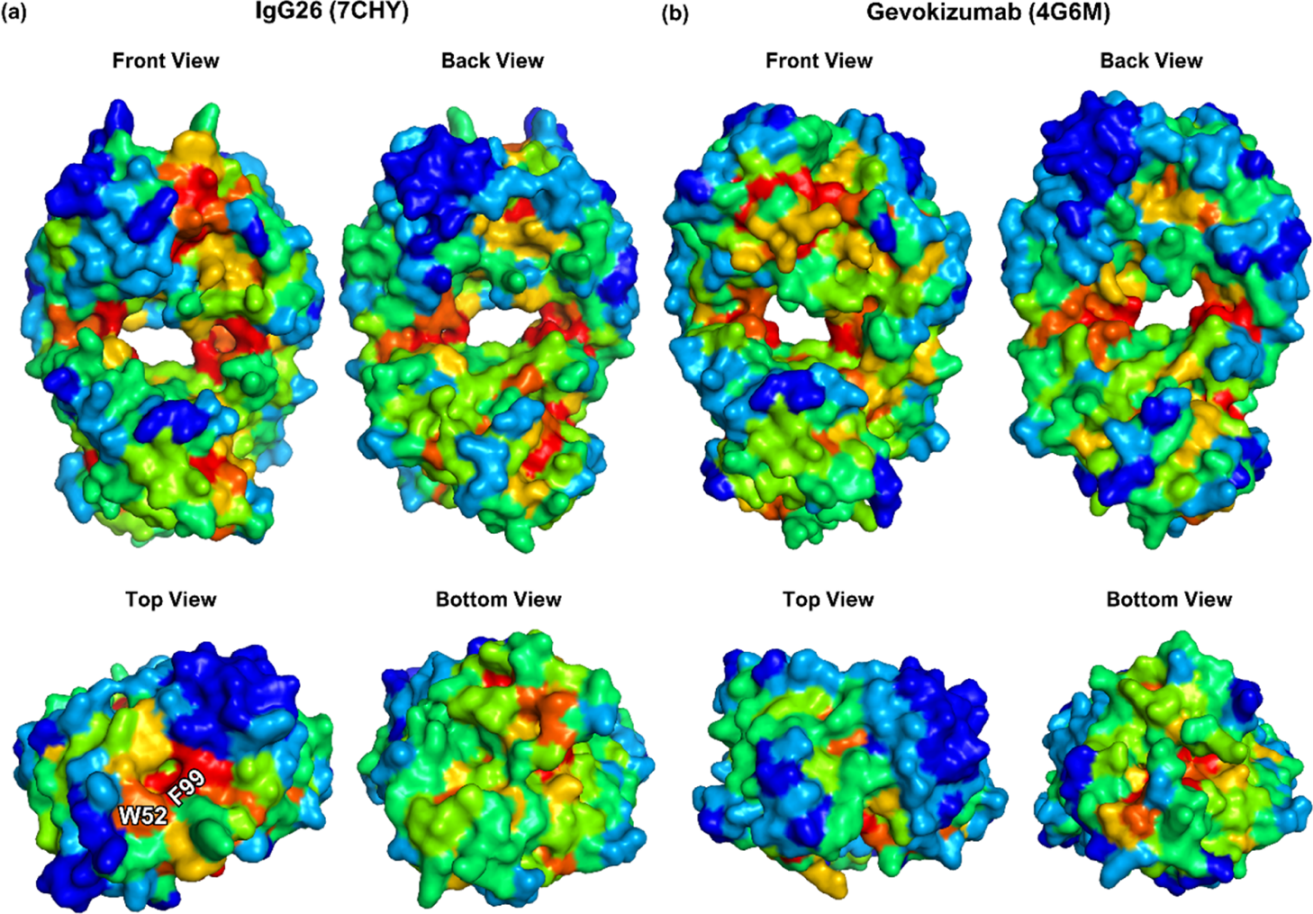
ESSA results of the IgG26 (the highest-performing, 7CHY) and Gevokizumab (the lowest-performing, 4G6M) antibodies. (a) Representation of the ESSA result for the IL-1β-removed IgG26 structure from all sides. The top view shows the paratope region of this antibody, and two detected interacting residues are labeled in the structure. (b) Representation of the ESSA result for the IL-1β-removed Gevokizumab structure from all sides. No interacting residue was detected on the paratope region by ESSA. Structures are colored using a rainbow color code based on ESSA *z*-scores. Here, the red and orange represent the most essential residues. The left side of each structure in the front view always shows the heavy chain.

#### 3.2.1 Unbiased Conformational Sampling of Gevokizumab Using ClustENMD Implies the Importance of the Antigen-Binding-Related Induced-Fit

Here, conformational changes in the Gevokizumab were investigated to see how these changes might affect ESSA results and the success of predicting Ag-interacting residues. In this respect, 300 different conformations of Gevokizumab were created using ClustENMD and ordered by decreasing RMSD values relative to the initial structure. Three frames with high RMSD with respect to the initial structure were selected and subjected to ESSA: 299th (6.98 Å), 219th (5.94 Å), and 136th (5.55 Å). The ESSA result of frame 136 yielded the highest total number of essential residues (42) (Figure S5), whereas it was 20–23 for the other frames (Figures S3, S4). If we focus on the CDRs and Ag-interacting residues, ESSA detected Phe107 and Asp109 on HCDR-3, Trp96 on LCDR-3, and no Ag-interacting residues in frame 136 (Table S10). It has been found that the length of the HCDR-3 loops affects the Ag-binding residues and loop conformations. Longer HCDR-3 loops impact the CDR surface shape and affect Ag binding of other loops, which is important for mAb design.^12^ Interestingly, in another study, it was stated that CDR-3 loops mostly preserve their conformations after binding to Ag.^48^ Under the light of this information, we checked if there is any relation between conformational changes in variable parts of the antibody and the drop-in success rate in finding Ag-interacting residues using ESSA. We compared three conformers from ClustENMD with the initial conformation and noticed a dominant motion involving a counter-rotation of the VH-VL (upper) and CH-CL (lower) parts of the antibody (Figure S6). These findings show that the antibody is highly flexible and CDRs can have different conformations in the apo-form (antigen-removed), implying that the presence of the antigen facilitates the formation of the exact conformation of CDRs via the induced-fit mechanism.

### 3.3 Essentiality in Residues Shifts toward the Paratope and Epitope Interface after the Antibody–Antigen Complex Formation in IL-1β

We were also curious about the changes in the potential binding sites and overall dynamics of the Ag and Ab parts after complex formation. Therefore, we conducted an ESSA analysis for the Ab/Ag complex structures and investigated the results in detail.

We compared the apo- (Ab-removed) and bound-form results of the IL-1β structures and detected significant changes in the *z*-score values of 36 to 50 (24%–33.3%) residues. The number of total essential residues detected decreases in each antigen structure upon binding. Although the total number of essential residues decreases upon binding, the number of Ab-interacting residues increases in all IL-1β structures except the IgG26-bound form (Figure S7). Two types of *z*-score changes were observed in the detected residues: “negative change”, where the *z*-score value of a residue is decreased with complex formation, and “positive change”, where the *z*-score is increased. With the complex formation, most of the residues that are not near the binding residues lost their significance, resulting in a negative change. On the other hand, we observe a positive change generally at and near the binding residues, even revealing new significant residues with increased *z*-score values. The best example of the positive effect on binding residues is the result of Gevokizumab-bound IL-1β (4G6M). In the Gevokizumab-removed form, ESSA detected five Ab-binding residues as essential. In the case of complex form, six new binding residues were detected (Table S12). Interestingly, five previously detected binding residues lost their significance, and their *z*-score values decreased significantly. In most antigens (except IgG26-bound IL-1β), at least two new binding residues were detected, while some previously identified residues lost significance (Tables S11–S14). In the case of IgG26-bound IL-1β, no new binding residue was detected, and *z*-scores of the previously detected three binding residues also decreased significantly (Table S13).

Following this, we analyzed the antibodies in their apo (Ag-removed) and bound states to understand how complex formation affects their essential residues. Overall, we observed significant changes in the *z*-score values of 21 to 30 residues (9.6%–13.7%) in the H-chain and 16 to 23 (7.5%–10.8%) residues in the L-chain. Just like the antigens, we also observed positive and negative changes in the *z*-scores of antibodies. These positive changes are observed mostly in the Ag-binding residues and their neighbors. In each apo antibody, we detected at least two nonbinding CDR residues using ESSA. In the bound form, we see that these nonbinding CDR residues lost their significance, and their *z*-score values decreased with a few exceptions. We see these exceptions in the Gevokizumab-bound IL-1β (4G6M) and AAL160-bound IL-1β (7Z4T). In both antibodies, *z*-scores of two new residues on CDR regions have increased to the essential range (6–7). In 7Z4T, Arg59 (HCDR-2) and Trp102 (HCDR-3), and in 4G6M, Trp106 (HCDR-3) and Ser30 (LCDR-1) were detected as essential. These newly detected residues are close to (or neighbor to) paratope residues. We mostly see the positive change in the Ag-binding residues, and when we focus on them, we see that at least three new binding residues are detected as essential in the paratope region. Normally, in the Ag-removed form, ESSA could not detect any Ag-binding residue on the Gevokizumab (4G6M). Still, in the complex form, we detect 11 binding residues as essential, which is the antibody with the most newly detected binding residues (Figure 6). Just like the results of the Ab-bound antigens, in the complex form, the residues other than paratope regions mostly lost their significance, and their *z*-score value decreased significantly (the negative change). Ag-removed IgG26 (7CHY) was the best resulting antibody among the others for detecting paratope regions using ESSA. When we focus on its complex form results, we see that only three new binding residues were detected, and one of the two detected binding residues lost its significance, along with residues in the CDR regions. If we compare the antibodies according to the number of newly detected binding residues upon complex formation, Gevokizumab has the highest number (11) (Table S16), followed by Canakinumab (6) (Table S15), then AAL160 (4) (Table S18), and finally IgG26 (3) (Table S17) (Figure S8). Overall, we can say that with the complex formation, detected residues shift toward paratope regions, and the number of detected Ag-binding residues increases.

**Figure 6:**
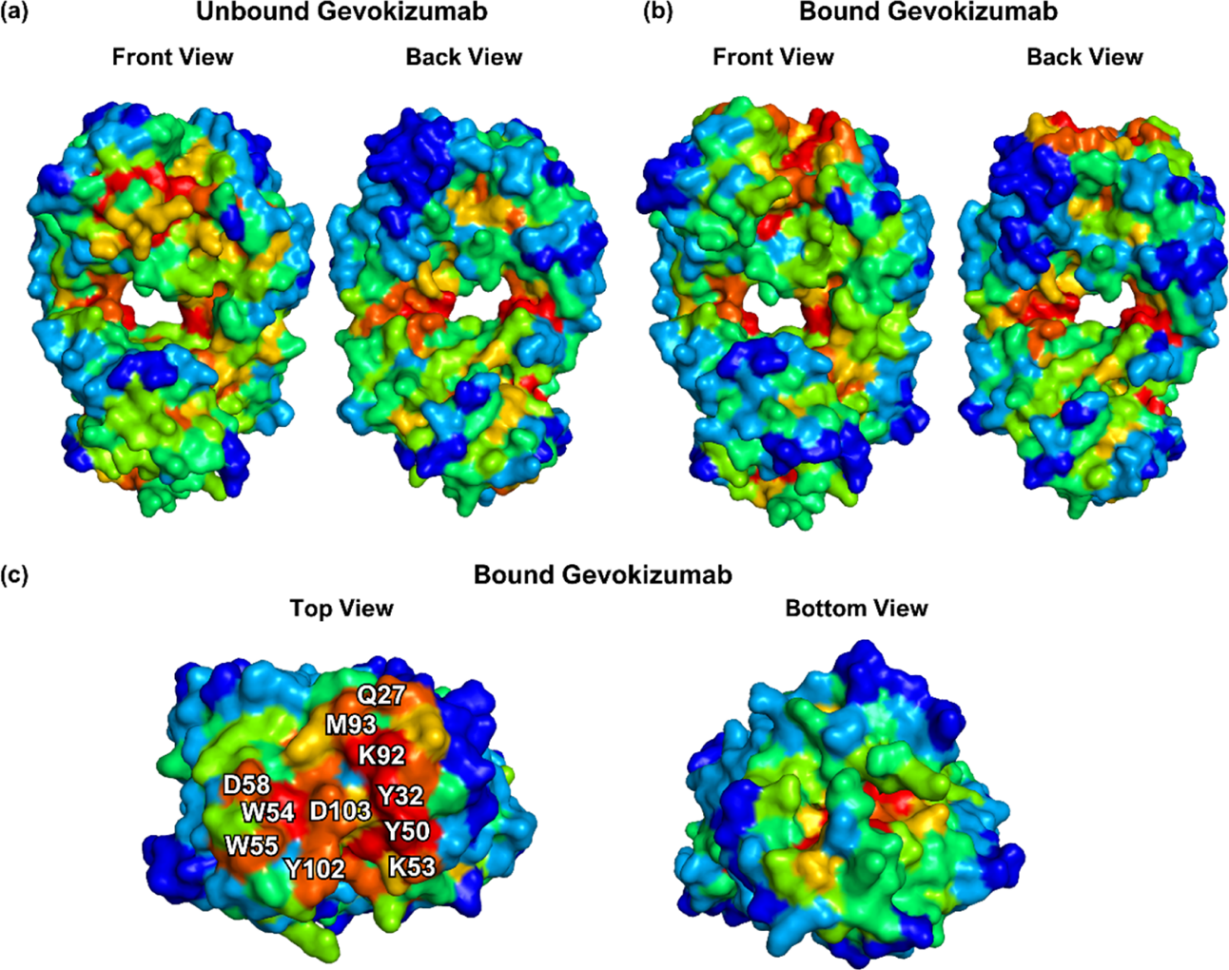
ESSA results for the Gevokizumab-bound IL-1β structure in comparison with the unbound state. (a) Unbound Gevokizumab and (b) Gevokizumab part of the Gevokizumab:IL-1β complex structure from different sides. (c) The top view shows the paratope region of Gevokizumab, whereas the bottom view shows the Fc binding region. The labeled residues are newly ESSA-detected interacting residues distinct from the unbound state. Structures are colored using a rainbow color code based on ESSA *z*-scores. Here, the red and orange represent the most essential residues. The left side of each structure in the front view always shows the heavy chain.

### 3.4 ESSA-Detected Essential Residues Yield Better Docking Poses of Antibody:IL-1β Pairs

We performed ESSA on each IL-1β, its mAbs, and their complex structures and detected essential residues on their paratope/epitope regions or residues specifically on CDR regions. To control the effect of essential residue information obtained from ESSA on the prediction of the correct pose of the Ab/Ag complex structure, we proceeded with protein–protein, in our case Ab–Ag, docking studies. Ags and mAbs were uploaded as receptors and ligands, respectively, in the ClusPro protein–protein docking tool. Docking calculations were performed under three categories, Blind, HCDR_ESSA, and Ag_ESSA dockings, of which the last two are ESSA-guided dockings. For the HCDR_ESSA docking, forced (local) docking was performed using the detected essential residues on HCDRs (mostly HCDR-3) from ESSA, whereas the detected essential residues on epitope regions were used for the Ag_ESSA docking. Docking cluster center models ranked based on their energy values, which were calculated using the “Balanced” scoring scheme in ClusPro, were used to compare three docking categories performed in this study.

The ranking and scores of the Ab/Ag docking results (Table 2) show that Canakinumab:IL-1β (4G6J) gave the best result (1st ranking) among the four Ab/Ag crystal complexes in all docking categories. In the HCDR_ESSA docking for the Canakinumab-bound case, we selected four residues on the HCDR-3 that were detected as essential using ESSA. For the Ag_ESSA docking, three residues on the epitope region obtained from ESSA were used, as shown in Table S21. The ClusPro docking analysis yielded 17 poses for the HCDR_ESSA, 30 for both the Ag_ESSA and Blind docking. Even though we separately performed the docking calculations, the best poses for the HCDR_ESSA and Ag_ESSA are the same (Figure 7b,c). However, the Ag_ESSA docking pose has the best score (−633.7) among these three. Furthermore, Ab–Ag interactions in these poses were determined using PDBsum and compared with those in the original conformation. Due to conformational changes, slight differences in interactions and increased RMSD values relative to the original structure were observed, but most of the original interactions (especially between the H-chain and Ag) were maintained. Formed hydrogen bonds between the L-chain and Ag were preserved, but some of the nonbonded contacts were missing (Tables S22 and S23). Interestingly, RMSD values are higher in ESSA-guided dockings than in the Blind_docking results.

**Table 2:** ClusPro Docking Scores and Ranking Results for the Studied IL-1β-Targeting Antibodies

	Blind		HCDR_ESSA		Ag_ESSA	
structures	rank^a^	score	rank	score	rank	score
4G6J (canakinumab:IL-1β)	1/30	(−573.8)	1/17	(−583.4)	1/30	(−633.7)
4G6M (gevokizumab:IL-1β)	13/19	(−462.0)	13/18	(−472.1)	13/17	(−477.5)
7CHY (IgG26:IL-1β)	2/20	(−722.4)	3/18	(−821.7)	11/21	(−655.9)
7Z4T (AAL160:IL-1β)	2/26	(−561.0)	2/26	(−579.2)	1/24	(−674.4)

a In ranking values, the numerator represents the rank of the cluster that is most similar to the original crystal complex structure, and the denominator represents the total number of obtained clusters from ClusPro.

**Figure 7:**
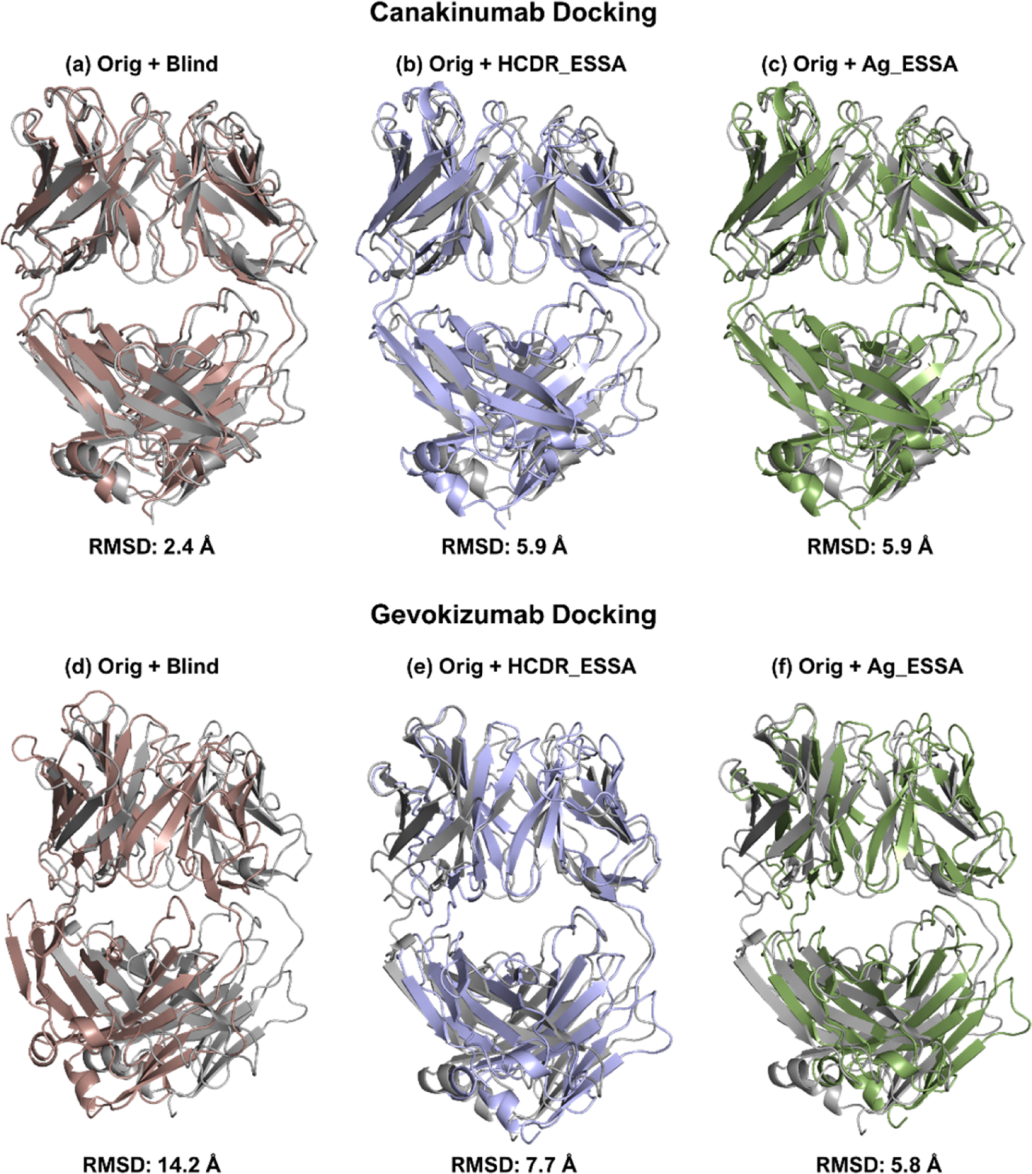
Comparison of the best docking poses for Canakinumab (the best-performing) and Gevokizumab (the least-performing) binding to IL-1β. (a) Canakinumab original vs blind docking pose, (b) Canakinumab original vs HCDR_ESSA docking pose, (c) Canakinumab original vs Ag_ESSA docking pose, (d) Gevokizumab original vs blind docking pose, (e) Gevokizumab original vs HCDR_ESSA docking pose, and (f) Gevokizumab original vs Ag_ESSA docking pose. The original crystal structure is written as “Orig” in the figure. RMSD values are calculated between the original structure and each docking pose. The original structure is shown as light gray, whereas the Blind docking pose is light rose, HCDR_ESSA slate, and Ag_ESSA dark green.

Gevokizumab:IL-1β (4G6M) docking results are the least successful among the others in terms of both ranking and scores. In the HCDR_ESSA docking, we selected three residues on the HCDR-3 detected as essential using ESSA, and for the Ag_ESSA docking, five residues on the epitope detected as essential using ESSA were selected for the forced docking. In total, we obtained 17–19 poses for each docking for 4G6M. The best pose for each docking was detected at rank 13, and the best-obtained score (−477.5) was obtained from Ag_ESSA docking (Table 2). As shown in Figure 7, the Blind_docking pose provided the most different structure from the corresponding original structure (RMSD = 14.2 Å) compared to ESSA-guided docking poses; however, most of the original H-bonds were maintained. On the other hand, the ESSA-guided dockings yielded lower RMSD values (≤7.7 Å) and a few preserved H-bonds (Tables S24–S26).

Finally, to evaluate the specificity and compatibility of Ab/Ag interactions, we performed cross-docking calculations by swapping antibodies between distinct epitopes. We docked Gevokizumab onto the epitope of Canakinumab in IL-1β, and similarly, Canakinumab was docked onto the epitope of Gevokizumab (Figure 8). We used the critical residue information for each possible epitope, obtained solely from the ESSA results, to mimic the situation in which the native epitope residues are not known; therefore, docking was performed without any information on the complex crystal structure. The swapped-docking results were sorted by the number of interacting residues in HCDRs within 5 Å of ESSA-detected epitopes. The docking pose with the highest number of interacting residues was selected as the best pose and provided as a green cartoon in Figure 8 for each swapped docking. Swapping the antibodies between their native epitopes resulted in significantly poorer docking scores and less favorable binding poses, highlighting the importance of epitope-specific interactions. Docking of Canakinumab to its native epitope yielded the highest binding score and correct pose (Canakinumab_native_ = −633.7), whereas docking to the non-native epitope produced a lower binding score (Canakinumab_non‑native_ = −493.2). The same trend was observed for Gevokizumab, which favored the native epitope over the non-native epitope (Gevokizumab_native_ = −477.5 and Gevokizumab_non‑native_ = −455.6). Interestingly, none of the swapped Gevokizumab poses was near the non-native epitope, which is Canakinumab’s native epitope. In Supplementary Figure S10, all poses obtained from Gevokizumab-swapped docking were provided from two different angles. However, there were two binding poses that slightly overlapped with Canakinumab, but the antibody is interacting with the antigen via its hinge region, not the HCDRs or LCDRs. Our swapped docking results show a similar pattern, reinforcing the specificity of antigen–antibody recognition, driven by critical contact residues at each epitope. As a result, ESSA can be used as a powerful tool to assist docking methods in the computational determination of Ab/Ag complex structures by enabling the accurate and rapid identification of the critical residues involved in Ab/Ag binding.

**Figure 8:**
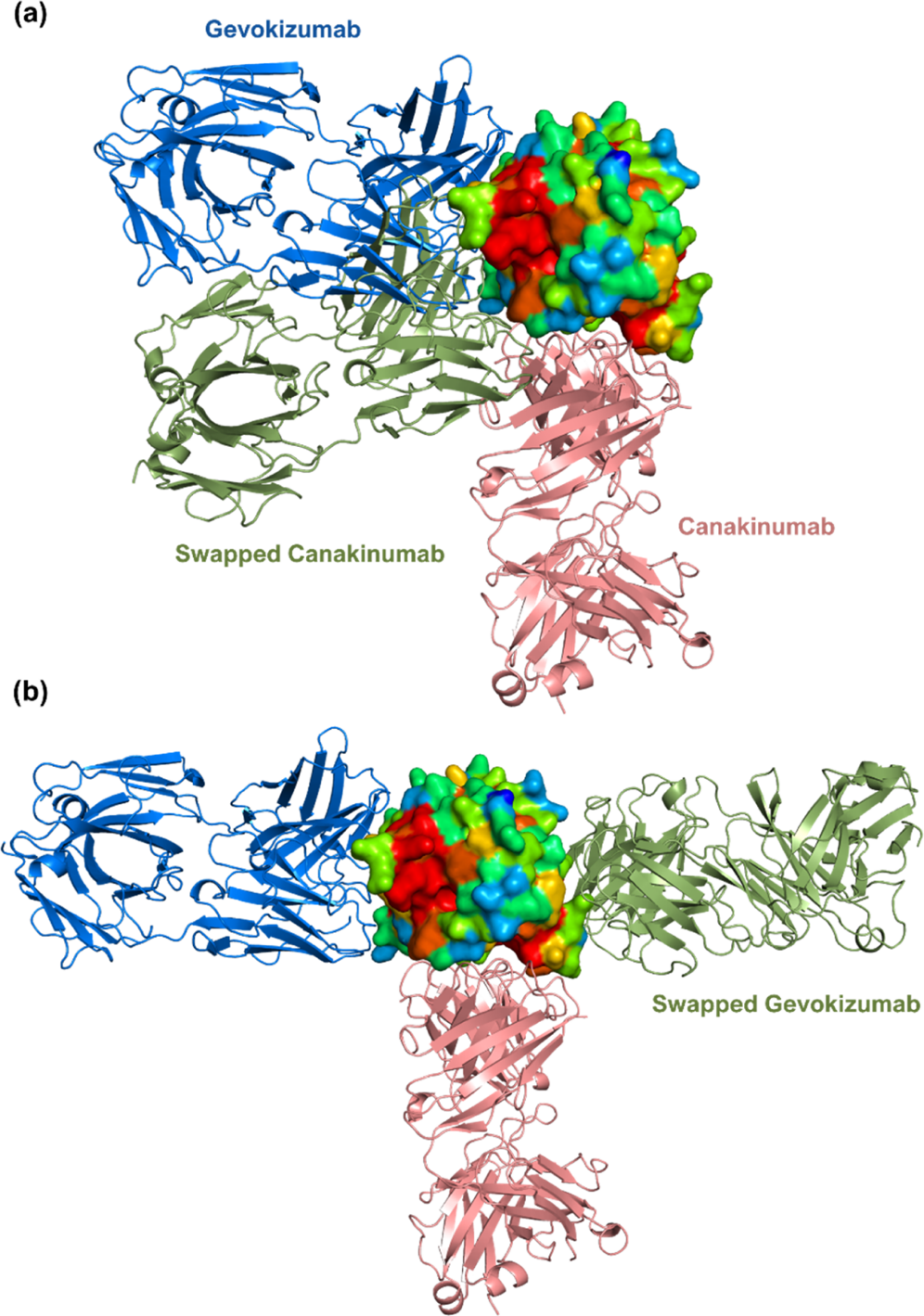
Best docking poses from swapped docking for Canakinumab and Gevokizumab. The best poses for (a) the swapped Canakinumab and (b) the swapped Gevokizumab are shown in green. The antigen is colored using a rainbow color code based on its ESSA *z*-scores. Here, the red and orange represent the most essential residues in the antigen.

The Blind and ESSA-guided dockings were also performed for other antibodies studied, IgG26 (7CHY) and AAL160 (7Z4T) (Table S21). Considering their ranking and docking scores, the ESSA-guided dockings (HCDR_ESSA and Ag_ESSA) yielded better results than blind docking (Table 2). The best docking poses are shown in Figure S9. Although the Ag_ESSA docking poses for both cases yielded conformations that differed from the corresponding original structures, critical interactions between Ag and Ab were also maintained in these poses (Tables S27–S31).

To sum up, detecting essential residues using ESSA on both the antigen and the antibody helps improve Ab/Ag docking and yields better antibody:IL-1β complex poses. ESSA might provide key information for the local docking and facilitate the docking procedure. However, we think that the RMSD alone is not a measure of correctness. Guided dockings might yield more accurate binding poses; however, they do not guarantee that they capture all critical interactions observed in the crystal structure. Docking poses represent a single static conformation and lack dynamic information. RMSD comparisons based on a single structure are insufficient for reliable conclusions. To address this limitation, we performed all-atom conventional MD simulations for the Blind and Ag_ESSA docking poses of Gevokizumab:IL-1β (4G6M), due to being less successful at docking pose prediction than others.

### 3.5 MD Simulations Revealed More Stable Interactions in the ESSA-Guided Docking Pose of Gevokizumab:IL-1β

The RMSD between the original structure and the Ag_ESSA docking pose showed that ESSA-guided docking yields better results. To investigate the stability of the docking poses for the Ab/Ag complex, first we performed three replicate (300 ns each) all-atom MD simulations on Blind and Ag_ESSA-guided docking poses and collected 15,000 frames/run for each structure. Then, we calculated the corresponding RMSD profiles, which show the stability of each complex, and the number of hydrogen bonds formed between the Ab and Ag. In the initial MD run01, we observed that the Ag_ESSA pose is more stabilized than the Blind structure, and when we compare the highest RMSD values for both structures, Ag_ESSA has a lower (3.75 Å) value than the Blind pose (4.91 Å). Figure S11 shows the frame-by-frame RMSD comparison plot of the three runs for each pose from the initial docking pose. The average of the RMSD values in three runs of each structures proved that Ag_ESSA is indeed more stabilized (RMSD_AverageAll_ = 2.66 Å) than the Blind structure (RMSD_AverageAll_ = 3.04 Å). Additionally, we calculated the RMSD for each complex relative to the original crystal structure and reported it as a line plot (Figure S12). In the initial run, the Ag_ESSA RMSD profile has a very narrow range (RMSD_min_ = 1.26 Å, RMSD_average_ = 1.9 Å, RMSD_max_ = 3.02 Å), in which RMSD ≤2 Å in more than 10,000 frames, yielding conformations more similar to the crystal complex. On the other hand, the Blind docking frames showed higher RMSD values (RMSD_min_ = 1.7 Å, RMSD_average_ = 3.04 Å, RMSD_max_ = 5.92 Å), giving a wider range and yielding only 188 frames with RMSD <2 Å. RMSD comparison of the three replica runs of the Ag_ESSA and Blind poses with a crystal structure shows a similar result in general. The RMSD_average_ of 45,000 frames in Ag_ESSA is 2.35 Å, and for the Blind, this value is 2.88 Å. The difference between the RMSD_min_ values is small; however, we observe a significant difference in the number of hydrogen bonds formed between the antibody and the antigen.

In the original crystal structure (4G6M), there are 15 hydrogen bonds formed between Ab and Ag. All these hydrogen bonds are shown in Figure 9(a). In each frame, we specifically focus on the hydrogen bonds that originally formed. For this aim, we calculated the H-bonds in each frame of the trajectories and determined the percentage of frames in which the original H-bonds were observed. In the Ag_ESSA, every single frame includes at least one of the original H-bonds observed in the crystal structure (except the third run). On the other hand, some frames from the Blind docking pose MD simulations lack any original H-bond (except the third run). Then, we focused on the last 100 ns of the trajectories and identified hydrogen bonds observed in at least 50% of the 5000 frames in each structure. This filtering yielded eight original H-bonds in Ag_ESSA (Figure 9b) and only one in Blind (Figure 9c) in the first MD run. All the observed H-bonds using this threshold are shown in Tables S19 and S20 for Ag_ESSA and Blind, respectively. Ag/E83-OE2–AbL/Y32–OH is the most seen H-bond for Ag_ESSA, with 4969 (99.38%) frames, and for the Blind, it is Ag/K97-NZ–AbH/D56-OD2 with 3533 (70.66%) frames. In the second run with this filtering, we observed seven original H-bonds in Ag_ESSA, but the only bond observed in the first MD of the Blind docking pose could not pass the filtering. Again, the Ag/E83-OE2–AbL/Y32–OH is the most seen H-bond for Ag_ESSA with 4987 (99.74%) frames. In the third run, through this filtering, we observed three H-bonds in Blind and only one in Ag_ESSA. The most seen H-bond in the third run for both structures is Ag/K97-NZ–AbH/D56-OD2 with 3723 frames (74.46%) in Ag_ESSA and 3936 frames (78.72%) in Blind. When the filtering threshold increased to 80%, we observed that the Ag_ESSA still has five and three original H-bonds in two replica runs, respectively, and none in the third replica, whereas the Blind has none in all three replica runs. The persistent H-bonds observed in Ag_ESSA simulations include residues from CDRs (one in CDR1, one in CDR2, and three in CDR3). On the contrary, the Blind docking pose revealed H-bonds with the antigen only with two residues from CDR2, and none from CDR1 and CDR3. With such a high filtering threshold, these findings indicate that two of the Ag_ESSA simulations spanned a similar state where higher occupancy values were observed for some H-bonds observed in the crystal structure, whereas the third run showed a transient trend with lower occupancy values. On the other hand, the Blind docking pose could not produce persistent H-bonds in all three replicates. As a result, all MD simulation findings showed that ESSA-guided docking poses are closer to the crystal structure in terms of both interactions and structural similarity.

**Figure 9:**
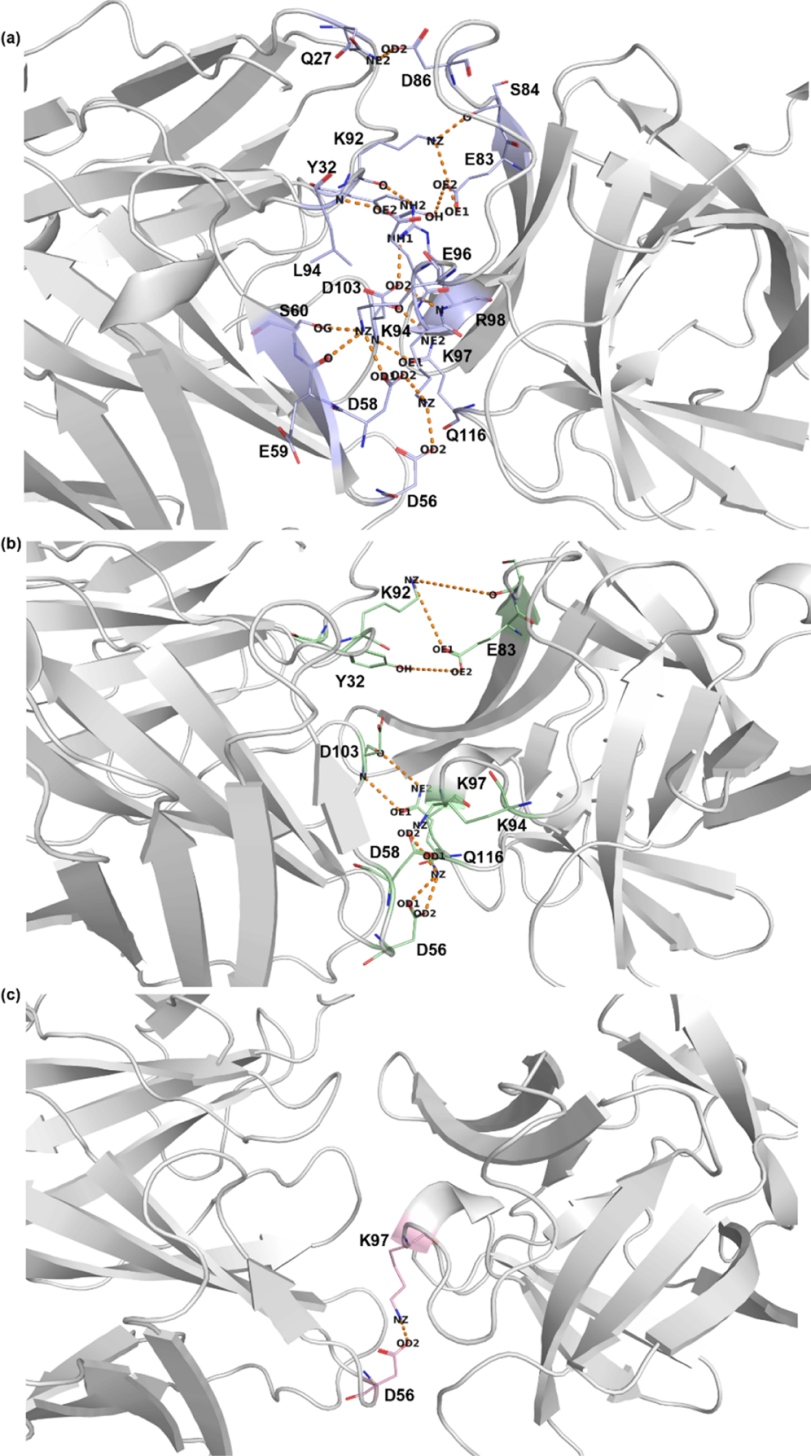
Formed hydrogen bonds in the Gevokizumab-bound IL-1β. (a) Originally formed 15 H-bonds in Gevokizumab-bound IL-1β (PDB ID: 4G6M ^3^). (b) The most seen eight originally formed H-bonds in the Run01 Ag_ESSA pose. (c) The most seen H-bond in the Run01 Blind pose. Residues that form H-bonds were colored purple, green, and pink in each structure, respectively. H-bonds were represented with orange dashed lines. In each panel, the right side is IL-1β, and the left side is Ab.

### 3.6 EPIGUIDE, an Epitope and Binding Pose Prediction Strategy, Revealed Successful Results

When the knowledge-based workflow (Figure 2A) was provided with the true epitope residues as constraints, which overlap with the ESSA results, ESSA-guided dockings successfully predicted the correct Ab/Ag poses and preserved some of the important interactions between Ab and Ag. However, this user-defined approach requires prior knowledge of the epitope, which is typically not available in prospective studies. This limitation raised a key question: could ESSA itself be used to predict epitope footprint, thereby eliminating the need for external input? To address this question, we developed EPIGUIDE, a complete blind prediction approach (Figure 2B), and extended our validation to a diverse set of 11 antigen–antibody complexes spanning different protein families and binding modes (Table 1).

First, we compared our epitope predictions obtained from ESSA to two different methods, namely DiscoTope-3.0 ^49^ and SEPPA 3.0.^50^ For a fair comparison, the same 3D structures of antigens in the data set were used as input in all calculations. Table 3 summarizes the results obtained from ESSA, DiscoTope-3.0, and SEPPA 3.0 tools. We determined only the known epitopes for each studied antigen by extracting all related complex crystal structures in the Protein Data Bank corresponding to each antigen studied. Then, for other proteins in these complex structures, their interacting residues within 4 Å of the protein with the antigen were determined. Therefore, all known linear and discontinuous epitope residues were collected for each antigen. Next, we clustered these known epitope residues using different distance threshold values ranging from 10 Å to 16 Å. We applied the elbow method to determine which distance threshold value is good enough. The elbow method results are provided in Supplementary Figure S13. According to the results, we selected a distance threshold value of 14 Å and obtained 62 clusters in total using the distance threshold value (Table 3). In the clustering, a minimum of three clusters for each antigen were reached. Then, for each prediction tool, we calculated the total number of successfully predicted known epitope clusters where at least one high-scored residue is in the cluster. In total, DiscoTope-3.0 and SEPPA 3.0 predicted 52 out of 62 epitope clusters, whereas ESSA with a threshold value of 6 (named ESSA6) was able to predict 47 known epitope clusters. Additionally, we decreased this threshold value to 5 (named ESSA5) and obtained an increment to a value of 52, which is the same number of predictions compared to that of other tools. However, having a lower threshold value in any epitope prediction tool would yield more residues, therefore offering more target epitope regions to be used in docking calculations, increasing the docking cost. We deliberately chose the more challenging ESSA model that produces fewer results and, next, conducted our docking calculations using the determined epitope regions by ESSA6.

**Table 3:** Epitope Prediction Results for ESSA, DiscoTope-3.0, and SEPPA 3.0^a^

PDB ID	total number of known epitope clusters	ESSA6	ESSA5	DiscoTope-3.0	SEPPA 3.0
1OAK	8	5	6	**8**	3
3S36	5	**5**	**5**	2	**5**
3WD5	8	5	6	**7**	6
4DGI	3	**3**	**3**	**3**	**3**
4G3Y	7	5	**6**	**6**	**6**
4G6J	10	7	8	8	**9**
5GGT	3	**3**	**3**	**3**	**3**
5Y9J	6	5	**6**	5	5
7C88	4	3	3	3	**4**
7E9B	5	3	3	4	**5**
8Y31	3	**3**	**3**	**3**	**3**
total	62	47	52	52	52
average	5.64	4.27	4.73	4.73	4.73

a (The best prediction for each antigen is written in bold).

We evaluated the docking performance of EPIGUIDE (with ESSA6) on 11 protein targets using two different epitope prediction strategies (Max and Ave) as explained in the Materials and Methods section. The numbers (1, 2, and 3) in our epitope merging strategies refer to distinct predicted epitope regions identified by EPIGUIDE, with “1 + 2” indicating that we merged the first and second predicted epitope regions for docking restraints, “2 + 3” merging the second and third regions, and so forth (Table S32). We defined a successful “complete blind prediction” as one where the top-ranked model (i.e., the pose with the highest docking score) achieved an RMSD ≤10 Å from the native structure, which is a commonly used threshold for near-native predictions in CAPRI.^51^ Using this definition, the success rate was 45% (5 out of 11 cases). Among these, three predictions (4G3Y, 4G6J, and 5Y9J) were of medium quality (RMSD ≤5 Å CAPRI criteria). Notably, all are below 2.5 Å. Two models (3S36 and 5GGT) were of acceptable quality (5 Å < RMSD ≤10 Å), based on CAPRI classification criteria.

Then, we investigated all top models having the best docking score and noticed that at least one top model with RMSD ≤10 Å from the native structure exists for 7 out of 11 cases, increasing the success rate to 64%. Among these successes, four predictions were of medium quality (RMSD ≤5 Å CAPRI criteria). Notably, all are below 2.5 Å, where one of them (1OAK) is below 2.0 Å, and can be considered as a near-high-quality model (RMSD ≤1 Å CAPRI criteria). Three models were of acceptable quality (5 Å < RMSD ≤10 Å), based on CAPRI classification criteria.

Additionally, when the top three models coming from the best docking score-set were considered separately for each case, our success rate did not change (64%). While the top-ranked model for 1OAK was completely incorrect (110.88 Å) using Max_Ave–Ep1 + Ep2, the third-ranked model achieved an excellent RMSD value of 1.76 Å, making the model almost a high-quality one. Similarly, the RMSD value for the 7E9B case significantly improved from 65 Å to 1.93 Å, yielding a near-high-quality model. Moreover, expanding our consideration to the top five models increased the overall success rate to 73%. For 8Y31, a successful prediction emerged at fifth rank, with RMSD improving from 81.73 Å to 9.50 Å.

For 4 out of 11 proteins, Max and Ave epitope-determining strategies yielded different top docking poses. Interestingly, for 4G3Y, both strategies have very good docking poses, although their docking scores are different. For 5Y9J, the Max-Ep1 + Ep3 strategy yielded a very good docking pose with 2.22 Å of RMSD, and its top docking score is higher than the Ave, whereas the Ave strategy could not find a pose near the original. On the contrary, for 8Y31, Max yielded a better docking score but worse prediction, whereas the prediction using Ave is in an acceptable range (RMSD = 9.5 Å). Interestingly, for 7E9B, none of the top predictions using Max or Ave overlaps with the original pose. However, both Max—Ep1 + Ep2 and Ave—Ep1 + Ep3 third-ranked models overlap with the original pose and yield a 1.93 Å RMSD value, which is in the near-high-quality range based on CAPRI classification criteria. Therefore, the top 3 ranked models successfully predict the correct pose for the best-scoring epitopes. All the top predictions with the highest docking scores in comparison with corresponding crystal structures are shown in Figure 10.

**Figure 10:**
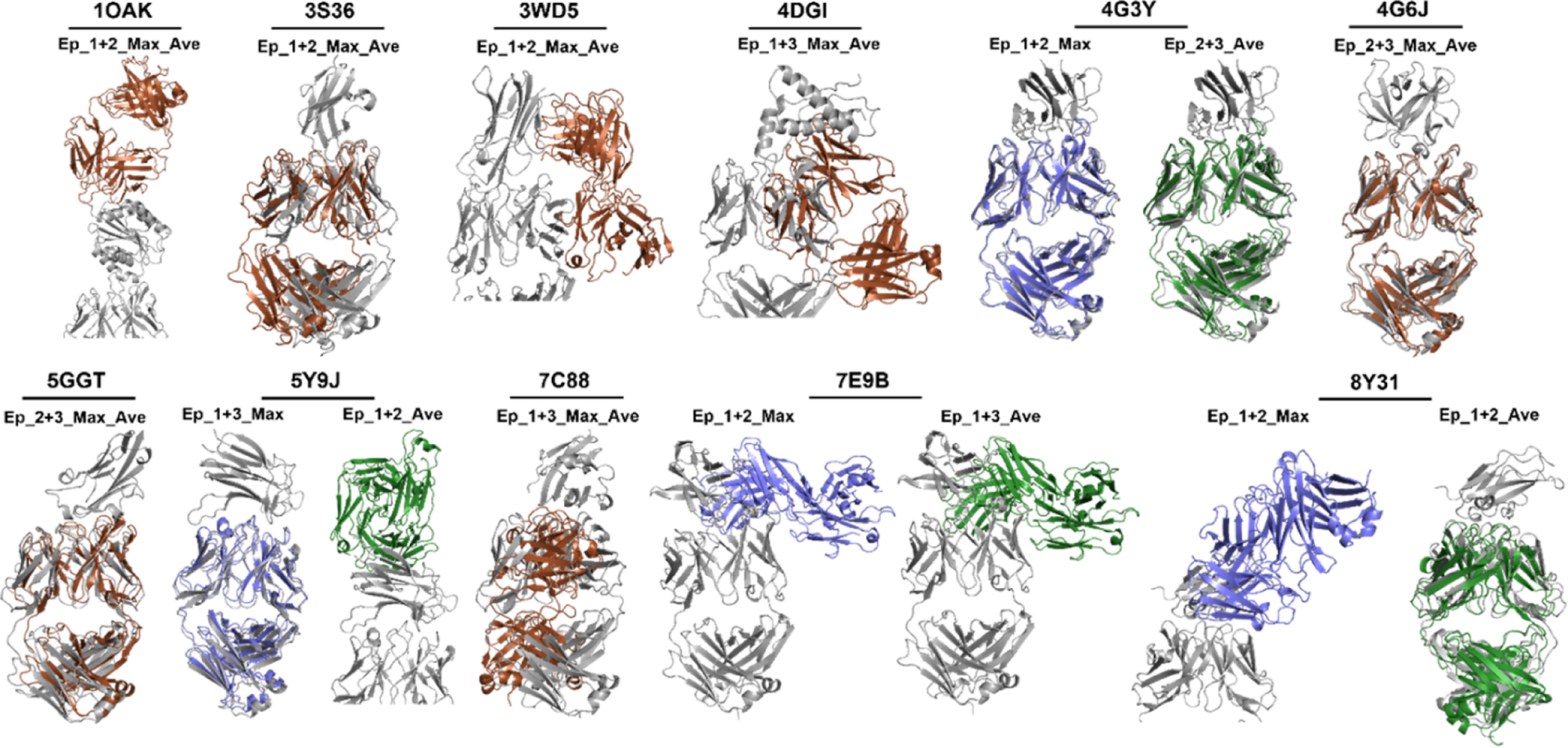
Comparison between EPIGUIDE predictions and corresponding crystal structures in the data set. The original antibody position was shown in gray.

Regarding the epitope-determining approaches (“Max” and “Ave”), they yielded the same results for seven proteins in the data set and were written as “Max_Ave” in the second column of Table 3. In most cases, the successful predictions were obtained when either the first or the second epitope was used in the calculations. This clearly shows the success of ESSA in detecting an original epitope region as the first or second rank for these cases. However, we think that different epitope prediction strategies capture different biological signals, and using multiple strategies increases the chance of successful prediction.

We also compared our EPIGUIDE results with AlphaFold3 predictions, as reported in the last column of Table 4. First, we used RMSD values for comparison. AlphaFold3 successfully predicted seven complexes (RMSD ≤10 Å), matching EPIGUIDE’s overall success count. However, a more nuanced analysis reveals important differences. For 4G6J, EPIGUIDE achieved an excellent 2.33 Å RMSD, while AlphaFold3 produced a poor prediction (53.22 Å), highlighting EPIGUIDE’s strength for this particular complex. For 8Y31, AlphaFold3 failed with 76.16 Å, whereas EPIGUIDE predicted the complex with 9.5 Å, which is within the acceptable range. For two cases, 5Y9J and 5GGT, both methods were successful; however, EPIGUIDE predictions were closer to the original structure. There are also cases (1OAK and 7E9B) where EPIGUIDE’s lower-ranked models achieved excellent accuracy even when the top-ranked model failed. In two cases (3WD5 and 4DGI), AlphaFold3 showed superior performance. 7C88 is the most difficult case where both EPIGUIDE and AlphaFold3 struggled (27.73 Å and 82.20 Å, respectively).

**Table 4:** EPIGUIDE Prediction Results^a^

PDB_ID	type and epitopes	score	first-ranked model overlap with Orig	Ab RMSD (L-RMSD Å)	ratio of the top models with RMSD ≤ 10 Å	AlphaFold vs Orig RMSD Comp. (Å)
1OAK	Max_Ave—1 + 2	–964.5	no (3rd overlaps)	110.88 (1.76)**	1/3	112.92
3S36	Max_Ave—1 + 2	–693.2	yes	8.84	2/3	2.70
3WD5	Max_Ave—1 + 3	–829.4	no	50.66	0/3	4.48
4DGI	Max_Ave—1 + 3	–698.4	no	35.21	0/3	7.90
4G3Y	Max—1 + 2	–783.3	yes	2.37	3/4	**1.70**
	Ave—2 + 3	–850.7	yes	**2.41**		
4G6J	Max_Ave—2 + 3	–576.6	yes	**2.33**	3/3	53.22
5GGT	Max_Ave—2 + 3	–750.5	yes	5.97	3/3	7.26
5Y9J	Max—1 + 3	–903.3	yes	**2.22**	3/5	5.22
	Ave—1 + 2	–841.6	no (5th overlaps)	92.8 (4.17)*		
7C88	Max_Ave—1 + 3	–695.4	yes	27.73	0/3	82.20
7E9B	Max—1 + 2	–908.1	no (3rd overlaps)	64.6 (1.93)*	0/6	9.20
	Ave—1 + 3	–963.0	no (3rd overlaps)	65.33 (1.93)*		
8Y31	Max—1 + 2	–589.4	no (5th overlaps)	81.73 (9.50)**	1/5	76.16
	Ave—1 + 2	–563.3	yes	9.50		

a The last column represents the AlphaFold3 prediction results for comparison (predictions considering only the top docking scored ones with an RMSD ≤ 2.5 Å are written in bold. “*” represents a model where it is not first-ranked, but has a good RMSD value in higher ranked model(s)).

Second, we calculated DockQ^52^ values of each heavy and light chain in each antibody studied. The original crystal complex structures were used as the reference structure in DockQ calculations. Twenty-two DockQ values for the best EPIGUIDE and AlphaFold3 predictions in the top 3 models were obtained individually and are provided in Table 5. DockQ classification was used to evaluate the quality of the predicted models: 0.00 ≤ DockQ <0.23—Incorrect; 0.23 ≤ DockQ <0.49—acceptable quality; 0.49 ≤ DockQ <0.80—medium quality; DockQ ≥0.80—high quality. Considering only the heavy and light chain predictions separately, EPIGUIDE produced 11 high-quality, 3 medium-quality, and 8 incorrect structures, while AlphaFold3 yielded 5 high-quality, 9 medium-quality, and 8 incorrect structures. EPIGUIDE and AlphaFold3 achieved very similar maximum DockQ scores (0.96 versus 0.94, respectively). Interestingly, both tools yielded very high DockQ scores for the light chains (0.96 versus 0.94) compared to those of heavy chains (0.92 versus 0.88). The average DockQ values for EPIGUIDE were consistently above those of AlphaFold3 across all categories (combined: 0.58 vs 0.51; heavy-only: 0.59 vs 0.49; light-only: 0.57 vs 0.52). Similarly, EPIGUIDE showed higher average native contact fractions (*f*_nat_) than AlphaFold3 overall (0.60 vs 0.55), as well as in heavy-only (0.63 vs 0.55) and light-only (0.58 vs 0.52) evaluations. These results suggest that EPIGUIDE can provide high-quality complex models preserving native interface contacts. However, both methods produced a comparable number of incorrect predictions.

**Table 5:** DockQ and Native Contact Fraction (*f*_nat_) Values of Heavy and Light Chains in the Top 3 Predicted Models Using EPIGUIDE and AlphaFold3^a^

		EPIGUIDE				AlphaFold			
PDB ID	native chains	DockQ	*f*_nat_	RMSD	quality DockQ	DockQ	*f*_nat_	RMSD	quality DockQ
1OAK	H	0.91	0.92	1.76	**high**	0.03	0.00	112.92	incorrect
	L	0.96	1.00	1.76	**high**	0.03	0.00	112.92	incorrect
3S36	H	0.84	0.93	8.84	**high**	0.72	0.93	2.70	**high**
	L	0.79	0.95	8.84	medium	0.80	0.75	2.70	medium
3WD5	H	0.02	0.00	50.66	incorrect	0.88	0.90	4.48	**high**
	L	0.01	0.00	50.66	incorrect	0.79	0.71	4.48	medium
4DGI	H	0.16	0.15	35.21	incorrect	0.63	0.96	7.90	medium
	L	0.16	0.11	35.21	incorrect	0.73	0.95	7.90	medium
4G3Y	H	0.92	0.95	2.41	**high**	0.86	0.81	1.70	**high**
	L	0.87	0.86	2.41	**high**	0.94	0.93	1.70	**high**
4G6J	H	0.91	0.98	2.33	**high**	0.06	0.05	53.22	incorrect
	L	0.84	0.80	2.33	**high**	0.06	0.05	53.22	incorrect
5GGT	H	0.77	0.92	5.97	medium	0.57	0.64	7.26	medium
	L	0.73	0.73	5.97	medium	0.76	0.64	7.26	medium
5Y9J	H	0.92	0.97	2.22	**high**	0.78	0.88	5.22	medium
	L	0.91	0.92	2.22	**high**	0.76	0.77	5.22	medium
7C88	H	0.08	0.11	27.73	incorrect	0.03	0.00	82.20	incorrect
	L	0.05	0.00	27.73	incorrect	0.03	0.00	82.20	incorrect
7E9B	H	0.91	0.95	1.93	**high**	0.82	0.93	9.20	**high**
	L	0.91	0.96	1.93	**high**	0.75	0.83	9.20	medium
8Y31	H	0.05	0.00	81.73	incorrect	0.04	0.00	76.16	incorrect
	L	0.05	0.00	81.73	incorrect	0.08	0.12	76.16	incorrect

a RMSD value of each antibody is also provided. The highest DockQ and *f*_nat_ values in the heavy and light chains, the lowest RMSD values, and the best quality predictions (High-quality) are written in bold. The average values for the DockQ and *f*_nat_ are provided at the end of the table (H: heavy chain and L: light chain).

The comparison between EPIGUIDE and AlphaFold3 was based on the RMSD and DockQ values rather than computational speed. AlphaFold3 is an end-to-end deep learning system that generates complete antibody–antigen complex structures in a single integrated run, with runtime determined primarily by the model’s internal computations. In contrast, the EPIGUIDE workflow consists of two main computational phases: (1) ESSA analysis for epitope prediction and (2) ClusPro docking for Ab/Ag complex structure generation. These phases differ fundamentally in their computational requirements and controllability, in contrast to AlphaFold3. The ESSA calculations were performed to determine potential epitopes using three cutoff values and 20 modes for each protein, for a total of 60 calculations using a desktop with AMD Ryzen 7 7800X3D seventh Gen 8 Cores in our lab. For a protein with 195 residues, these calculations resulted in around 3.5 min, which can be further optimized by adjusting the ESSA calculation architecture, i.e., inclusion of GPU acceleration. On the other hand, ClusPro docking calculations were performed on their web server; therefore, the controllability of the server was not possible. Jobs may complete within minutes or require hours, depending on server availability. Due to these fundamental differences between an integrated end-to-end system (AlphaFold3) versus a hybrid local/server workflow (EPIGUIDE), a direct comparison of computational time would be neither meaningful nor fair. We, therefore, restrict our comparison to prediction accuracy using antibody RMSD, native contact fraction, and DockQ values.

## 4 Conclusion

Interleukin-1β (IL-1β) is central to both protective immunity and pathological states such as autoimmune disorders and cancer when dysregulated. Monoclonal antibodies targeting IL-1β have been recently developed; however, the molecular mechanisms underlying their allosteric effects on IL-1β are not well characterized. We first applied ESSA to apo (antibody-removed) and antibody-bound Interleukin-1β (IL-1β) and identified critical residues that significantly alter the intrinsic dynamics of the antigen and antibodies. ESSA successfully predicted the critical residues in the antigen and antibodies. Then, we used the ESSA-predicted residues to guide molecular docking using ClusPro, leading to improved recovery of the experimentally validated binding pose compared to blind docking alone. The results highlight the value of integrating residue–level interface prediction with docking workflows to enhance the structural modeling of antigen–antibody complexes. On the other hand, conformational sampling of the Gevokizumab antibody using ClustENMD showed that the binding site might not form until antigen binding, suggesting an induced-fit mechanism in which the CDRs’ conformation and the VH-VL orientation change upon antigen binding. Upon complex formation, significant shifts in potential binding sites were observed on both the Ag and Ab components of the complex. More importantly, swapping antibodies to noncognate epitopes leads to unfavorable docking, underscoring the role of critical interface residues in guiding specific binding. Additionally, conventional MD simulations in three replicates revealed that the Ag_ESSA-guided docking pose yielded conformers with an H-bond profile more similar to the crystal complex when compared with the results of the Blind docking pose. Further studies are needed to identify and experimentally validate additional (if any) allosteric sites and gain deeper insight into the mechanisms underlying the dynamics of IL-1β and whole antibody structures, including their constant domains.

We further explored whether ESSA correctly predicts known epitope regions for other antigens. ESSA applied to 10 more antigen structures in addition to IL-1β, and the results were compared with two different epitope prediction tools, DiscoTope-3.0 and SEPPA 3.0. The ESSA predictions showed comparable success rates for the studied antigens.

Lastly, we checked whether a complete blind prediction would be possible using ESSA and ClusPro and proposed a workflow named EPIGUIDE. Our results demonstrate that the EPIGUIDE is effective for predicting antibody–antigen complex structures across a range of immunological targets, as validated on 11 diverse Ab/Ag complexes. Direct comparison with AlphaFold3 in terms of RMSD and DockQ values revealed that EPIGUIDE is highly competitive and achieves dramatic improvements in challenging targets such as 4G6J (2.33 Å vs 53.22 Å) and 8Y31 (9.50 Å vs 76.16 Å). We anticipate that EPIGUIDE will serve as a valuable strategy for antibody design, immunological target analysis, and the broader study of protein–protein interactions with further benchmarking and optimization and experimental validation.

## Supplementary Material









## Data Availability

The data and the information that support the results of this study are given within the article and its Supporting Information.

## Notes

Cartoon representations of aligned IL-1β crystal structures; RMSD value differences between different IL-1β crystal structures; ESSA results of all cutoffs for the Canakinumab-removed IL-1β; ESSA results of all cutoffs for the Gevokizumab-removed IL-1β; ESSA results of all cutoffs for the IgG26-removed IL-1β; ESSA results of all cutoffs for the AAL160-removed IL-1β; common residues that were detected as essential using ESSA in all studied IL-1β-targeting antibodies, Canakinumab, Gevokizumab, IgG26, and AAL160; detected essential residues of all cutoffs for IL-1β-removed IgG26; detected essential residues of all cutoffs for IL-1β-removed Canakinumab; detected essential residues of all cutoffs for IL-1β-removed AAL160; detected essential residues of all cutoffs for IL-1β-removed Gevokizumab; detected essential residues at all cutoffs for Gevokizumab frames generated using ClustENMD; all cutoffs-combined ESSA results for Gevokizumab ClustENMD Frame 299; all cutoffs-combined ESSA results for Gevokizumab ClustENMD Frame 219; all cutoffs-combined ESSA results for Gevokizumab ClustENMD Frame 136; Structural comparison of ClustENMD-generated Gevokizumab frames with the corresponding original conformation: (a) Frame 299, (b) Frame 219, and (c) Frame 136, and the vectoral representations are provided, respectively, in the d to e panels; combined-cutoff ESSA result comparison for the unbound (Ab-removed) and bound forms of IL-1βs; ESSA result comparison between the bound and unbound state of IL-1β part of Canakinumab:IL-1β complex (4G6J) at all cutoffs; ESSA result comparison between the bound and unbound state of IL-1β part of Gevokizumab:IL-1β (4G6M) at all cutoffs; ESSA result comparison between the bound and unbound state of IL-1β part of IgG26:IL-1β (7CHY) at all cutoffs; ESSA result comparison between the bound and unbound state of IL-1β part of AAL160: IL-1β (7Z4T) at all cutoffs; combined-cutoff ESSA results for the bound Abs; ESSA results comparing the bound and unbound state of Canakinumab (4G6J) at all cutoffs; ESSA results comparing the bound and unbound state of Gevokizumab (4G6M) at all cutoffs; ESSA results comparing the bound and unbound state of IgG26 (7CHY) at all cutoffs; ESSA results comparing the bound and unbound states of AAL160 (7Z4T) at all cutoffs; the best docking poses for IgG26 (a, b, and c) and AAL160 (d, e, and f); Gevokizumab swapped docking poses; the RMSD profiles of the Gevokizumab-bound IL-1β Ag_ESSA and Blind docking poses; the RMSD profiles comparison of the Ag_ESSA and Blind frames relative to the crystal structure; formed H-Bonds between Ag and Ab above or equal to the 50% threshold in Ag_ESSA three replica MD runs; formed H-Bonds between Ag and Ab above or equal to the 50% threshold in Blind three replica MD runs; selected essential residues for ESSA-guided docking; the Elbow Method; Canakinumab/Ag (4G6J) interactions in the best Blind docking pose; Canakinumab/Ag (4G6J) interactions in the best HCDR_ESSA and Ag_ESSA docking poses; Gevokizumab/Ag (4G6M) interactions in the best Blind docking pose; Gevokizumab/Ag (4G6M) interactions in the best HCDR_ESSA docking pose; Gevokizumab/Ag (4G6M) interactions in the best Ag_ESSA docking pose; IgG26:Ag (7CHY) interactions in the best Blind docking pose; IgG26:Ag (7CHY) interactions in the best HCDR_ESSA docking pose; IgG26:Ag (7CHY) interactions in the best Ag_ESSA docking pose; AAL160:Ag (7Z4T) interactions in the best Blind and HCDR_ESSA docking pose; AAL160:Ag (7Z4T) interactions in the best Ag_ESSA docking pose; and selected essential residues for Max and Ave Epitopes (PDF)
MD Input Files for the 4G6M-Ag_ESSA (ZIP)
MD Input Files for the 4G6M-Blind (ZIP)
Best docking poses of each structure from the knowledge-based and blind (EPIGUIDE) (ZIP)

## Abbreviations and symbols:

IL-1β: interleukin-1 beta
IL-1R: interleukin-1 receptor
ESSA: essential site scanning analysis
CDR: complementarity-determining regions
Ab: antibody
Ag: antigen
H: heavy
L: light
mAb: monoclonal antibody
aa: amino acid
VH: variable region of the heavy chain
VL: variable region of the light chain
MD: molecular dynamics.
