# Supplementary material for: Integrative Computational Prediction Strategy for Antibody–Antigen Binding: A Case Study on Interleukin‑1 Beta
Source: J Chem Inf Model. 2026 May 27;66(11):6659–75. doi: 10.1021/acs.jcim.6c00781 (PMC13250912; doi:10.1021/acs.jcim.6c00781)
Supplement: Supplementary file 1 [file ci6c00781_si_001.pdf]

Supplementary Material

Integrative Computational Prediction Strategy for Antibody-Antigen Binding: A Case Study on Interleukin-1 Beta

Mehmet Emin Aygen [a] and Arzu Uyar [a,b] *

[a] Department of Bioengineering, Izmir Institute of Technology, 35430, Urla, Izmir, Türkiye

[b] Computational Science and Engineering Program, Izmir Institute of Technology, 35430, Urla, Izmir, Türkiye

*Corresponding Author e-mail: arzuuyar@iyte.edu.tr

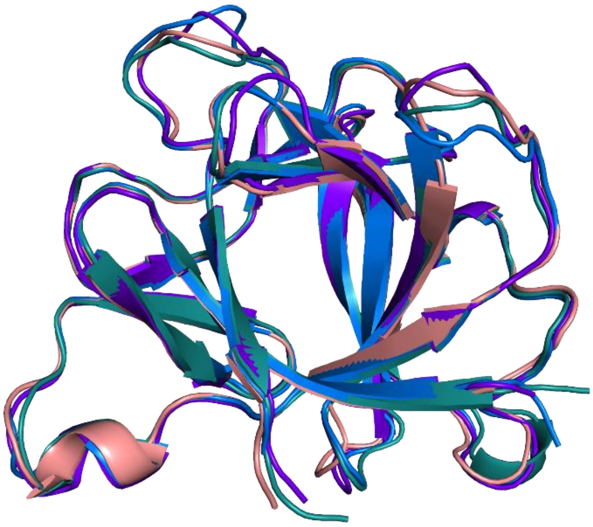

Figure S1. Cartoon representations of aligned IL-1β crystal structures. (teal: 4G6J, blue: 4G6M, purple: 7CHY, and salmon: 7Z4T). (All structure figures in this study were prepared using PyMOL v2.5.4.)

Table S1. RMSD value differences between different IL-1β crystal structures.

	**4G6J**	**4G6M**	**7CHY**	**7Z4T**
**4G6J (Canakinumab:IL-1β)**	0	1.57 Å	1.41 Å	1.38 Å
**4G6M (Gevokizumab:IL-1β)**	1.57 Å	0	1.62 Å	1.57 Å
**7CHY (IgG26:IL-1β)**	1.41 Å	1.62 Å	0	1.39 Å
**7Z4T (AAL160:IL-1β)**	1.38 Å	1.57 Å	1.39 Å	0

Table S2. ESSA results of all cutoffs for the Canakinumab-removed IL-1β. Bold numbers on the CutOff column indicate the cutoff at which the Highest z-score was obtained.

**4G6J - Antigen - AllCutOffs**				
**Residue**	**Residue Type**	**Interaction**	**Z-score (The Highest)**	**CutOff (Å)**
7 - N	Polar Uncharged	No Interaction	7	7.3
10 - L	Hydrophobic		6	7.3 - 10 - 13
11 - R	Positively Charged		7	10 - 13
16 - K	Positively Charged		6	7.3
18 - L	Hydrophobic		7	13
24 - Y	Hydrophobic		6	7.3
26 - L	Hydrophobic		6	7.3 - 10
31 - L	Hydrophobic	**H Chain 31-V**	6	10
36 - M	Hydrophobic	**No Interaction**	6	13
38 - Q	Polar Uncharged	**H Chain 31-V / 32-Y / 100-L / 101-R**	6	13
39 - Q	Polar Uncharged	**H Chain 101-R**	7	7.3 - 10 - 13
40 - V	Hydrophobic	No Interaction	6	7.3 - 10
42 - F	Hydrophobic		7	7.3 - 10 - 13
44 - M	Hydrophobic		6	10
62 - L	Hydrophobic		6	7.3
68 - Y	Hydrophobic		7	7.3 - 10
69 - L	Hydrophobic		6	10 - 13
71 - C	Polar Uncharged		6	13
72 - V	Hydrophobic		6	13
74 - K	Positively Charged		7	13
77 - K	Positively Charged		7	13
78 - P	Hydrophobic		6	13
79 - T	Polar Uncharged		7	13
80 - L	Hydrophobic		7	7.3 - **13**
81 - Q	Polar Uncharged		7	13
82 - L	Hydrophobic		7	**7.3** - 10
90 - Y	Hydrophobic		7	10
91 - P	Hydrophobic		6	10
92 - K	Positively Charged		6	10
99 - F	Hydrophobic		6	10
101 - F	Hydrophobic		7	**7.3 - 10** - 13
103 - K	Positively Charged		6	13
111 - E	Negatively Charged		6	10
112 - F	Hydrophobic		7	7.3 - **10** - 13
117 - F	Hydrophobic		7	13
120 - W	Hydrophobic		7	7.3 - 13

122 - I	Hydrophobic		6	7.3 - 10 - 13
132 - V	Hydrophobic		6	7.3
133 - F	Hydrophobic		6	13
134 - L	Hydrophobic		7	7.3 - 13
145 - D	Negatively Charged		6	10
146 - F	Hydrophobic		6	7.3 - 10 - 13
151 - V	Hydrophobic		6	7.3

Table S3. ESSA results of all cutoffs for the Gevokizumab-removed IL-1β. Bold numbers on the CutOff column indicate the cutoff at which the Highest z-score was obtained.

**4G6M - Antigen - AllCutOffs**				
**Residue**	**Residue Type**	**Interaction**	**Z-score (The Highest)**	**CutOff (Å)**
3 - V	Hydrophobic	**No Interaction**	6	7.3
10 - L	Hydrophobic		6	10 - 13
11 - R	Positively Charged		6	10
18 - L	Hydrophobic		6	13
26 - L	Hydrophobic		6	10
31 - L	Hydrophobic		6	7.3
36 - M	Hydrophobic		6	10
39 - Q	Polar Uncharged		7	**7.3 - 10** - 13
40 - V	Hydrophobic		6	7.3 - 10 - 13
41 - V	Hydrophobic		6	10
42 - F	Hydrophobic		7	7.3 - 10 - 13
44 - M	Hydrophobic		6	10
60 - L	Hydrophobic		6	13
68 - Y	Hydrophobic		7	7.3 - 10
69 - L	Hydrophobic		6	10 - 13
71 - C	Polar Uncharged		6	13
72 - V	Hydrophobic	**H Chain 104-P / L Chain 32-Y / 50-Y**	6	13
77 - K	Positively Charged	**No Interaction**	6	13
78 - P	Hydrophobic		6	13
79 - T	Polar Uncharged		6	13
80 - L	Hydrophobic		7	7.3 - **13**
81 - Q	Polar Uncharged	**L Chain 50-Y**	7	13
82 - L	Hydrophobic	**No Interaction**	7	**7.3** - 10
90 - Y	Hydrophobic	**L Chain 93-M**	7	10
91 - P	Hydrophobic	**No Interaction**	7	7.3 - 10
92 - K	Positively Charged	**L Chain 93-M**	7	10
93 - K	Positively Charged	**No Interaction**	6	7.3
99 - F	Hydrophobic		6	10
101 - F	Hydrophobic		7	**7.3 - 10** - 13
111 - E	Negatively Charged		6	10
112 - F	Hydrophobic		7	7.3 - 10 - 13
117 - F	Hydrophobic	**H Chain 102-Y**	7	7.3 - **13**
120 - W	Hydrophobic	**No Interaction**	7	7.3 - 13
121 - Y	Hydrophobic		6	10
122 - I	Hydrophobic		6	10 - 13

132 - V	Hydrophobic		7	7.3
134 - L	Hydrophobic		6	7.3 - 13
144 - T	Polar Uncharged		6	7.3 - 10
146 - F	Hydrophobic		7	7.3 - **10**

Table S4. ESSA results of all cutoffs for the IgG26-removed IL-1β. Bold numbers on the CutOff column indicate the cutoff at which the Highest z-score was obtained.

7CHY - Antigen - AllCutOffs				
**Residue**	**Residue Type**	**Interaction**	**Z-score (The Highest)**	**CutOff (Å)**
122 - L	Hydrophobic	**No Interaction**	6	10
123 - N	Polar Uncharged		6	10
124 - C	Polar Uncharged		6	10
125 - T	Polar Uncharged		6	10
126 - L	Hydrophobic		6	10 - 13
127 - R	Positively Charged		7	10 - **13**
132 - K	Positively Charged		6	7.3
134 - L	Hydrophobic		7	13
135 - V	Hydrophobic		7	13
142 - L	Hydrophobic		6	10
145 - L	Hydrophobic	**H Chain 57.F**	6	13
147 - L	Hydrophobic	**H Chain 57.F / 59.Y**	7	7.3 - **10**
152 - M	Hydrophobic	**No Interaction**	6	13
153 - E	Negatively Charged		6	13
154 - Q	Polar Uncharged	**H Chain 57.F**	7	7.3 - **13**
155 - Q	Polar Uncharged	**No Interaction**	7	7.3 - 10 - 13
156 - V	Hydrophobic		7	7.3 - **10 - 13**
157 - V	Hydrophobic		7	10
158 - F	Hydrophobic		7	7.3 - 10 - 13
176 - L	Hydrophobic		6	10
184 - Y	Hydrophobic		7	7.3 - 10
185 - L	Hydrophobic		6	10 - 13
187 - C	Polar Uncharged		6	13
188 - V	Hydrophobic		6	13
194 - P	Hydrophobic		6	13
195 - T	Polar Uncharged		6	13
196 - L	Hydrophobic		7	7.3 - 10 - **13**
197 - Q	Polar Uncharged		6	13
198 - L	Hydrophobic		6	7.3
206 - Y	Hydrophobic		7	10
207 - P	Hydrophobic		7	10
215 - F	Hydrophobic		6	10
217 - F	Hydrophobic		7	7.3 - 10
228 - F	Hydrophobic		7	**7.3 - 10** - 13
233 - F	Hydrophobic		7	13
236 - W	Hydrophobic		7	7.3 - 13
250 - L	Hydrophobic		7	7.3 - **13**
262 - F	Hydrophobic		6	7.3 - 10

Table S5. ESSA results of all cutoffs for the AAL160-removed IL-1β. Bold numbers on the CutOff column indicate the cutoff at which the Highest z-score was obtained.

7Z4T - Antigen - AllCutOffs				
**Residue**	**Type**	**Interaction**	**Z-score (The Highest)**	**CutOff (Å)**
6 - L	Hydrophobic		6	10
7 - N	Polar Uncharged		7	7.3
10 - L	Hydrophobic		7	**10** - 13
11 - R	Positively Charged	**No Interaction**	7	10 - **13**
16 - K	Positively Charged		6	7.3
18 - L	Hydrophobic		6	13
19 - V	Hydrophobic		6	13
24 - Y	Hydrophobic	**H Chain 32.Y / 33.Q / 99.Y / 100.T / 101.N**	6	7.3
26 - L	Hydrophobic		6	10
31 - L	Hydrophobic		6	10
39 - Q	Polar Uncharged		7	7.3 - 10 - 13
40 - V	Hydrophobic		7	7.3 - 10 - 13
41 - V	Hydrophobic		7	7.3 - 10
42 - F	Hydrophobic	**No Interaction**	7	7.3 - 10 - 13
63 - K	Positively Charged		7	7.3
68 - Y	Hydrophobic		7	7.3 - 10
69 - L	Hydrophobic		7	7.3 - **10** - 13
71 - C	Polar Uncharged		6	13
72 - V	Hydrophobic		6	13
74 - K	Positively Charged	**L Chain 32.Y**	6	13
77 - K	Positively Charged		6	13
79 - T	Polar Uncharged	**No Interaction**	6	13
80 - L	Hydrophobic		7	7.3 - 10 - **13**
81 - Q	Polar Uncharged		7	13
82 - L	Hydrophobic	**H Chain 101.N**	7	7.3
90 - Y	Hydrophobic		6	7.3 - 10
101 - F	Hydrophobic		7	**7.3 - 10** - 13
104 - I	Hydrophobic		7	10
105 - E	Negatively Charged		6	10
110 - L	Hydrophobic		6	10
111 - E	Negatively Charged	**No Interaction**	7	7.3 - **10**
112 - F	Hydrophobic		7	7.3 - **10 - 13**
117 - F	Hydrophobic		7	7.3 - **13**
120 - W	Hydrophobic		7	7.3 - **13**
122 - I	Hydrophobic		6	10
132 - V	Hydrophobic		6	7.3

134 - L	Hydrophobic		7	13
143 - I	Hydrophobic		6	7.3
145 - D	Negatively Charged		6	10
146 - F	Hydrophobic		6	7.3 - 10

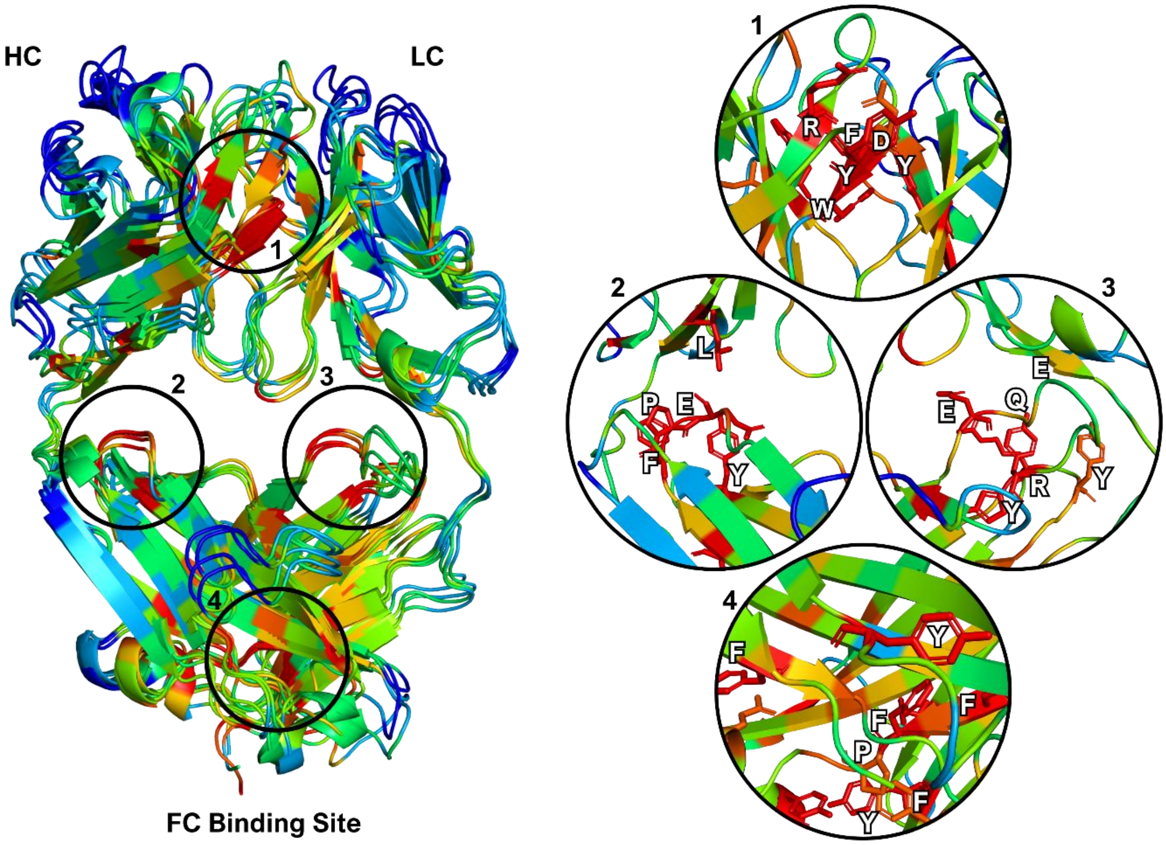

Figure S2. Common residues that were detected as essential using ESSA in all studied IL-1β-targeting antibodies, Canakinumab, Gevokizumab, IgG26, and AAL160. Region 1: VH-VL interface, region 2 and 3: near the center of H and L chains, and region 4: CH-CL interface.

Table S6. Detected essential residues of all cutoffs for IL-1β-removed IgG26. Bold numbers on the CutOff column indicate the cutoff at which the Highest z-score was obtained. *CDR1 **CDR2 ***CDR3.

7CHY - Antibody AllCutOffs				
**Residue (H Chain)**	**Residue Type**	**Interaction**	**Z-score (The Highest)**	**CutOff**
11 - L	Hydrophobic	No Interaction	6	7.3
32 - Y*	Hydrophobic		6	13
35 - H*	Positively Charged		7	13
36 - W	Hydrophobic		7	13
47 - W	Hydrophobic		7	10 - **13**
52 - W**	Hydrophobic	**Ag 244.E / 245.N**	6	13
94 - Y	Hydrophobic	No Interaction	6	10 - 13
98 - R	Positively Charged		7	13
99 - F***	Hydrophobic	**Ag 243.A**	7	10 - **13**
105 - Y***	Hydrophobic	No Interaction	7	13
106 - I***	Hydrophobic		6	13
107 - M***	Hydrophobic		6	13
108 - D***	Negatively Charged		7	13
109 - Y***	Hydrophobic		6	13
110 - W	Hydrophobic		7	10 - **13**
115 - L	Hydrophobic		7	**7.3** - 10
117 - T	Polar Uncharged		6	7.3
129 - F	Hydrophobic		7	**10** - 13
136 - K	Positively Charged		6	13
145 - L	Hydrophobic		6	13
150 - K	Positively Charged		7	**10** - 13
153 - Y	Hydrophobic		7	7.3 - 10 - 13
154 - P	Hydrophobic		6	7.3
155 - E	Negatively Charged		7	10 - 13
183 - Y	Hydrophobic		7	10 - 13
191 - V	Hydrophobic		7	13
192 - P	Hydrophobic		6	13
196 - L	Hydrophobic		6	13
201 - Y	Hydrophobic		7	10 - **13**
**L Chain**				
35 - W	Hydrophobic	No Interaction	6	13
36 - Y	Hydrophobic		6	13
49 - Y	Hydrophobic		6	13
55 - Y**	Hydrophobic		6	13
62 - F	Hydrophobic		6	13
83 - F	Hydrophobic		7	**7.3** - 10
86 - Y	Hydrophobic		7	10 - **13**
91 - Y***	Hydrophobic		7	13
103 - K	Positively Charged		6	13

105 - E	Negatively Charged		6	10
116 - F	Hydrophobic		7	10 - 13
118 - F	Hydrophobic		6	10 - 13
124 - Q	Polar Uncharged		6	10
140 - Y	Hydrophobic		6	7.3 - 10 - 13
148 - W	Hydrophobic		6	10 - 13
160 - Q	Polar Uncharged		6	10
165 - E	Negatively Charged		7	10 - 13
166 - Q	Polar Uncharged		6	7.3 - 10 - 13
173 - Y	Hydrophobic		7	10 - **13**
179 - L	Hydrophobic		6	10 - 13
180 - T	Polar Uncharged		6	10 - 13
181 - L	Hydrophobic		7	13
186 - Y	Hydrophobic		6	10 - 13
192 - Y	Hydrophobic		7	10 - 13
195 - E	Hydrophobic		6	13

Table S7. Detected essential residues of all cutoffs for IL-1β-removed Canakinumab. Bold numbers on the CutOff column indicate the cutoff at which the Highest z-score was obtained. * CDR1 **CDR2 ***CDR3.

4G6J - Antibody AllCutOffs				
**Residue (H Chain)**	**Residue Type**	**Interaction**	**Z-score (The Highest)**	**CutOff (Å)**
11 - V	Hydrophobic	No Interaction	7	7.3
27 - F	Hydrophobic		6	10
36 - W	Hydrophobic		6	13
47 - W	Hydrophobic		7	10 - **13**
94 - Y	Hydrophobic		6	13
95 - Y	Hydrophobic		6	13
98 - R	Positively Charged	**Ag 37-E**	7	**10** - 13
105 - F***	Hydrophobic	No Interaction	7	10 - 13
106 - D***	Negatively Charged		6	13
107 - Y***	Hydrophobic		7	10 - 13
108 - W	Hydrophobic		7	10 - 13
113 - L	Hydrophobic		7	**7.3** - 10 - 13
127 - F	Hydrophobic		7	10 - 13
129 - L	Hydrophobic		6	10
146 - L	Hydrophobic		6	10 - 13
148 - K	Positively Charged		7	10 - 13
151 - F	Hydrophobic		7	**7.3 - 10** - 13
152 - P	Hydrophobic		7	**7.3** - 10
153 - E	Negatively Charged		7	7.3 - 10 - 13
154 - P	Hydrophobic		6	10 - 13
169 - H	Positively Charged		7	**7.3 - 10** - 13
171 - F	Hydrophobic		6	10 - 13
181 - Y	Hydrophobic		7	10 - 13
199 - Y	Hydrophobic		7	7.3 - 10
**L Chain**				
36 - L	Hydrophobic	No Interaction	7	13
41 - D	Negatively Charged		7	13
62 - F	Hydrophobic		6	10
86 - Y	Hydrophobic		7	10 - **13**
87 - Y	Hydrophobic		6	13
89 - H***	Positively Charged		6	13
98 - F	Hydrophobic		6	13
116 - F	Hydrophobic		7	**7.3 - 10** - 13
117 - I	Hydrophobic		6	7.3
118 - F	Hydrophobic		7	7.3 - **10 - 13**
119 - P	Hydrophobic		6	13
120 - P	Hydrophobic		6	13
124 - Q	Polar Uncharged		6	10
132 - V	Hydrophobic		6	13

137 - N	Polar Uncharged		6	10
140 - Y	Hydrophobic		6	7.3 - 13
142 - R	Positively Charged		7	13
148 - W	Hydrophobic		7	7.3 - 10 - **13**
160 - Q	Polar Uncharged		6	10
165 - E	Negatively Charged		7	7.3 - **10 - 13**
173 - Y	Hydrophobic		7	7.3 - 10 - **13**
179 - L	Hydrophobic		6	10 - 13
180 - T	Polar Uncharged		7	13
181 - L	Hydrophobic		6	10 - 13
186 - Y	Hydrophobic		7	**10** - 13
192 - Y	Hydrophobic		7	7.3 - **10 - 13**
209 - F	Hydrophobic		6	7.3 - 13

Table S8. Detected essential residues of all cutoffs for IL-1β-removed AAL160. Bold numbers on the CutOff column indicate the cutoff at which the Highest z-score was obtained. ***CDR3.

7Z4T - Antibody AllCutOffs				
**Residue (H Chain)**	**Residue Type**	**Interaction**	**Z-score (The Highest)**	**CutOff**
36 - W	Hydrophobic		6	13
47 - W	Hydrophobic		6	13
83 - W	Hydrophobic	**No Interaction**	6	13
94 - Y	Hydrophobic		6	13
95 - Y	Hydrophobic		6	13
99 - Y***	Hydrophobic	**Ag 23.P / 24.Y**	6	13
105 - F***	Hydrophobic		6	13
108 - W	Hydrophobic		7	10 - **13**
127 - F	Hydrophobic		6	10 - 13
131 - P	Hydrophobic		6	13
132 - S	Positively Charged		6	13
133 - S	Polar Uncharged		7	10
134 - K	Positively Charged	**No Interaction**	6	13
148 - K	Positively Charged		6	10 - 13
151 - F	Hydrophobic		7	**7.3** - 10 - 13
153 - E	Negatively Charged		7	10 - **13**
169 - H	Positively Charged		6	7.3 - 10 - 13
181 - Y	Hydrophobic		7	10 - **13**
199 - Y	Hydrophobic		6	13
218 - P	Hydrophobic		7	10 - **13**
**L Chain**				
36 - Y	Hydrophobic		6	13
105 - E	Negatively Charged		7	**7.3** - 10 - 13
116 - F	Hydrophobic		7	7.3 - 10 - 13
118 - F	Hydrophobic		7	7.3 - 10 - 13
119 - P	Hydrophobic		7	7.3 - **13**
120 - P	Hydrophobic		6	13
122 - D	Negatively Charged		6	10 - 13
140 - Y	Hydrophobic		7	**7.3** - 13
142 - R	Positively Charged		6	13
165 - E	Negatively Charged		7	10 - 13
166 - Q	Polar Uncharged	**No Interaction**	7	**7.3** - 10 - **13**
173 - Y	Hydrophobic		7	10 - **13**
186 - Y	Hydrophobic		7	7.3
192 - Y	Hydrophobic		6	7.3
207 - K	Positively Charged		6	13
209 - F	Hydrophobic		7	7.3 - 10 - **13**
210 - N	Polar Uncharged		6	10
211 - R	Positively Charged		7	**10** - 13
212 - G	Hydrophobic		6	10
213 - E	Negatively Charged		6	10
214 - C	Polar Uncharged		7	10

Table S9. Detected essential residues of all cutoffs for IL-1β-removed Gevokizumab. Bold numbers on the CutOff column indicate the cutoff at which the Highest z-score was obtained. **CDR2 ***CDR3.

4G6M - Antibody AllCutOffs			
**Residue (H Chain)**	**Residue Type**	**Z-score (The Highest)**	**CutOff (Å)**
4 - L	Hydrophobic	6	13
24 - F	Hydrophobic	7	13
27 - F	Hydrophobic	7	13
38 - W	Hydrophobic	6	13
40 - R	Positively Charged	6	10
49 - W	Hydrophobic	6	10 - 13
95 - Y	Hydrophobic	6	13
96 - F	Hydrophobic	6	13
99 - R	Positively Charged	7	13
107 - F***	Hydrophobic	6	13
108 - V***	Hydrophobic	7	13
109 - D***	Negatively Charged	7	13
110 - W	Hydrophobic	7	10 - **13**
115 - L	Hydrophobic	6	7.3 - 13
129 - F	Hydrophobic	7	10
145 - L	Hydrophobic	6	13
150 - K	Positively Charged	7	10
153 - F	Hydrophobic	7	**7.3** - 13
154 - P	Hydrophobic	6	7.3 - 10
155 - E	Negatively Charged	7	7.3 - 10 - 13
156 - P	Hydrophobic	6	10 - 13
171 - H	Positively Charged	6	7.3 - 10 - 13
173 - F	Hydrophobic	6	13
183 - Y	Hydrophobic	7	13
201 - Y	Hydrophobic	6	10 - 13
**L Chain**			
36 - Y	Hydrophobic	6	13
55 - H**	Positively Charged	6	13
62 - F	Hydrophobic	6	10
83 - F	Hydrophobic	6	10 - 13
96 - W***	Hydrophobic	6	10 - 13
105 - E	Negatively Charged	6	10
116 - F	Hydrophobic	7	7.3 - 10 - 13
117 - I	Hydrophobic	6	7.3 - 10
118 - F	Hydrophobic	7	7.3 - 10 - 13
119 - P	Hydrophobic	7	10
124 - Q	Polar Uncharged	6	10
137 - N	Polar Uncharged	6	13
140 - Y	Hydrophobic	6	13
142 - R	Positively Charged	6	13

148 - W	Hydrophobic	6	7.3 - 10
165 - E	Negatively Charged	7	10 - 13
166 - Q	Polar Uncharged	6	10 - 13
173 - Y	Hydrophobic	7	10 - **13**
186 - Y	Hydrophobic	7	7.3 - 10
192 - Y	Hydrophobic	7	**7.3 - 10** - 13
209 - F	Hydrophobic	7	7.3 - 10
211 - R	Positively Charged	6	10

Table S10. Detected essential residues at all cutoffs for Gevokizumab frames generated using ClustENMD. Bold numbers on the CutOff column indicate the cutoff at which the Highest z-score was obtained. ***CDR3.

4G6M - Antibody ClustENMD AllCutOffs			
**Residue (H Chain)**	**Frame-299 (RMSD = 6.98 Å)**	**Frame-219 (RMSD = 5.94 Å)**	**Frame-136 (RMSD = 5.55 Å)**
11 - L	-	10 - 13	-
27 - F	10	-	10
38 - W	-	-	13
40 - R	-	-	10 - 13
49 - W	-	-	13
95 - Y	-	-	13
99 - R	10	-	10 - 13
107 - F***	-	-	13
109 - D***	-	-	10
110 - W	13	-	10 - **13**
115 - L	-	-	10
129 - F	-	-	**10** - 13
136 - K	-	13	-
145 - L	-	-	13
150 - K	-	-	10 - 13
153 - F	10 - 13	10 - 13	7.3 - 10 - 13
154 - P	-	-	10
155 - E	-	-	10 - 13
171 - H	-	-	13
173 - F	-	-	10
177 - L	10 - 13	-	-
183 - Y	10 - **13**	13	10 - 13
201 - Y	-	-	13
207 - H	7.3	-	-
**L Chain**			
36 - Y	-	-	13
45 - K	-	-	10
83 - F	-	**7.3** - 10 - 13	7.3 - 10 - **13**
86 - Y	-	-	10 - 13
96 - W***	-	-	13
105 - E	**7.3 - 10** - 13	**7.3 - 10** - 13	7.3 - 10 - 13
106 - I	10	-	-
108 - R	7.3 - 10	7.3	-
116 - F	-	-	**10** - 13
118 - F	-	-	10 - 13
124 - Q	-	-	10
139 - F	-	-	13
140 - Y	7.3 - 10	**7.3** - 10	7.3 - 10 - 13
142 - R	7.3 - **10 - 13**	10 - 13	10 - 13
143 - E	-	-	7.3
148 - W	-	-	10 - **13**
165 - E	10 - 13	13	10 - 13
166 - Q	7.3 - 10	7.3 - 10 - 13	7.3 - 10 - 13
173 - Y	10 - **13**	10 - **13**	7.3 - 10 - **13**

179 - L	-	-	13
180 - T	-	-	13
181 - L	-	-	13
186 - Y	10 - 13	13	-
189 - H	-	-	13
190 - K	10 - 13	13	-
191 - V	10 - 13	13	-
192 - Y	10 - 13	10 - **13**	10 - 13
208 - S	13	13	-
209 - F	10 - 13	10 - 13	-
210 - N	13	13	-
211 - R	10 - **13**	13	-

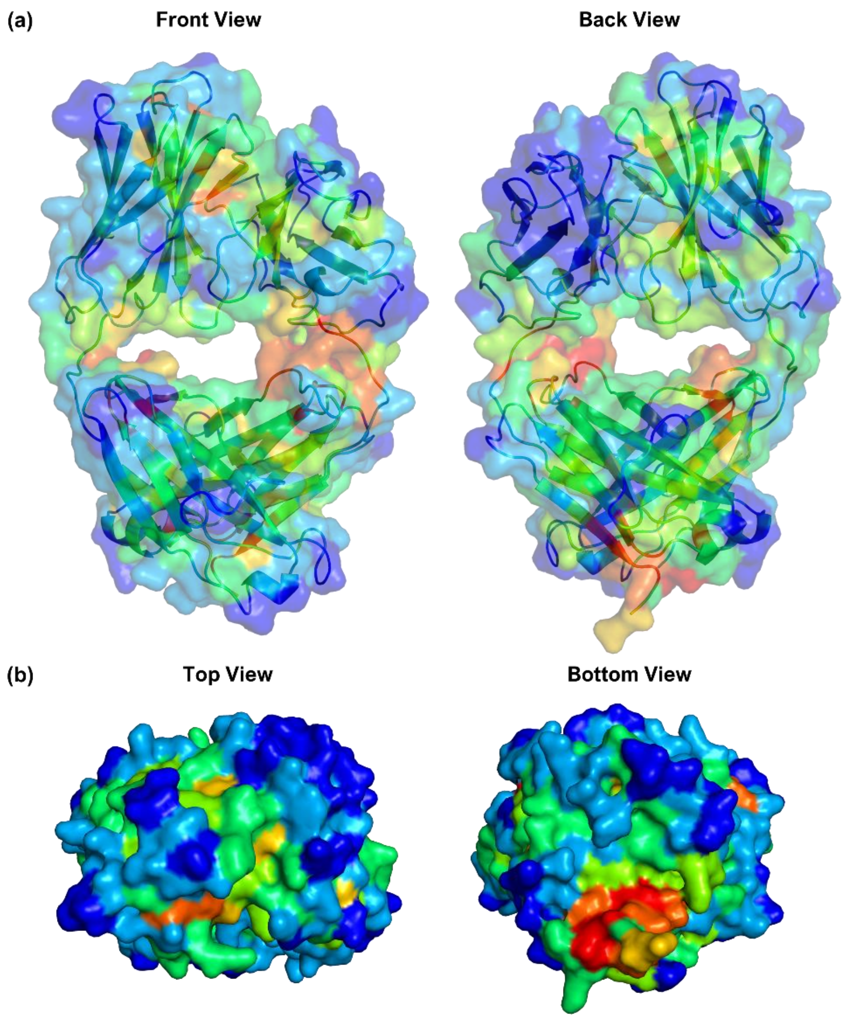

Figure S3. All cutoffs-combined ESSA results for Gevokizumab ClustENMD Frame 299.

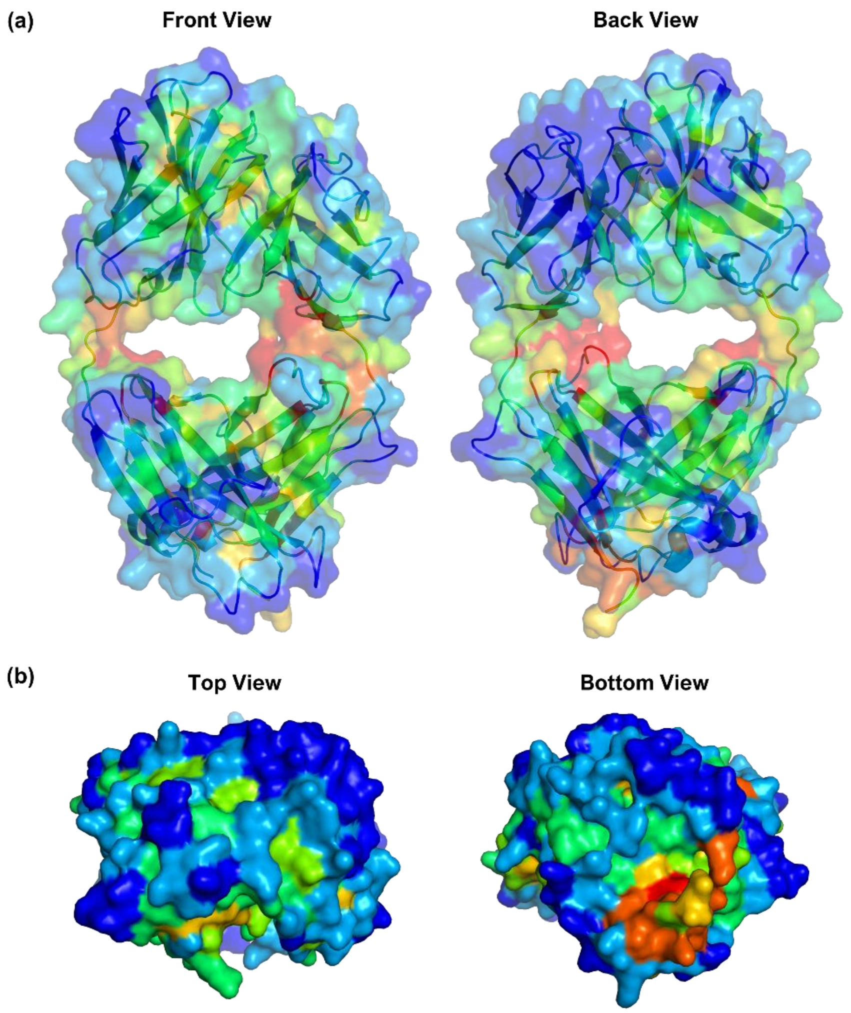

Figure S4. All cutoffs-combined ESSA results for Gevokizumab ClustENMD Frame 219.

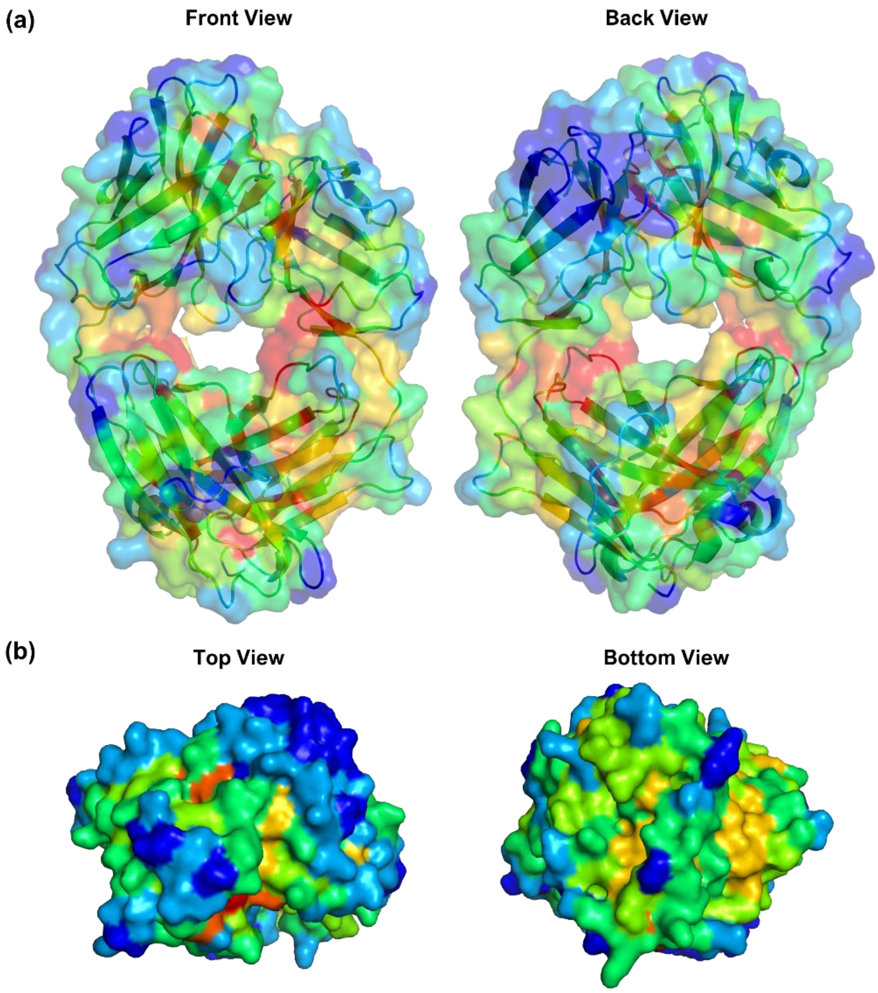

Figure S5. All cutoffs-combined ESSA results for Gevokizumab ClustENMD Frame 136.

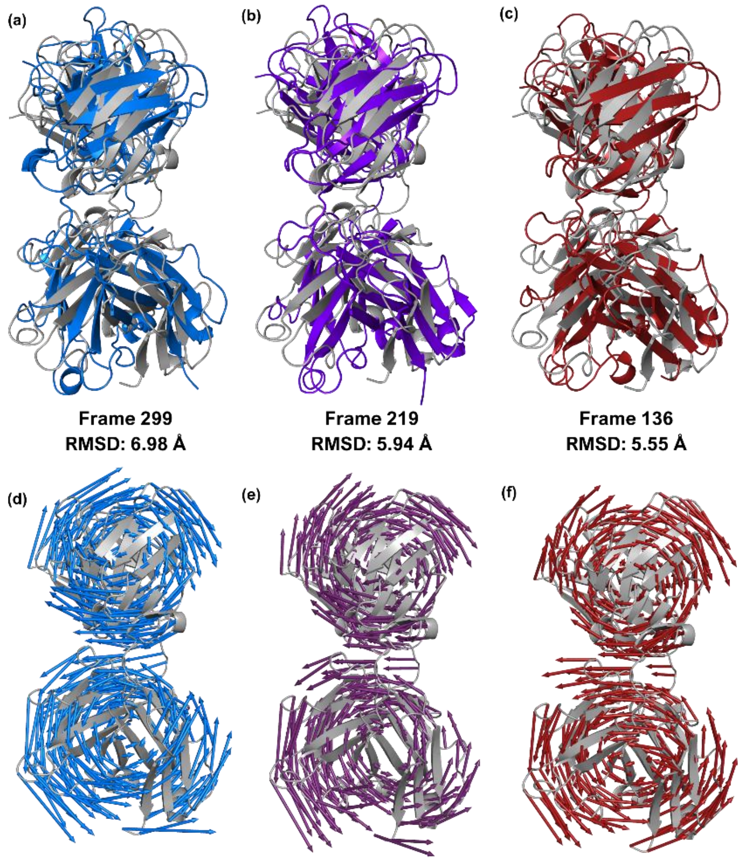

Figure S6. Structural comparison of ClustENMD-generated Gevokizumab frames with the corresponding original conformation: a) Frame 299, b) Frame 219, and c) Frame 136, and the vectoral representations are provided, respectively, in the d to e panels. Structures are given from a side view perspective, clearly showing the counter-rotation of the VH-VL (upper) and CH-CL (lower) parts of Gevokizumab.

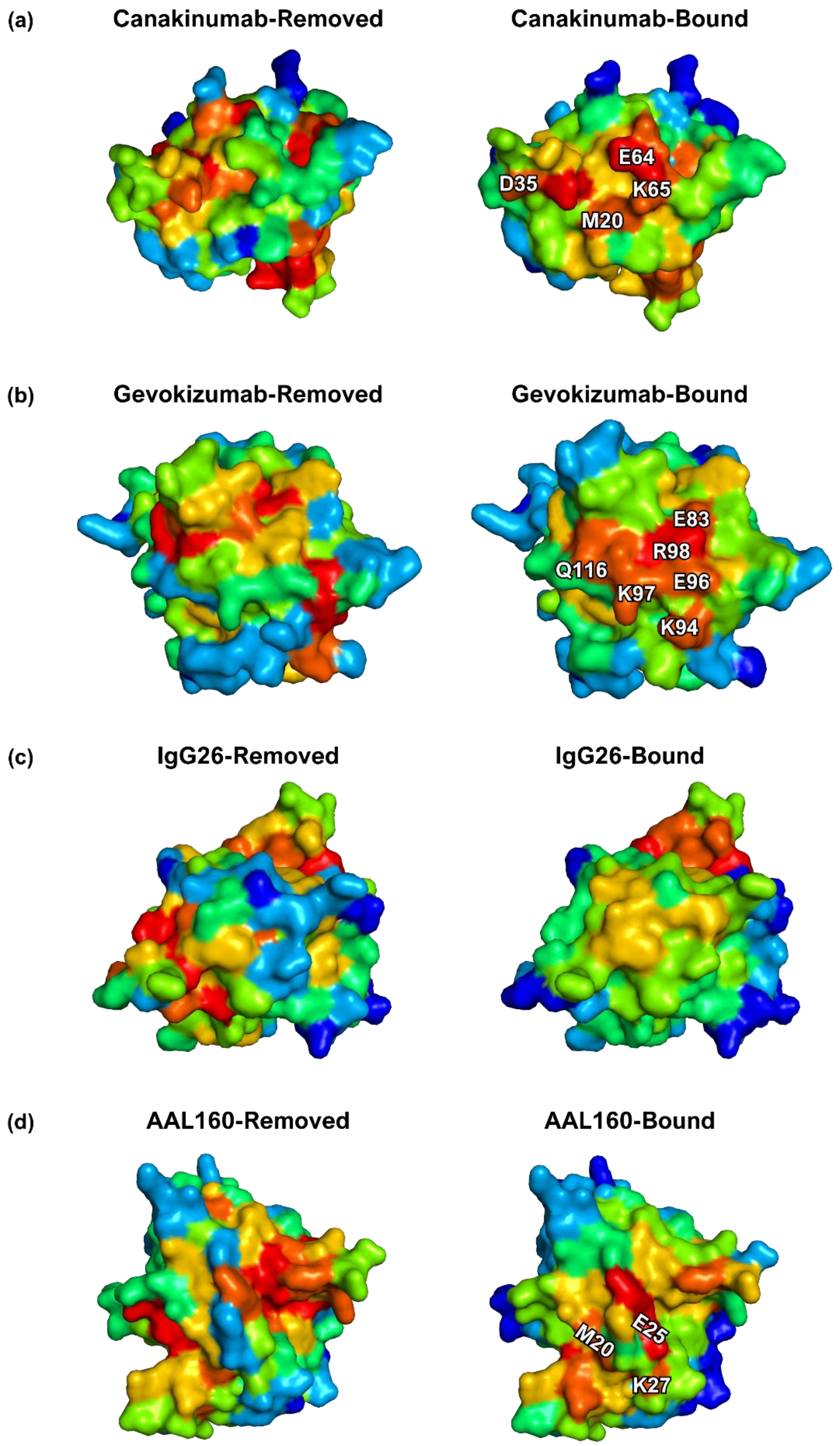

Figure S7. Combined-cutoff ESSA result comparison for the unbound (Ab-removed) and bound forms of IL-1βs.

Table S11. ESSA result comparison between the bound and unbound state of IL-1β part of Canakinumab:IL-1β complex (4G6J) at all cutoffs. Bold numbers indicate a positive change.

4G6J - Antigen&Complex AllCutOffs				
**Residue**	**Interaction**	**Rcut: 7.3 Å Z-score - Ag -> Complex**	**Rcut: 10 Å Z-score - Ag -> Complex**	**Rcut: 13 Å Z-score - Ag -> Complex**
7 - N	No Interaction	7 -> 2	-	-
10 - L		6 -> 3	6 -> 5	6 -> 4
11 - R		-	7 -> 5	7 -> 3
16 - K		6 -> 3	-	-
18 - L		-	-	7 -> 4
20 - M	**H Chain 52.W / 53.Y / 102.T**	-	**4 -> 6**	-
24 - Y	No Interaction	6 -> 3	-	-
26 - L		6 -> 3	6 -> 5	-
31 - L	**H Chain 31.V**	-	6 -> 4	-
35 - D	**H Chain 28.T / 31.V / 32.Y**	**2 -> 6**	-	-
36 - M	**No Interaction**	-	-	6 -> 2
38 - Q	**H Chain 31.V / 32.Y / 100.L / 101.R**	**4 -> 7**	-	6 -> 4
39 - Q	**H Chain 101.R**	7 -> 4	7 -> 5	7 -> 3
40 - V	No Interaction	6 -> 3	6 -> 5	-
42 - F		7 -> 3	7 -> 6	7 -> 5
44 - M		-	6 -> 5	-
62 - L		6 -> 3	-	-
63 - K		-	**3 -> 6**	**3 -> 6**
64 - E	**H Chain 101.R / L Chain 32.S / 50.Y**	-	**4 -> 7**	**4 -> 6**
65 - K	**H Chain 102.T / L Chain 91.S / 92.S / 96.F**	-	**3 -> 6**	**3 -> 6**
68 - Y	No Interaction	7 -> 4	7 -> 6	**5 -> 6**
69 - L		-	-	6 -> 5
71 - C		-	-	6 -> 5
74 - K		-	-	7 -> 6
77 - K		-	-	7 -> 5
79 - T		-	-	7 -> 6
80 - L		6 -> 3	**5 -> 6**	-
82 - L		7 -> 3	6 -> 5	-

90 - Y		-	7 -> 4	-
91 - P		-	6 -> 3	-
92 - K		-	6 -> 3	-
99 - F		-	6 -> 5	-
101 - F		7 -> 5	-	-
111 - E		-	6 -> 5	-
112 - F		6 -> 4	-	-
120 - W		7 -> 5	-	-
122 - I		6 -> 3	-	-
132 - V		6 -> 3	-	-
134 - L		7 -> 4	-	-
145 - D		-	6 -> 4	-
146 - F		6 -> 4	-	-
151 - V		6 -> 2	-	-

Table S12. ESSA result comparison between the bound and unbound state of IL-1β part of Gevokizumab:IL-1β (4G6M) at all cutoffs. Bold numbers indicate a positive change.

4G6M - Antigen&Complex AllCutOffs				
**Residue**	**Interaction**	**Rcut: 7.3 Å Z-score - Ag -> Complex**	**Rcut: 10 Å Z-score - Ag -> Complex**	**Rcut: 13 Å Z-score - Ag -> Complex**
3 - V	**No Interaction**	6 -> 1	-	-
10 - L		-	6 -> 4	-
11 - R		-	6 -> 5	**5 -> 6**
18 - L		-	-	6 -> 5
26 - L		-	6 -> 3	-
29 - L		-	-	**4 -> 6**
30 - H		-	-	**3 -> 6**
31 - L		6 -> 2	-	-
36 - M		-	6 -> 4	-
37 - E		-	-	**4 -> 6**
38 - Q		-	-	**4 -> 6**
39 - Q		7 -> 3	6 -> 5	**6 -> 7**
40 - V		6 -> 2	6 -> 4	6 -> 4
41 - V		-	6 -> 3	-
42 - F		7 -> 3	7 -> 4	7 -> 6
44 - M		-	6 -> 4	-
60 - L		-	-	6 -> 5
68 - Y		7 -> 3	7 -> 4	-
69 - L		-	6 -> 4	6 -> 5
71 - C		-	-	6 -> 3
72 - V	**H Chain 104.P / L Chain 32.Y / 50.Y**	-	-	6 -> 4
77 - K	**No Interaction**	-	-	6 -> 2
78 - P		-	-	6 -> 2
79 - T		-	-	6 -> 3
80 - L		6 -> 3	-	7 -> 4
81 - Q	**L Chain 50.Y**	-	-	7 -> 5
82 - L	**No Interaction**	7 -> 2	6 -> 3	-
83 - E	**L Chain 32.Y / 92.K**	**4 -> 6**	**4 -> 6**	-

90 - Y	**L Chain 93.M**	-	7 -> 4	-
91 - P	**No Interaction**	7 -> 1	7 -> 2	-
92 - K	**L Chain 93.M**	-	7 -> 3	-
93 - K	**No Interaction**	6 -> 1	-	-
94 - K	**H Chain 58.D / 59.E / 60.S**	**2 -> 6**	-	-
96 - E	**H Chain 103.D / L Chain 93.M / 94.L**	**1 -> 6**	-	**3 -> 6**
97 - K	**H Chain 54.W / 55.W / 56.D / 58.D / 103.D**	**3 -> 6**	**3 -> 6**	**2 -> 6**
98 - R	**H Chain 103.D / L Chain 32.Y / 91.G / 92.K**	**3 -> 7**	**4 -> 7**	**5 -> 7**
99 - F	**No Interaction**	-	6 -> 5	**5 -> 6**
101 - F		7 -> 5	7 -> 5	-
103 - K		-	-	**4 -> 6**
111 - E		-	6 -> 5	**4 -> 7**
112 - F		7 -> 3	7 -> 6	-
116 - Q	**H Chain 55.W / 102.Y / 103.D**	**4 -> 6**	-	-
117 - F	**H Chain 102.Y**	-	-	7 -> 5
120 - W	**No Interaction**	7 -> 4	-	7 -> 5
121 - Y		-	6 -> 5	**4 -> 6**
122 - I		-	6 -> 4	-
132 - V		7 -> 2	-	-
134 - L		6 -> 4	-	6 -> 4
144 - T		6 -> 3	6 -> 4	-
146 - F		6 -> 3	7 -> 5	**5 -> 6**

Table S13. ESSA result comparison between the bound and unbound state of IL-1β part of IgG26:IL-1β (7CHY) at all cutoffs. Bold numbers indicate a positive change.

7CHY - Antigen&Complex AllCutOffs				
**Residue**	**Interaction**	**Rcut: 7.3 Å Z-score - Ag -> Complex**	**Rcut: 10 Å Z-score - Ag -> Complex**	**Rcut: 13 Å Z-score - Ag -> Complex**
122 - L	No Interaction	-	6 -> 4	-
123 - N	No Interaction	-	6 -> 4	-
124 - C	No Interaction	-	6 -> 4	-
125 - T	No Interaction	-	6 -> 5	-
126 - L	No Interaction	-	6 -> 5	6 -> 4
127 - R	No Interaction	-	6 -> 4	7 -> 4
132 - K	No Interaction	6 -> 2	-	-
134 - L	No Interaction	-	-	7 -> 4
135 - V	No Interaction	-	-	7 -> 3
142 - L	No Interaction	6 -> 4	-	-
145 - L	**H Chain 57.F**	-	-	6 -> 4
147 - L	**H Chain 57.F / 59.Y**	6 -> 2	7 -> 4	-
152 - M	No Interaction	-	-	6 -> 2
153 - E	No Interaction	-	-	6 -> 2
154 - Q	**H Chain 57.F**	6 -> 2	-	7 -> 3
155 - Q	No Interaction	7 -> 2	7 -> 5	7 -> 3
156 - V	No Interaction	6 -> 2	7 -> 4	7 -> 3
157 - V	No Interaction	-	7 -> 5	-
158 - F	No Interaction	7 -> 2	-	7 -> 5
176 - L	No Interaction	-	6 -> 5	-
184 - Y	No Interaction	7 -> 3	7 -> 5	-
185 - L	No Interaction	-	6 -> 5	6 -> 5
187 - C	No Interaction	-	-	6 -> 5
190 - K	No Interaction	-	-	**5 -> 6**
193 - K	No Interaction	-	-	**5 -> 6**
196 - L	No Interaction	6 -> 2	6 -> 5	-
197 - Q	No Interaction	-	-	**6 -> 7**
198 - L	No Interaction	6 -> 2	-	-
206 - Y	No Interaction	-	7 -> 3	-

207 - P		-	7 -> 3	-
215 - F		-	6 -> 5	-
217 - F		7 -> 3	7 -> 6	-
228 - F		7 -> 2	7 -> 6	6 -> 5
236 - W		7 -> 3	-	-
250 - L		6 -> 2	-	-
262 - F		6 -> 2	6 -> 5	-

Table S14. ESSA result comparison between the bound and unbound state of IL-1β part of AAL160: IL-1β (7Z4T) at all cutoffs. Bold numbers indicate a positive change.

7Z4T - Antigen&Complex AllCutOffs				
**Residue**	**Interaction**	**Rcut: 7.3 Z-score - Ag -> Complex**	**Rcut: 10 Z-score - Ag -> Complex**	**Rcut: 13 Z-score - Ag -> Complex**
6 - L	No Interaction	-	6 -> 3	-
7 - N		7 -> 1	-	-
10 - L		-	7 -> 5	6 -> 5
11 - R		-	6 ->4	7 -> 6
16 - K		6 -> 2	-	-
18 - L		-	-	6 -> 5
19 - V		-	-	6 -> 5
20 - M	**H Chain 33.W - L Chain 94.W**	-	-	**5 -> 6**
24 - Y	**H Chain 32.Y / 33.Q / 99.Y / 100.T / 101.N**	-	**4 -> 7**	**4 -> 7**
25 - E	**L Chain 91.R**	**4 ->6**	**3 -> 7**	**4 -> 6**
26 - L	**No Interaction**	-	6 -> 5	-
27 - K	**L Chain 93.N**	-	-	4 ->6
31 - L	**No Interaction**	-	6 -> 3	-
38 - Q		-	-	**5 -> 6**
39 - Q		7 -> 3	7 -> 6	-
40 - V		7 ->2	7 -> 5	7 -> 5
41 - V		6 -> 2	7 -> 4	-
42 - F		7 ->2	7 -> 5	7 -> 6
63 - K		7 ->2	-	-
68 - Y		7 -> 3	7 -> 4	**5 -> 6**
69 - L		6 -> 3	7 -> 4	6 -> 5
71 - C		-	-	6 ->4
72 - V		-	-	6 ->4
77 - K		-	-	6 -> 3
79 - T		-	-	6 -> 5
80 - L		6 -> 3	6 ->4	7 -> 5
81 - Q		-	-	7 -> 5
82 - L	**H Chain 101.N**	7 -> 2	-	-

90 - Y		6 -> 2	6 -> 3	-
101 - F		7 -> 4	7 -> 5	6 -> 5
104 - I		-	7 -> 2	-
105 - E		-	6 -> 2	-
110 - L		-	6 -> 3	-
111 - E		6 -> 2	7 -> 3	-
112 - F	**No Interaction**	6 -> 2	7 -> 5	7 -> 6
117 - F		6 -> 2	-	7 -> 5
120 - W		6 -> 2	-	7 -> 6
122 - I		-	6 -> 4	-
132 - V		6 -> 2	-	
134 - L		-	-	7 -> 5
143 - I		6 -> 1	-	-
145 - D		-	6 -> 2	-
146 - F		6 -> 2	6 -> 4	-

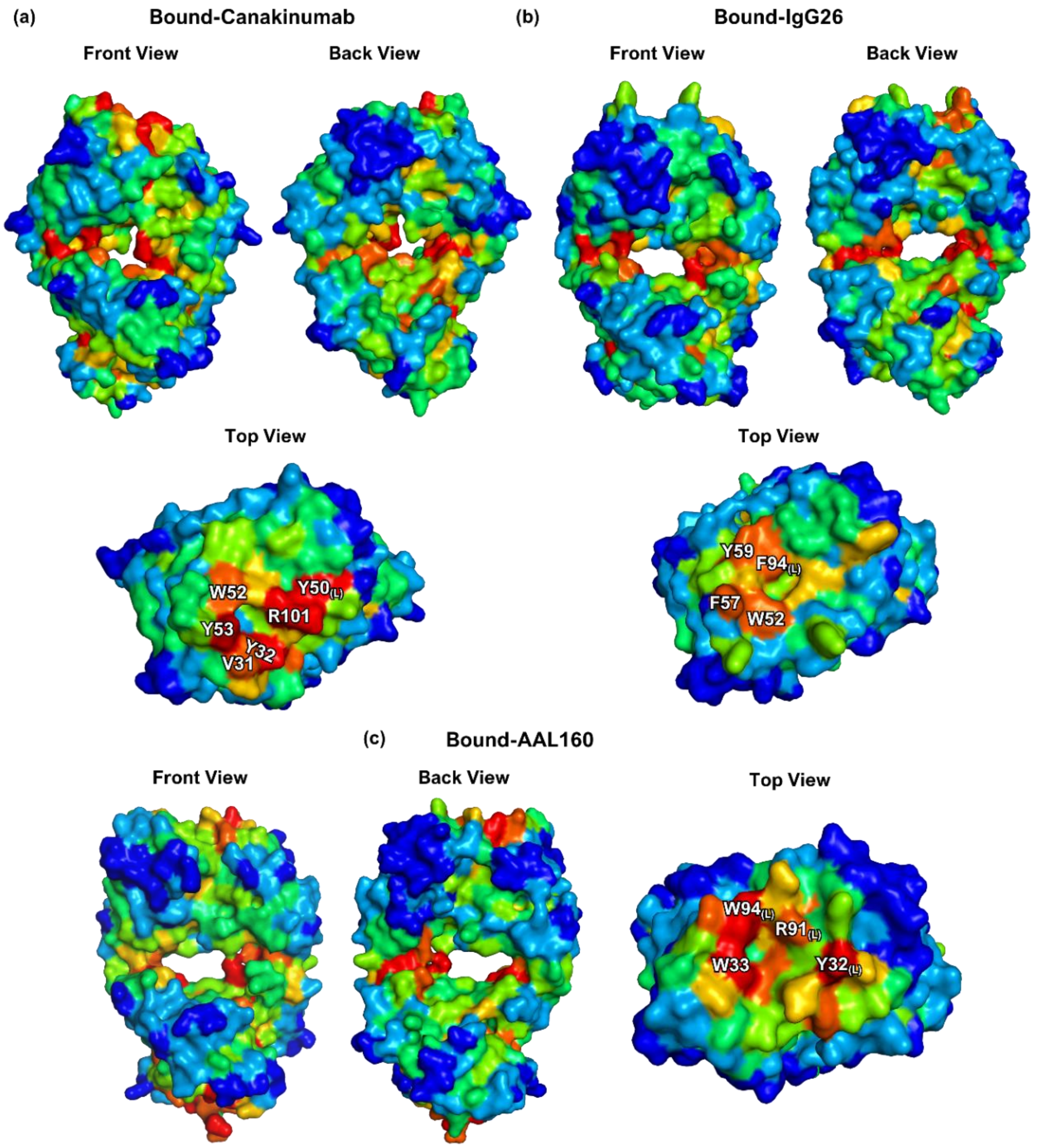

Figure S8. Combined-cutoff ESSA results for the bound Abs. In each structure, the left side is the H chain, and the right side is the L chain. The top views show the newly detected interacting residues. (a) IL-1β-bound Canakinumab, (b) IL-1β-bound IgG26, and (c) IL-1β-bound AAL160.

Table S15. ESSA results comparing the bound and unbound state of Canakinumab (4G6J) at all cutoffs. Bold numbers indicate a positive change. *CDR1, **CDR2, ***CDR3.

**4G6J - Antibody&Complex AllCutOffs**				
**Residue (H Chain)**	**Interaction**	**Rcut: 7.3 Å Zscore - Ag -> Complex**	**Rcut: 10 Å Zscore - Ag -> Complex**	**Rcut: 13 Å Zscore - Ag -> Complex**
27 - F	**No Interaction**	-	6 -> 5	-
31 - V	**Ag 31.L / 35.D / 38.Q**	**1 -> 6**	-	-
32 - Y	**Ag 35.D / 37.E / 38.Q**	**3 -> 7**	**4 -> 7**	-
36 - W	**No Interaction**	-	-	6 -> 4
47 - W		-	6 -> 5	7 -> 5
52 - W	**Ag 20.M / 21.S / 22.G / 23.P**	-	**4 -> 6**	**4 -> 6**
53 - Y	**Ag 19.V / 20.M / 27.K / 29.L / 129.N**	**1 -> 7**	**2 -> 7**	-
94 - Y	**No Interaction**	-	-	6 -> 5
95 - Y		-	-	6 -> 5
98 - R	**Ag 37.E**	-	-	6 -> 5
101 - R	**Ag 37.E / 38.Q / 39.Q / 64.E**	**1 -> 6**	**3 -> 7**	**3 -> 7**
105 - F***	**No Interaction**	-	-	7 -> 5
106 - D***		-	-	6 -> 5
107 - Y***		-	7 -> 6	7 -> 5
108 - W		-	-	7 -> 6
127 - F		-	-	7 -> 5
146 - L		-	-	6 -> 5
148 - K		-	-	7 -> 6
154 - P		**5 -> 6**	-	-
169 - H		7 -> 6	-	-
172 - P		-	**5 -> 6**	-
199 - Y		7 -> 5	7 -> 5	-
**L Chain**				
36 - Y	**No Interaction**	-	-	7 -> 5
50 - Y	**Ag 64.E**	-	**3 -> 7**	**3 -> 6**
62 - F	**No Interaction**	-	6 -> 5	-
86 - Y		-	-	7 -> 5
87 - Y		-	-	6 -> 5
89 - H***		-	-	6 -> 5
98 - F		-	-	6 -> 5
116 - F		7 -> 6	-	-
118 - F		-	-	7 -> 6
119 - P		-	-	6 -> 4
120 - P		-	-	6 -> 4
132 - V		-	-	6 -> 4
140 - Y		-	-	6 -> 5
142 - R		-	-	7 -> 6

148 - W		-	-	7 -> 5
165 - E		**6 -> 7**	-	-
179 - L		-	6 -> 5	6 -> 4
180 - T		-	-	7 -> 4
181 - L		-	6 -> 5	6 -> 2
186 - Y		-	7 -> 6	6 -> 3
192 - Y		-	-	7 -> 4
207 - K		**5 -> 6**	-	-
209 - F		6 -> 5	-	6 -> 5

Table S16. ESSA results comparing the bound and unbound state of Gevokizumab (4G6M) at all cutoffs. Bold numbers indicate a positive change. *CDR1, **CDR2, ***CDR3.

**4G6M - Antibody&Complex AllCutOffs**				
**Residue (H Chain)**	**Interaction**	**Rcut: 7.3 Å Zscore - Ag -> Complex**	**Rcut: 10 Å Zscore - Ag -> Complex**	**Rcut: 13 Å Zscore - Ag -> Complex**
4 - L	**No Interaction**	-	-	6 -> 4
24 - F		-	-	7 -> 4
27 - F		-	-	7 -> 4
38 - W		-	-	6 -> 5
40 - R		-	6 -> 5	-
49 - W		-	6 -> 5	-
54 - W	**Ag 97.K**	-	**3 -> 6**	**4 -> 7**
55 - W	**Ag 97.K / 115.A / 116.Q**	-	-	**2 -> 6**
58 - D	**Ag 94.K / 97.K**	**1 -> 6**	-	-
96 - F	**No Interaction**	-	-	6 -> 5
99 - R		-	-	7 -> 5
102 - Y	**Ag 116.Q / 117.F**	**1 -> 6**	**2 -> 6**	**3 -> 6**
103 - D	**Ag 96.E / 97.K / 98.R / 116.Q**	-	**2 -> 6**	-
106 - W***	**No Interaction**	-	**5 -> 7**	**5 -> 6**
107 - F***		-	-	6 -> 5
108 - V***		-	-	7 -> 6
109 - D***		-	-	7 -> 5
110 - W		-	6 -> 5	-
115 - L		6 -> 5	**5 -> 6**	-
145 - L		-	-	6 -> 5
154 - P		6 -> 5	-	-
171 - H		6 -> 5	-	-
201 - Y		-	6 -> 5	6 -> 5
**L Chain**				
27 - Q	**Ag 86.D**	**1 -> 6**	-	-
30 - S*	**No Interaction**	**1 -> 6**	-	-
32 - Y	**Ag 72.V / 83.E / 98.R**	**2 -> 7**	**2 -> 7**	**2 -> 6**
36 - Y	**No Interaction**	-	-	6 -> 5
50 - Y	**Ag 72.V / 73.L / 74.K / 81.Q**	**1 -> 7**	**2 -> 6**	-
53 - K	**Ag 75.D**	**1 -> 6**	-	-
62 - F	**No Interaction**	-	6 -> 5	-
92 - K	**Ag 83.E / 84.S / 98.R**	**1 -> 7**	-	-
93 - M	**Ag 89.N / 90.Y / 92.K / 96.D**	**1 -> 6**	-	-
118 - F	**No Interaction**	7 -> 6	-	-
140 - Y		-	-	6 -> 5

148 - W		6 -> 5	-	-
166 - Q		-	**6 -> 7**	-
186 - Y		7 -> 5	-	-
192 - Y		7 -> 6	-	6 -> 4
209 - F		7 -> 5	-	-
211 - R		-	6 -> 5	-

Table S17. ESSA results comparing the bound and unbound state of IgG26 (7CHY) at all cutoffs. Bold numbers indicate a positive change. *CDR1, **CDR2, ***CDR3.

**7CHY - Antibody&Complex AllCutOffs**				
**Residue (H Chain)**	**Interaction**	**Rcut: 7.3 Å Zscore - Ag -> Complex**	**Rcut: 10 Å Zscore - Ag -> Complex**	**Rcut: 13 Å Zscore - Ag -> Complex**
11 - L	No Interaction	**6 -> 7**	-	-
32 - Y*		-	-	6 -> 3
35 - H*		-	-	7 -> 4
36 - W		-	-	7 -> 4
47 - W		-	-	7 -> 4
52 - W**	**Ag 244.E / 245.N**	-	**4 -> 6**	6 -> 5
57 - F**	**Ag 145.L / 147.L / 151.D / 154.Q**	-	**1 -> 6**	-
59 - Y**	**Ag 146.H / 147.L / 148.Q**	-	**3 -> 6**	-
94 - Y	No Interaction	-	6 -> 5	6 -> 4
98 - R		-	-	7 -> 3
99 - F***	**Ag 243.A**	-	6 -> 5	7 -> 4
105 - Y***	No Interaction	-	-	7 -> 3
106 - I***		-	-	6 -> 3
107 - M***		-	-	6 -> 3
108 - D***		-	-	7 -> 3
109 - Y***		-	-	6 -> 3
110 - W		-	6 -> 5	7 -> 5
115 - L		-	6 -> 5	-
129 - F		-	-	6 -> 4
136 - K		-	-	6 -> 4
145 - L		-	-	6 -> 5
150 - K		-	-	6 -> 5
153 - F		7 -> 6	7 -> 6	7 -> 6
155 - E		-	7 -> 6	-
156 - P		-	-	**5 -> 6**
183 - Y			7 -> 6	-
191 - V		-	-	7 -> 4
192 - P		-	-	6 -> 3
196 - L		-	-	6 -> 3
201 - Y		-	-	7 -> 4
**L Chain**				
35 - W	No Interaction	-	-	6 -> 3
36 - Y		-	-	6 -> 4
49 - Y		-	-	6 -> 3
55 - Y**		-	-	6 -> 3

62 - F		-	-	6 -> 3
83 -F		7 -> 6	-	-
86 - Y		-	6 -> 5	7 -> 4
91 - Y***		-	-	7 -> 5
94 - F***	**Ag 146.H / 244.E**	-	**4 -> 6**	-
105 - E		**5 -> 6**	-	**5 -> 6**
140 - Y		**6 -> 7**	-	6 -> 5
148 - W		-	-	6 -> 4
179 - L		-	6 -> 5	6 -> 3
180 - T	**No Interaction**	-	-	6 -> 3
181 - L		-	-	7 -> 2
186 - Y		-	-	6 -> 3
192 - Y		-	-	7 -> 3
195 - E		-	-	6 -> 3

Table S18. ESSA results comparing the bound and unbound states of AAL160 (7Z4T) at all cutoffs. Bold numbers indicate a positive change. *CDR1, **CDR2, ***CDR3.

7Z4T - Antibody&Complex AllCutOffs				
**Residue (H Chain)**	**Interaction**	**Rcut: 7.3 Å Zscore - Ag -> Complex**	**Rcut: 10 Å Zscore - Ag -> Complex**	**Rcut: 13 Å Zscore - Ag -> Complex**
33 - W	**Ag 20.M / 23.P / 24.Y**	-	**3 -> 6**	**4 -> 7**
36 - W	**No Interaction**	-	-	6 -> 4
47 - W		-	-	6 -> 4
59 - R**		-	**2 -> 6**	**3 -> 6**
83 - W		-	-	6 -> 4
94 - Y		-	-	6 -> 5
95 - Y		-	-	6 -> 5
102 - W***		-	**3 -> 6**	-
105 - F***		-	-	6 -> 5
108 - W		-	6 -> 5	7 -> 5
115 - T		-	**5 -> 6**	-
127 - F		-	-	6 -> 5
131 - P		-	-	6 -> 4
132 - S		-	-	6 -> 4
134 - K		-	-	6 -> 4
146 - L		-	**5 -> 6**	-
148 - K		-	-	6 -> 5
152 - P		-	**5 -> 6**	-
169 - H		-	-	6 -> 5
199 - Y		-	-	6 -> 4
218 - P		-	-	7 -> 4
**L Chain**				
32 - Y	**Ag 74.K**	-	**2 -> 7**	**2 -> 7**
36 - Y	**No Interaction**	-	-	6 -> 5
91 - R	**Ag 21.S / 22.G / 23.P / 25.E**	-	**2 -> 6**	**3 -> 6**
94 - W	**Ag 20.M / 21.S / 22.G / 23.P**	-	**3 -> 7**	**4 -> 7**
103 - K	**No Interaction**	-	-	**5 -> 6**
106 - I		**5 -> 6**	-	-
117 - I		**5 -> 6**	-	-
119 - P		-	-	7 -> 5
120 - P		-	-	6 -> 4
122 - D		-	-	6 -> 4
135 - L		-	**5 -> 6**	-
140 - Y		-	-	6 -> 5
165 - E		**5 -> 6**	-	-
207 - K		-	-	6 -> 5
209 - F		-	-	7 -> 5
211 - R		-	-	6 -> 4

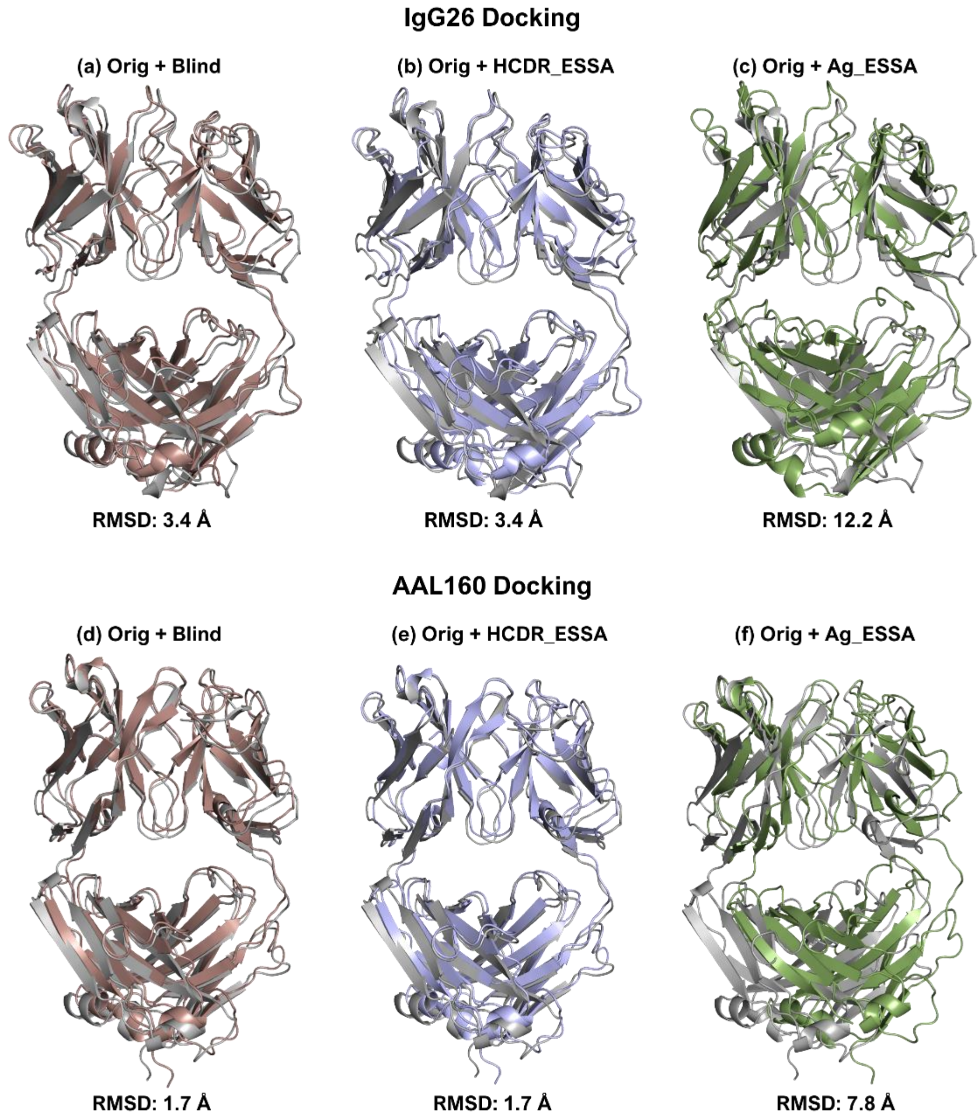

Figure S9. The best docking poses for IgG26 (a, b, and c) and AAL160 (d, e, and f). RMSD values are calculated between the original structure and each docking pose.

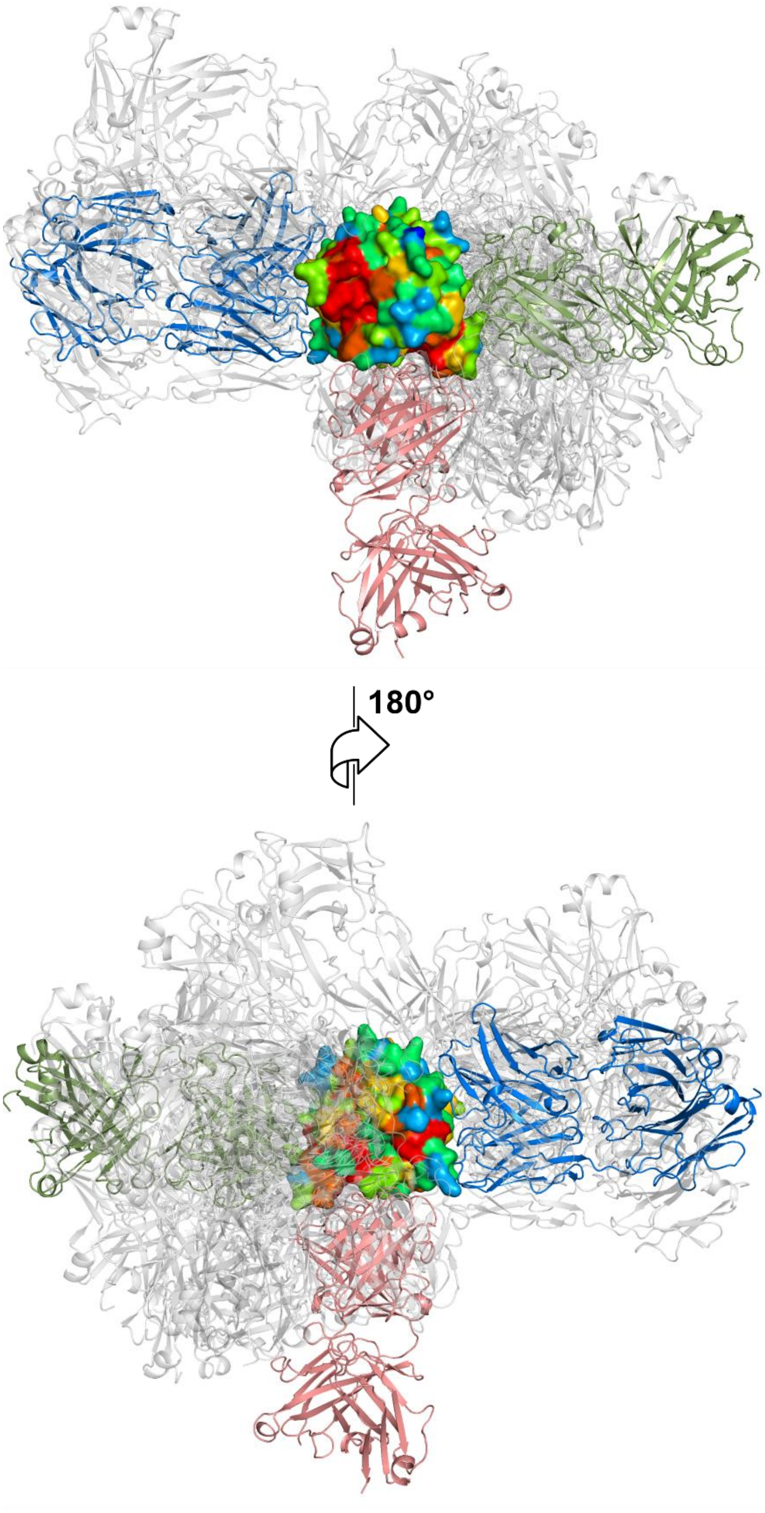

Figure S10. Gevokizumab swapped docking poses. All poses were shown as transparent light gray cartoons, whereas the best pose, which was selected based on the number of HCDR and LCDR contacts with the antigen, was green.

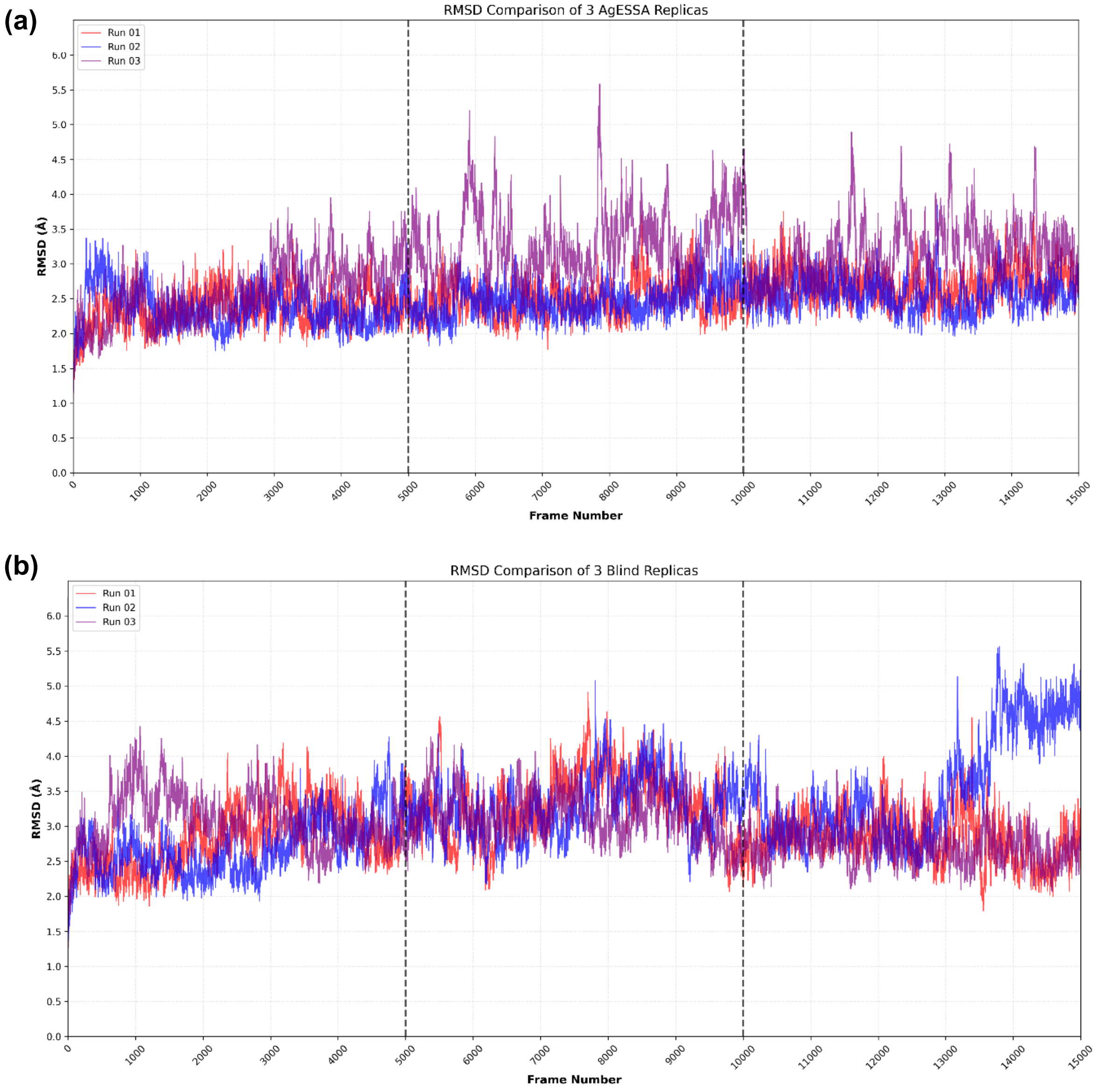

Figure S11. The RMSD profiles of the Gevokizumab-bound IL-1β Ag_ESSA and Blind docking poses (red-line: Run01, blue-line: Run02, purple-line: Run03) (a) RMSD profiles of Ag_ESSA runs. (b) RMSD profiles of Blind runs. Each run is 300 ns in length.

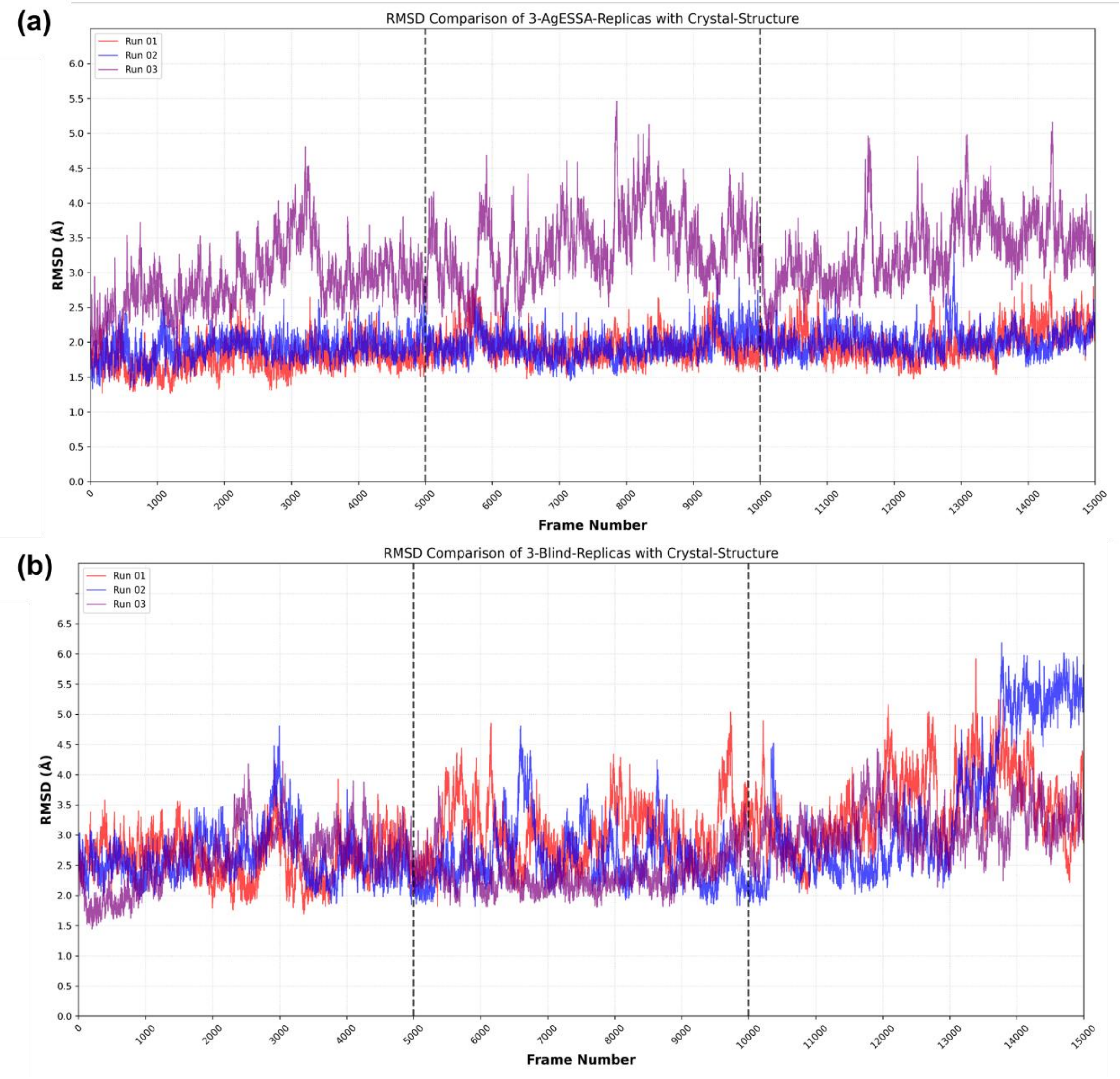

Figure S12. The RMSD profile comparison of the Ag_ESSA and Blind frames relative to the crystal structure. (a) Line plot representation of Ag_ESSA RMSD comparisons. (b) Line plot representation of Blind RMSD comparisons. (red-line: Run01, blue-line: Run02, purple-line: Run03)

Table S19. Formed H-Bonds between Ag and Ab above or equal to the 50% threshold in Ag_ESSA three replica MD runs. The number of frames used in the calculations are 5,000. AbL: Light chain of the antibody; AbH: Heavy chain of the antibody; Ag: antigen.

	Formed Original H-Bonds	# of Frames	Percentage (%)
Ag_ESSA-Run01	Ag:E83-OE2 – AbL:Y32-OH	4969	99.38
	Ag:E83-OE1 – AbL:K92-NZ	4886	97.72
	Ag:S84-O – AbL:K92-NZ	4730	94.60
	Ag:Q116-OE1 – AbH:D103-N	4518	90.36
	Ag:K97-NZ – AbH:D58-OD2	4092	81.84
	Ag:K94-NZ – AbH:D58-OD1	3539	70.78
	Ag:K97-NZ – AbH:D56-OD2	3019	60.38
	Ag:Q116-NE2 – AbH:D103-O	2768	55.36
Ag_ESSA-Run02	Ag:E83-OE2 – AbL:Y32-OH	4987	99.74
	Ag:E83-OE1 – AbL:K92-NZ	4874	97.48
	Ag:S84-O – AbL:K92-NZ	4727	94.54
	Ag:Q116-OE1 – AbH:D103-N	3946	78.92
	Ag:Q116-NE2 – AbH:D103-O	3290	65.80
	Ag:K97-NZ – AbH:D56-OD2	3234	64.68
	Ag:K94-NZ – AbH:D58-OD2	2798	55.96
Ag_ESSA-Run03	Ag:K97-NZ – AbH:D56-OD2	3723	74.46

Table S20. Formed H-Bonds between Ag and Ab above or equal to the 50% threshold in Blind three replica MD runs. The number of frames used in the calculations are 5,000. (AbH: Heavy chain of the antibody; Ag: antigen; x: not available.)

	Formed Original H-Bonds	# of Frames	Percentage (%)
**Blind-Run01**	Ag:K97-NZ – AbH:D56-OD2	3533	70.66
**Blind-Run02**	x	x	x
**Blind-Run03**	Ag:K97-NZ – AbH:D56-OD2	3936	78.72
	Ag:K94-NZ – AbH:D58-OD1	2578	51.56
	Ag:K94-NZ – AbH:D58-OD2	2500	50.00

Table S21. Selected essential residues for ESSA-guided docking.

	Blind	HCDR_ESSA	Ag_ESSA
**Structures**	**Forced Residues**		
**4G6J (Canakinumab:IL-1β)**	-	R98, F105, D106, Y107	L31, Q38, Q39
**4G6M (Gevokizumab:IL-1β)**	-	F107, V108, D109	V72, Q81, Y90, K92, F117
**7CHY (IgG26:IL-1β)**	-	W52, F99, Y105, I106, M107, D108, Y109	L145, L147, Q154
**7Z4T (AAL160:IL-1β)**	-	W36, Y99, F105, W108	Y24, K74, L82

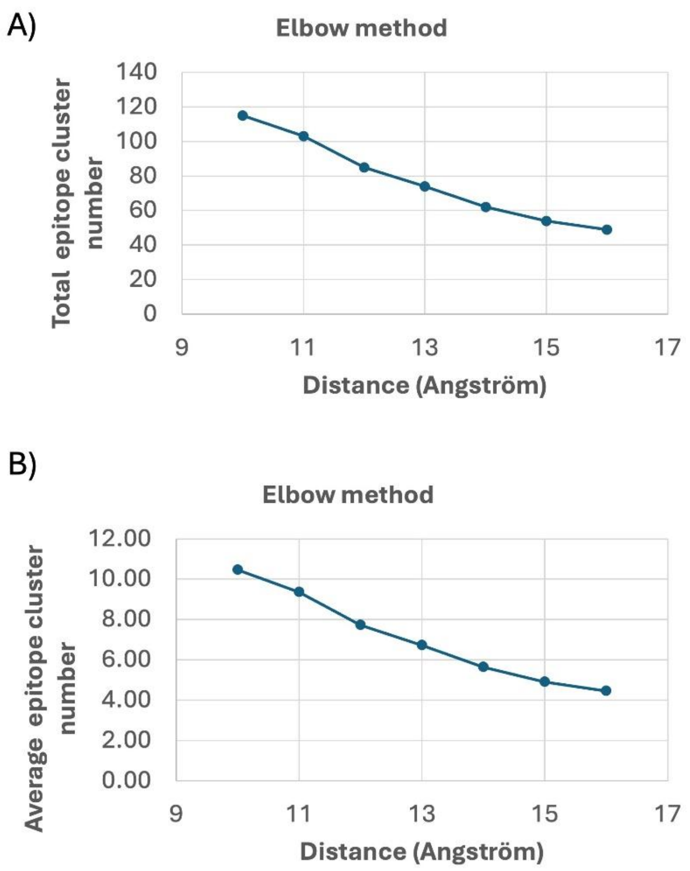

Figure S13. The Elbow Method. (A) Total number of formed epitope clusters using different distance threshold values, and (B) Average number of formed epitope clusters using different distance threshold values.

Table S22. Canakinumab:Ag (4G6J) interactions in the best Blind docking pose.

4G6J Blind		
**Ag**	**Interaction Type**	**Ab - H Chain**
Ser21	Hydrogen Bonds	Asn57
Glu25	Hydrogen Bonds	Asn57
Lys27	Hydrogen Bonds	Asp54
Gln34	Hydrogen Bonds	Gln1
Asp35	Hydrogen Bonds	Tyr32
Asp35	Hydrogen Bonds	Thr28
Glu37	Hydrogen Bonds	Arg101
Gln38	Hydrogen Bonds	Arg101
Gln38	Hydrogen Bonds	Asp99
Gln39	Hydrogen Bonds	Arg101
Glu64	Hydrogen Bonds	Arg101
Lys65	Hydrogen Bonds	Thr102
Asn129	Hydrogen Bonds	Tyr53
Asn129	Hydrogen Bonds	Asp54
Val19	Non-Bonded Contacts	Tyr53
Met20	Non-Bonded Contacts	Trp52
Met20	Non-Bonded Contacts	Tyr53
Met20	Non-Bonded Contacts	Thr102
Met20	Non-Bonded Contacts	Arg101
Ser21	Non-Bonded Contacts	Trp52
Ser21	Non-Bonded Contacts	Tyr53
Ser21	Non-Bonded Contacts	Asn57
Gly22	Non-Bonded Contacts	Trp52
Gly22	Non-Bonded Contacts	Asn57
Pro23	Non-Bonded Contacts	Trp52
Glu25	Non-Bonded Contacts	Asn57
Lys27	Non-Bonded Contacts	Tyr53
Lys27	Non-Bonded Contacts	Asp54
Lys27	Non-Bonded Contacts	Asp56
Leu29	Non-Bonded Contacts	Tyr53
Leu31	Non-Bonded Contacts	Val31
Gln34	Non-Bonded Contacts	Gln1
Asp35	Non-Bonded Contacts	Tyr32
Asp35	Non-Bonded Contacts	Val31
Asp35	Non-Bonded Contacts	Thr28
Glu37	Non-Bonded Contacts	Leu100
Glu37	Non-Bonded Contacts	Arg101
Glu37	Non-Bonded Contacts	Arg98
Gln38	Non-Bonded Contacts	Arg101
Gln38	Non-Bonded Contacts	Val31
Gln38	Non-Bonded Contacts	Tyr32
Gln38	Non-Bonded Contacts	Leu100
Gln38	Non-Bonded Contacts	Asp99
Gln39	Non-Bonded Contacts	Arg101
Val41	Non-Bonded Contacts	Arg101
Glu64	Non-Bonded Contacts	Arg101
Lys65	Non-Bonded Contacts	Thr102
Asn129	Non-Bonded Contacts	Tyr53

Asn129	Non-Bonded Contacts	Asp54
Lys27	Salt Bridges	Asp54
Lys27	Salt Bridges	Asp56
Glu37	Salt Bridges	Arg98
Glu64	Salt Bridges	Arg101
		**Ab - L Chain**
Glu64	Hydrogen Bonds	Ser32
Glu64	Hydrogen Bonds	Tyr50
Lys65	Hydrogen Bonds	Ser91
Lys65	Hydrogen Bonds	Ser92
Asn66	Hydrogen Bonds	Ser92
Asn66	Hydrogen Bonds	Ser93
Asp86	Hydrogen Bonds	Ser28
Pro23	Non-Bonded Contacts	Leu94
Glu64	Non-Bonded Contacts	Ser32
Glu64	Non-Bonded Contacts	Tyr50
Lys65	Non-Bonded Contacts	Ser92
Lys65	Non-Bonded Contacts	Ser91
Lys65	Non-Bonded Contacts	Phe96
Asn66	Non-Bonded Contacts	Ser92
Asn66	Non-Bonded Contacts	Ser93
Asp86	Non-Bonded Contacts	Ser28
Pro87	Non-Bonded Contacts	Ser28
Pro87	Non-Bonded Contacts	Gly30
Lys88	Non-Bonded Contacts	Ser28

Table S23. Canakinumab:Ag (4G6J) interactions in the best HCDR_ESSA and Ag_ESSA docking poses.

4G6J HCDR_ESSA & Ag_ESSA		
**Ag**	**Interaction Type**	**Ab - H Chain**
Ser21	Hydrogen Bonds	Asn57
Glu25	Hydrogen Bonds	Asn57
Lys27	Hydrogen Bonds	Asp54
Lys27	Hydrogen Bonds	Asp56
Gln34	Hydrogen Bonds	Gln1
Asp35	Hydrogen Bonds	Tyr32
Asp35	Hydrogen Bonds	Thr28
Glu37	Hydrogen Bonds	Arg101
Gln38	Hydrogen Bonds	Arg101
Gln38	Hydrogen Bonds	Asp99
Glu64	Hydrogen Bonds	Arg101
Lys65	Hydrogen Bonds	Thr102
Asn129	Hydrogen Bonds	Tyr53
Asn129	Hydrogen Bonds	Asp54
Val19	Non-Bonded Contacts	Tyr53
Met20	Non-Bonded Contacts	Trp52
Met20	Non-Bonded Contacts	Tyr53
Met20	Non-Bonded Contacts	Thr102
Ser21	Non-Bonded Contacts	Trp52
Ser21	Non-Bonded Contacts	Asn57
Ser21	Non-Bonded Contacts	Asp54
Gly22	Non-Bonded Contacts	Trp52
Gly22	Non-Bonded Contacts	Asn57
Pro23	Non-Bonded Contacts	Asn57
Glu25	Non-Bonded Contacts	Asn57
Lys27	Non-Bonded Contacts	Tyr53
Lys27	Non-Bonded Contacts	Asp56
Lys27	Non-Bonded Contacts	Asp54
Leu29	Non-Bonded Contacts	Tyr53
Leu31	Non-Bonded Contacts	Val31
Gln34	Non-Bonded Contacts	Gln1
Asp35	Non-Bonded Contacts	Tyr32
Asp35	Non-Bonded Contacts	Thr28
Asp35	Non-Bonded Contacts	Val31
Glu37	Non-Bonded Contacts	Leu100
Glu37	Non-Bonded Contacts	Arg101
Glu37	Non-Bonded Contacts	Arg98
Gln38	Non-Bonded Contacts	Arg101
Gln38	Non-Bonded Contacts	Tyr32
Gln38	Non-Bonded Contacts	Asp99
Gln38	Non-Bonded Contacts	Leu100
Gln39	Non-Bonded Contacts	Arg101
Val41	Non-Bonded Contacts	Arg101
Glu64	Non-Bonded Contacts	Arg101
Lys65	Non-Bonded Contacts	Thr102
Asn129	Non-Bonded Contacts	Tyr53

Asn129	Non-Bonded Contacts	Asp54
Lys27	Salt Bridges	Asp54
Lys27	Salt Bridges	Asp56
Glu37	Salt Bridges	Arg98
Glu64	Salt Bridges	Arg101
		**Ab - L Chain**
Glu64	Hydrogen Bonds	Ser32
Lys65	Hydrogen Bonds	Ser91
Lys65	Hydrogen Bonds	Ser92
Glu64	Non-Bonded Contacts	Ser32
Glu64	Non-Bonded Contacts	Tyr50
Lys65	Non-Bonded Contacts	Ser91
Lys65	Non-Bonded Contacts	Ser92

Table S24. Gevokizumab:Ag (4G6M) interactions in the best Blind docking pose.

4G6M Blind		
**Ag**	**Interaction Type**	**Ab - H Chain**
Glu50	Hydrogen Bonds	Trp55
Lys94	Hydrogen Bonds	Asp58
Lys94	Hydrogen Bonds	Glu59
Lys97	Hydrogen Bonds	Asp56
Lys97	Hydrogen Bonds	Asp58
Gln116	Hydrogen Bonds	Asp103
Gln48	Non-Bonded Contacts	Asp58
Glu50	Non-Bonded Contacts	Trp55
Leu73	Non-Bonded Contacts	Asp103
Leu73	Non-Bonded Contacts	Pro104
Leu73	Non-Bonded Contacts	Trp106
Lys94	Non-Bonded Contacts	Asp58
Lys94	Non-Bonded Contacts	Glu59
Met95	Non-Bonded Contacts	Asp58
Lys97	Non-Bonded Contacts	Trp55
Lys97	Non-Bonded Contacts	Asp56
Lys97	Non-Bonded Contacts	Trp54
Lys97	Non-Bonded Contacts	Asp58
Arg98	Non-Bonded Contacts	Asp103
Ala115	Non-Bonded Contacts	Trp55
Gln116	Non-Bonded Contacts	Gly33
Gln116	Non-Bonded Contacts	Tyr102
Gln116	Non-Bonded Contacts	Asp103
Phe117	Non-Bonded Contacts	Tyr102
Phe117	Non-Bonded Contacts	Asp103
Pro118	Non-Bonded Contacts	Ser32
Pro118	Non-Bonded Contacts	Arg101
Lys94	Salt Bridges	Asp58
Lys94	Salt Bridges	Glu59
Lys97	Salt Bridges	Asp56
Lys97	Salt Bridges	Asp58
		**Ab - L Chain**
Leu73	Hydrogen Bonds	Tyr32
Asp75	Hydrogen Bonds	Tyr50
Arg98	Hydrogen Bonds	Lys92
Leu73	Non-Bonded Contacts	Tyr32
Lys74	Non-Bonded Contacts	Tyr50
Lys74	Non-Bonded Contacts	Tyr32
Asp75	Non-Bonded Contacts	Tyr50
Asp75	Non-Bonded Contacts	Asn31
Asp76	Non-Bonded Contacts	Lys53
Arg98	Non-Bonded Contacts	Lys92
Asp76	Salt Bridges	Lys53

Table S25. Gevokizumab:Ag (4G6M) interactions in the best HCDR_ESSA docking pose.

4G6M HCDR_ESSA		
**Ag**	**Interaction Type**	**Ab - H Chain**
Leu73	Hydrogen Bonds	Asp103
Lys94	Hydrogen Bonds	Glu59
Gln116	Hydrogen Bonds	Asp103
Val72	Non-Bonded Contacts	Asp103
Val72	Non-Bonded Contacts	Pro104
Leu73	Non-Bonded Contacts	Tyr102
Leu73	Non-Bonded Contacts	Trp106
Lys94	Non-Bonded Contacts	Glu59
Lys94	Non-Bonded Contacts	Asp58
Lys97	Non-Bonded Contacts	Trp54
Lys97	Non-Bonded Contacts	Trp55
Lys97	Non-Bonded Contacts	Asp56
Lys97	Non-Bonded Contacts	Asp58
Arg98	Non-Bonded Contacts	Asp103
Ala115	Non-Bonded Contacts	Trp55
Gln116	Non-Bonded Contacts	Trp55
Gln116	Non-Bonded Contacts	Gly33
Gln116	Non-Bonded Contacts	Tyr102
Gln116	Non-Bonded Contacts	Asp103
Gln116	Non-Bonded Contacts	Trp54
Phe117	Non-Bonded Contacts	Tyr102
Lys94	Salt Bridges	Asp58
Lys94	Salt Bridges	Glu59
Lys97	Salt Bridges	Asp56
Lys97	Salt Bridges	Asp58
Arg98	Salt Bridges	Asp103
		**Ab - L Chain**
Lys74	Hydrogen Bonds	Tyr32
Asp75	Hydrogen Bonds	Lys53
Asp76	Hydrogen Bonds	Lys53
Arg98	Hydrogen Bonds	Lys92
Val72	Non-Bonded Contacts	Tyr32
Leu73	Non-Bonded Contacts	Tyr32
Lys74	Non-Bonded Contacts	Tyr50
Lys74	Non-Bonded Contacts	Tyr32
Asp75	Non-Bonded Contacts	Tyr50
Asp75	Non-Bonded Contacts	Lys53
Asp76	Non-Bonded Contacts	Lys53
Asp76	Non-Bonded Contacts	Tyr49
Gln81	Non-Bonded Contacts	Tyr32
Arg98	Non-Bonded Contacts	Lys92
Asp75	Salt Bridges	Lys53
Asp76	Salt Bridges	Lys53

Table S26. Gevokizumab:Ag (4G6M) interactions in the best Ag_ESSA docking pose.

4G6M Ag_ESSA		
**Ag**	**Interaction Type**	**Ab - H Chain**
Leu73	Hydrogen Bonds	Asp103
Lys94	Hydrogen Bonds	Glu59
Gln116	Hydrogen Bonds	Tyr102
Val72	Non-Bonded Contacts	Asp103
Leu73	Non-Bonded Contacts	Asp103
Leu73	Non-Bonded Contacts	Pro104
Lys94	Non-Bonded Contacts	Glu59
Lys94	Non-Bonded Contacts	Asp58
Lys97	Non-Bonded Contacts	Trp54
Lys97	Non-Bonded Contacts	Trp55
Lys97	Non-Bonded Contacts	Asp56
Lys97	Non-Bonded Contacts	Asp58
Arg98	Non-Bonded Contacts	Asp103
Gln116	Non-Bonded Contacts	Tyr102
Gln116	Non-Bonded Contacts	Trp54
Gln116	Non-Bonded Contacts	Asp103
Phe117	Non-Bonded Contacts	Tyr102
Lys94	Salt Bridges	Asp58
Lys94	Salt Bridges	Glu59
Lys97	Salt Bridges	Asp56
Lys97	Salt Bridges	Asp58
Arg98	Salt Bridges	Asp103
		**Ab - L Chain**
Lys74	Hydrogen Bonds	Tyr32
Asp76	Hydrogen Bonds	Lys53
Arg98	Hydrogen Bonds	Lys92
Val72	Non-Bonded Contacts	Tyr32
Lys74	Non-Bonded Contacts	Tyr50
Lys74	Non-Bonded Contacts	Tyr32
Asp75	Non-Bonded Contacts	Tyr50
Asp75	Non-Bonded Contacts	Lys53
Asp76	Non-Bonded Contacts	Lys53
Gln81	Non-Bonded Contacts	Tyr32
Glu83	Non-Bonded Contacts	Lys92
Glu96	Non-Bonded Contacts	Leu94
Arg98	Non-Bonded Contacts	Lys92
Arg98	Non-Bonded Contacts	Gly91
Asp75	Salt Bridges	Lys53
Asp76	Salt Bridges	Lys53

Table S27. IgG26:Ag (7CHY) interactions in the best Blind docking pose.

7CHY Blind		
**Ag**	**Interaction Type**	**Ab - H Chain**
Met136	Hydrogen Bonds	Tyr54
His146	Hydrogen Bonds	Arg50
Leu147	Hydrogen Bonds	Tyr59
Gln148	Hydrogen Bonds	Thr58
Gln148	Hydrogen Bonds	Tyr60
Asp151	Hydrogen Bonds	Thr58
Glu244	Hydrogen Bonds	Arg50
Glu244	Hydrogen Bonds	Trp52
Met136	Non-Bonded Contacts	Tyr54
Ser137	Non-Bonded Contacts	Tyr54
Gly138	Non-Bonded Contacts	Tyr54
Leu145	Non-Bonded Contacts	Trp52
Leu145	Non-Bonded Contacts	Phe57
His146	Non-Bonded Contacts	Arg50
His146	Non-Bonded Contacts	Tyr59
Leu147	Non-Bonded Contacts	Tyr59
Leu147	Non-Bonded Contacts	Phe57
Gln148	Non-Bonded Contacts	Tyr59
Gln148	Non-Bonded Contacts	Thr58
Gln148	Non-Bonded Contacts	Tyr60
Asp151	Non-Bonded Contacts	Phe57
Asp151	Non-Bonded Contacts	Thr58
Gln154	Non-Bonded Contacts	Phe57
Ala243	Non-Bonded Contacts	Gly101
Ala243	Non-Bonded Contacts	Ile106
Glu244	Non-Bonded Contacts	Phe99
Glu244	Non-Bonded Contacts	Gly101
Glu244	Non-Bonded Contacts	Trp52
Glu244	Non-Bonded Contacts	Arg50
Asn245	Non-Bonded Contacts	Trp52
Met246	Non-Bonded Contacts	Gly101
Met246	Non-Bonded Contacts	Tyr102
Pro247	Non-Bonded Contacts	Tyr102
Glu244	Salt Bridges	Arg50
		**Ab - L Chain**
Gln130	Hydrogen Bonds	Asp28
Gln131	Hydrogen Bonds	Asn93
His146	Hydrogen Bonds	Ser92
Gln242	Hydrogen Bonds	Trp31
Gln242	Hydrogen Bonds	Ser30
Gly256	Hydrogen Bonds	Trp31
Gln257	Hydrogen Bonds	Thr51
Gln257	Hydrogen Bonds	Ser50
Gln130	Non-Bonded Contacts	Asp28
Gln131	Non-Bonded Contacts	Asn93
His146	Non-Bonded Contacts	Phe94
His146	Non-Bonded Contacts	Ser92
His146	Non-Bonded Contacts	Asn93

Gln242	Non-Bonded Contacts	Tyr91
Gln242	Non-Bonded Contacts	Ser92
Gln242	Non-Bonded Contacts	Trp31
Gln242	Non-Bonded Contacts	Gly32
Gln242	Non-Bonded Contacts	Ser50
Gln242	Non-Bonded Contacts	Val29
Gln242	Non-Bonded Contacts	Ser30
Ala243	Non-Bonded Contacts	Tyr91
Glu244	Non-Bonded Contacts	Phe94
Gly256	Non-Bonded Contacts	Trp31
Gln257	Non-Bonded Contacts	Trp31
Gln257	Non-Bonded Contacts	Ser50
Gln257	Non-Bonded Contacts	Thr51

Table S28. IgG26:Ag (7CHY) interactions in the best HCDR_ESSA docking pose.

7CHY HCDR_ESSA		
**Ag**	**Interaction Type**	**Ab - H Chain**
Met136	Hydrogen Bonds	Tyr54
His146	Hydrogen Bonds	Arg50
Leu147	Hydrogen Bonds	Tyr59
Gln148	Hydrogen Bonds	Tyr60
Asp151	Hydrogen Bonds	Phe57
Asp151	Hydrogen Bonds	Thr58
Glu244	Hydrogen Bonds	Trp52
Glu244	Hydrogen Bonds	Arg50
Met136	Non-Bonded Contacts	Tyr54
Ser137	Non-Bonded Contacts	Tyr54
Gly138	Non-Bonded Contacts	Tyr54
Leu145	Non-Bonded Contacts	Trp52
Leu145	Non-Bonded Contacts	Phe57
His146	Non-Bonded Contacts	Arg50
His146	Non-Bonded Contacts	Tyr59
Leu147	Non-Bonded Contacts	Tyr59
Leu147	Non-Bonded Contacts	Phe57
Gln148	Non-Bonded Contacts	Tyr59
Gln148	Non-Bonded Contacts	Thr58
Gln148	Non-Bonded Contacts	Tyr60
Asp151	Non-Bonded Contacts	Phe57
Asp151	Non-Bonded Contacts	Thr58
Gln154	Non-Bonded Contacts	Phe57
Ala243	Non-Bonded Contacts	Phe99
Ala243	Non-Bonded Contacts	Gly101
Ala243	Non-Bonded Contacts	Ile106
Glu244	Non-Bonded Contacts	Phe99
Glu244	Non-Bonded Contacts	Trp52
Glu244	Non-Bonded Contacts	Arg50
Asn245	Non-Bonded Contacts	Trp52
Met246	Non-Bonded Contacts	Gly101
Met246	Non-Bonded Contacts	Tyr102
Pro247	Non-Bonded Contacts	Tyr102
Glu244	Salt Bridges	Arg50
		**Ab - L Chain**
Gln130	Hydrogen Bonds	Asp28
Gln242	Hydrogen Bonds	Trp31
Gln242	Hydrogen Bonds	Ser30
Gly256	Hydrogen Bonds	Trp31
Gln257	Hydrogen Bonds	Thr51
Gln257	Hydrogen Bonds	Ser50
Gln130	Non-Bonded Contacts	Asn93
Gln130	Non-Bonded Contacts	Ser92
Gln130	Non-Bonded Contacts	Asp28
Gln130	Non-Bonded Contacts	Ser30
Gln131	Non-Bonded Contacts	Asn93
His146	Non-Bonded Contacts	Phe94
His146	Non-Bonded Contacts	Ser92

His146	Non-Bonded Contacts	Asn93
Gln242	Non-Bonded Contacts	Tyr91
Gln242	Non-Bonded Contacts	Ser92
Gln242	Non-Bonded Contacts	Gly32
Gln242	Non-Bonded Contacts	Ser50
Gln242	Non-Bonded Contacts	Trp31
Gln242	Non-Bonded Contacts	Ser30
Gln242	Non-Bonded Contacts	Val29
Ala243	Non-Bonded Contacts	Tyr91
Glu244	Non-Bonded Contacts	Phe94
Gly256	Non-Bonded Contacts	Trp31
Gln257	Non-Bonded Contacts	Trp31
Gln257	Non-Bonded Contacts	Ser50
Gln257	Non-Bonded Contacts	Thr51
Ile259	Non-Bonded Contacts	Ser30

Table S29. IgG26:Ag (7CHY) interactions in the best Ag_ESSA docking pose.

7CHY Ag_ESSA		
**Ag**	**Interaction Type**	**Ab - H Chain**
Gly138	Hydrogen Bonds	Tyr54
His146	Hydrogen Bonds	Arg50
Gln148	Hydrogen Bonds	Lys65
Asp151	Hydrogen Bonds	Phe57
Asp151	Hydrogen Bonds	Thr58
Gln154	Hydrogen Bonds	Gly55
Glu244	Hydrogen Bonds	Arg50
Val135	Non-Bonded Contacts	Phe57
Met136	Non-Bonded Contacts	Tyr54
Ser137	Non-Bonded Contacts	Tyr54
Gly138	Non-Bonded Contacts	Tyr54
Glu141	Non-Bonded Contacts	Tyr102
Leu145	Non-Bonded Contacts	Arg50
Leu145	Non-Bonded Contacts	Phe57
His146	Non-Bonded Contacts	Arg50
His146	Non-Bonded Contacts	Tyr59
Leu147	Non-Bonded Contacts	Phe57
Gln148	Non-Bonded Contacts	Tyr59
Gln148	Non-Bonded Contacts	Lys65
Asp151	Non-Bonded Contacts	Phe57
Asp151	Non-Bonded Contacts	Thr58
Gln154	Non-Bonded Contacts	Gly55
Gln154	Non-Bonded Contacts	Phe57
Ala243	Non-Bonded Contacts	Phe99
Glu244	Non-Bonded Contacts	Phe99
Glu244	Non-Bonded Contacts	Arg50
Asn245	Non-Bonded Contacts	Trp52
Met246	Non-Bonded Contacts	Gly101
Pro247	Non-Bonded Contacts	Tyr102
Glu244	Salt Bridges	Arg50
		**Ab - L Chain**
Gln242	Hydrogen Bonds	Trp31
Gln242	Hydrogen Bonds	Ser92
Gln242	Hydrogen Bonds	Ser30
Gly256	Hydrogen Bonds	Trp31
Gln257	Hydrogen Bonds	Thr51
Gln257	Hydrogen Bonds	Ser50
Gln130	Non-Bonded Contacts	Asp28
His146	Non-Bonded Contacts	Asn93
His146	Non-Bonded Contacts	Phe94
His146	Non-Bonded Contacts	Ser92
Gln242	Non-Bonded Contacts	Tyr91
Gln242	Non-Bonded Contacts	Ser92
Gln242	Non-Bonded Contacts	Gly32
Gln242	Non-Bonded Contacts	Ser30
Gln242	Non-Bonded Contacts	Trp31
Gln242	Non-Bonded Contacts	Val29
Ala243	Non-Bonded Contacts	Tyr91

Glu244	Non-Bonded Contacts	Tyr91
Glu244	Non-Bonded Contacts	Phe94
Gly256	Non-Bonded Contacts	Trp31
Gln257	Non-Bonded Contacts	Trp31
Gln257	Non-Bonded Contacts	Ser50
Gln257	Non-Bonded Contacts	Thr51

Table S30. AAL160:Ag (7Z4T) interactions in the best Blind and HCDR_ESSA docking pose.

7Z4T HCDR_ESSA & Blind		
**Ag**	**Interaction Type**	**Ab - H Chain**
Pro23	Hydrogen Bonds	Trp33
Tyr24	Hydrogen Bonds	Tyr99
Glu64	Hydrogen Bonds	Ser54
Lys65	Hydrogen Bonds	Asp55
Lys65	Hydrogen Bonds	Asp57
Asn66	Hydrogen Bonds	Tyr52
Asn66	Hydrogen Bonds	Thr30
Gln81	Hydrogen Bonds	Asn101
Leu82	Hydrogen Bonds	Asn101
Val85	Hydrogen Bonds	Ser31
Met20	Non-Bonded Contacts	Asp57
Met20	Non-Bonded Contacts	Arg59
Pro23	Non-Bonded Contacts	Trp33
Tyr24	Non-Bonded Contacts	Thr100
Tyr24	Non-Bonded Contacts	Asn101
Tyr24	Non-Bonded Contacts	Trp33
Tyr24	Non-Bonded Contacts	Tyr99
Tyr24	Non-Bonded Contacts	Tyr32
Lys65	Non-Bonded Contacts	Ser54
Lys65	Non-Bonded Contacts	Tyr52
Lys65	Non-Bonded Contacts	Asp55
Lys65	Non-Bonded Contacts	Asp57
Asn66	Non-Bonded Contacts	Tyr52
Asn66	Non-Bonded Contacts	Ser31
Asn66	Non-Bonded Contacts	Thr30
Asn66	Non-Bonded Contacts	Ser54
Leu67	Non-Bonded Contacts	Tyr52
Leu67	Non-Bonded Contacts	Trp33
Leu67	Non-Bonded Contacts	Ser31
Gln81	Non-Bonded Contacts	Asn101
Leu82	Non-Bonded Contacts	Asn101
Ser84	Non-Bonded Contacts	Ser31
Ser84	Non-Bonded Contacts	Tyr32
Val85	Non-Bonded Contacts	Ser31
Pro87	Non-Bonded Contacts	Ser28
Pro87	Non-Bonded Contacts	Ser31
Lys65	Salt Bridges	Asp55
Lys65	Salt Bridges	Asp57
		**Ab - L Chain**
Met20	Hydrogen Bonds	Trp94
Glu25	Hydrogen Bonds	Arg91
Lys27	Hydrogen Bonds	Asn93
Lys74	Hydrogen Bonds	Asp50
Met20	Non-Bonded Contacts	Trp94
Ser21	Non-Bonded Contacts	Trp94
Ser21	Non-Bonded Contacts	Asn93

Gly22	Non-Bonded Contacts	Arg91
Gly22	Non-Bonded Contacts	Asn93
Gly22	Non-Bonded Contacts	Trp94
Pro23	Non-Bonded Contacts	Trp94
Pro23	Non-Bonded Contacts	Phe96
Pro23	Non-Bonded Contacts	Arg91
Glu25	Non-Bonded Contacts	Arg91
Lys27	Non-Bonded Contacts	Asn93
Lys74	Non-Bonded Contacts	Tyr32
Lys74	Non-Bonded Contacts	Asp50
Pro131	Non-Bonded Contacts	Ser30
Glu25	Salt Bridges	Arg91
Lys74	Salt Bridges	Asp50

Table S31. AAL160:Ag (7Z4T) interactions in the best Ag_ESSA docking pose.

7Z4T Ag_ESSA		
**Ag**	**Interaction Type**	**Ab - H Chain**
Tyr24	Hydrogen Bonds	Trp33
Tyr24	Hydrogen Bonds	Tyr99
Lys65	Hydrogen Bonds	Asp55
Lys65	Hydrogen Bonds	Asp57
Asn66	Hydrogen Bonds	Tyr52
Asn66	Hydrogen Bonds	Thr30
Lys74	Hydrogen Bonds	Asn101
Leu82	Hydrogen Bonds	Asn101
Val85	Hydrogen Bonds	Ser31
Asp86	Hydrogen Bonds	Ser28
Lys88	Hydrogen Bonds	Ser28
Met20	Non-Bonded Contacts	Arg59
Pro23	Non-Bonded Contacts	Trp33
Tyr24	Non-Bonded Contacts	Tyr99
Tyr24	Non-Bonded Contacts	Thr100
Tyr24	Non-Bonded Contacts	Trp33
Tyr24	Non-Bonded Contacts	Tyr32
Glu64	Non-Bonded Contacts	Asp55
Lys65	Non-Bonded Contacts	Tyr52
Lys65	Non-Bonded Contacts	Asp55
Lys65	Non-Bonded Contacts	Asp57
Asn66	Non-Bonded Contacts	Tyr52
Asn66	Non-Bonded Contacts	Ser31
Asn66	Non-Bonded Contacts	Ser54
Asn66	Non-Bonded Contacts	Thr30
Leu67	Non-Bonded Contacts	Tyr52
Leu67	Non-Bonded Contacts	Trp33
Lys74	Non-Bonded Contacts	Asn101
Lys74	Non-Bonded Contacts	Trp102
Gln81	Non-Bonded Contacts	Asn101
Leu82	Non-Bonded Contacts	Asn101
Glu83	Non-Bonded Contacts	Asn101
Ser84	Non-Bonded Contacts	Ser31
Ser84	Non-Bonded Contacts	Tyr32
Val85	Non-Bonded Contacts	Ser31
Asp86	Non-Bonded Contacts	Ser31
Asp86	Non-Bonded Contacts	Ser28
Pro87	Non-Bonded Contacts	Ser31
Lys88	Non-Bonded Contacts	Ser28
Lys65	Salt Bridges	Asp55
Lys65	Salt Bridges	Asp57
		**Ab - L Chain**
Glu25	Hydrogen Bonds	Arg91
Lys27	Hydrogen Bonds	Asn93
Asp75	Hydrogen Bonds	Asn53
Ser21	Non-Bonded Contacts	Asn93
Ser21	Non-Bonded Contacts	Trp94
Gly22	Non-Bonded Contacts	Arg91

Gly22	Non-Bonded Contacts	Asn93
Gly22	Non-Bonded Contacts	Trp94
Pro23	Non-Bonded Contacts	Phe96
Pro23	Non-Bonded Contacts	Arg91
Pro23	Non-Bonded Contacts	Trp94
Glu25	Non-Bonded Contacts	Arg91
Lys27	Non-Bonded Contacts	Asn93
Lys74	Non-Bonded Contacts	Tyr32
Lys74	Non-Bonded Contacts	Asp50
Asp75	Non-Bonded Contacts	Ser52
Asp75	Non-Bonded Contacts	Asn53
Glu25	Salt Bridges	Arg91
Lys74	Salt Bridges	Asp50

Table S32. Selected essential residues for Max and Ave Epitopes. Bold residues represent seeds for that epitope.

	Max_Epitope			Average_Epitope		
**PDB_ID**	**Epitope 1**	**Epitope 2**	**Epitope 3**	**Epitope 1**	**Epitope 2**	**Epitope 3**
**3WD5**	**R6**, T7	S9, D10, K11, A38, **N39**	F64, K65, L76, K112, **W114**, Y115, E116, Y141	SAME AS MAX		
**4G3Y**	L142, **F144**	F64, Q67, C69, L76, K112, **W114**, Y141	E104, **T105**, P106, E107	SAME AS MAX		V17, **W28**, N46, P152
**5Y9J**	**T143**, E144, D145, L285	R214, **K216**, H218, V219, E223, L224, S225, D257	**F220**, D222	H218, V219, **F220**, D222, E223	SAME AS MAX_EP1	R214, **K216**, L224, S225, D257
**5GGT**	F19, **Y118**	I38, M59, I64, Y81, **Y112**	**F42**	SAME AS MAX	SAME AS MAX_EP3	SAME AS MAX_EP2
**7C88**	I64, Y81, I101, **Y112**	**F42**	I38, Y56, **W57**, L99	SAME AS MAX_EP2	SAME AS MAX_EP1	SAME AS MAX_EP3
**1OAK**	R511, L512, L513, R545, K549, **W550**, R552, R611	F606, **R616**, I617, I647	L515, V516, **F517**, L518, L619	SAME AS MAX		
**3S36**	L244, **W260**, Y262, H269, V273, S290, L292	**Y221**, N253, V254	I294, **Y305**	SAME AS MAX		
**4DGI**	I139, F141, Y149, **Y150**, Y157, M205, V209	Y163, R164, **F175**, Y218	M206, E207, **R208**, V210, E211, Q212	SAME AS MAX		
**7E9B**	W32, **F56**, F63	A80, **Q91**, D92, F95	F37, N66, **W67**, Y68, K78, Y121	**W32**, F56, F63, K135	F37, N66, **W67**, Y68, K78, A80, F95, Y121	**Q91**, D92
**8Y31**	**Y244**, F293, F298, F324, T326	W261, L262, Y275, **W277**, L304, R306, E321	**Y250**, Y252, M255	SAME AS MAX_EP1	**W261**, L262, H263, W277, Y303, L304	SAME AS MAX_EP3
**4G6J**	**Y68**, L69, Q81, L82, Y90, P91, F99	N7, L10, L18, V40, **F42**, M44, L62	C71, L80, **F101**, K103, I122	SAME AS MAX_EP1	SAME AS MAX_EP3	SAME AS MAX_EP2
